# The genomic basis of circadian and circalunar timing adaptations in a midge

**DOI:** 10.1038/nature20151

**Published:** 2016-11-21

**Authors:** Tobias S. Kaiser, Birgit Poehn, David Szkiba, Marco Preussner, Fritz J. Sedlazeck, Alexander Zrim, Tobias Neumann, Lam-Tung Nguyen, Andrea J. Betancourt, Thomas Hummel, Heiko Vogel, Silke Dorner, Florian Heyd, Arndt von Haeseler, Kristin Tessmar-Raible

**Affiliations:** 1 Max F. Perutz Laboratories, University of Vienna, Campus Vienna Biocenter, Dr. Bohr-Gasse 9/4, Vienna, A-1030 Austria; 2 Center for Integrative Bioinformatics Vienna, Max F. Perutz Laboratories, University of Vienna and Medical University of Vienna, Dr. Bohr-Gasse 9, Vienna, A-1030 Austria; 3 Research Platform ‘Rhythms of Life’, University of Vienna, Vienna, A-1030 Austria; 4 Department of Biology, Chemistry, Pharmacy, Institute of Chemistry and Biochemistry, FU Berlin, D-14195 Berlin Germany; 5 Bioinformatics and Computational Biology, Faculty of Computer Science, University of Vienna, Vienna, A-1030 Austria; 6 Department of Biomedical Sciences, Institute of Population Genetics, University of Veterinary Medicine Vienna, Josef-Baumann-Gasse 1, Vienna, A-1210 Austria; 7 Department of Neurobiology, Faculty of Life Sciences, University of Vienna, Vienna, A-1090 Austria; 8 Department of Entomology, Max Planck Institute for Chemical Ecology, Hans-Knöll-Straße 8, Jena, D-07745 Germany; 9 Present Address: †Present addresses: Max Planck Institute for Evolutionary Biology, August-Thienemann-Straße 2, D-24306 Plön, Germany (T.S.K.); Department of Computer Science, Johns Hopkins University, Baltimore, Maryland 21211, USA (F.J.S.)., ,

**Keywords:** Genome, Evolutionary genetics, Ecological genetics, Circadian regulation

## Abstract

Organisms use endogenous clocks to anticipate regular environmental cycles, such as days and tides. Natural variants resulting in differently timed behaviour or physiology, known as chronotypes in humans, have not been well characterized at the molecular level. We sequenced the genome of *Clunio marinus*, a marine midge whose reproduction is timed by circadian and circalunar clocks. Midges from different locations show strain-specific genetic timing adaptations. We examined genetic variation in five *C. marinus* strains from different locations and mapped quantitative trait loci for circalunar and circadian chronotypes. The region most strongly associated with circadian chronotypes generates strain-specific differences in the abundance of calcium/calmodulin-dependent kinase II.1 (CaMKII.1) splice variants. As equivalent variants were shown to alter CaMKII activity in *Drosophila melanogaster*, and *C. marinus* (*Cma*)-CaMKII.1 increases the transcriptional activity of the dimer of the circadian proteins *Cma-*CLOCK and *Cma-*CYCLE, we suggest that modulation of alternative splicing is a mechanism for natural adaptation in circadian timing.

**Supplementary information:**

The online version of this article (doi:10.1038/nature20151) contains supplementary material, which is available to authorized users.

## Abstract (web summary)

Genomic and molecular analyses of *Clunio marinus* timing strains suggest that modulation of alternative splicing of Ca2+/calmodulin-dependent kinase II represents a mechanism for evolutionary adaptation of circadian timing.

**Supplementary information:**

The online version of this article (doi:10.1038/nature20151) contains supplementary material, which is available to authorized users.

## Night and day habits of a sea midge

Kristin Tessmar-Raible and colleagues report the genome of *Clunio marinus*, a marine midge whose reproduction is timed to the tides by circadian and circalunar clocks. To identify genetic variation associated with timing differences, the authors report genetic mapping in a selection of *C. marinus* strains with a range of circadian and circalunar timing. They suggest that circalunar and circadian timing are regulated by separate pathways, do not find involvement of core clock genes, and implicate calcium/calmodulin-dependent kinase II.1 in the regulation of circadian timing.

**Supplementary information:**

The online version of this article (doi:10.1038/nature20151) contains supplementary material, which is available to authorized users.

## Main

**Extended Data Figure 1:**
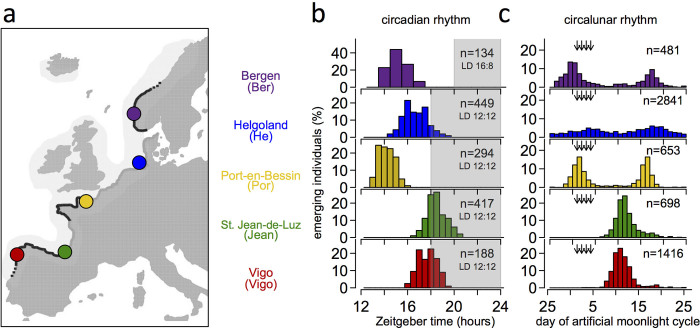
The biology of *Clunio marinus*. **a**, *C. marinus* is restricted to rocky shores (black lines), the localities differing in tidal regime (adapted from ref. 67). **b**, **c**, Local strains show corresponding genetic adaptations in their circadian^67^ (**b**) and circalunar rhythms (**c**, He^1^, Jean^5^). Timing was measured in the laboratory under artificial moonlight (arrows in **c**) in a 30-day cycle and a light–dark cycle of 12:12 (He, Por, Jean, Vigo) or 16:8 (Ber). Seasonal differences in daily illumination duration do not affect circadian emergence peaks^1,76^. Historically, for *C. marinus* ‘zeitgeber time 0’ is defined as the middle of the dark phase.

Around the new or full moon, during a few specific hours surrounding low tide, millions of non-biting midges of the species *C. marinus* emerge from the sea to perform their nuptial dance. Adults live for only a few hours, during which they mate and oviposit. They must therefore emerge synchronously and—given that embryonic, larval and pupal development take place in the sea—at a time when the most extreme tides reliably expose the larval habitat. The lowest low tides occur predictably during specific days of the lunar month at a specific time of day. Consequently, adult emergence in *C. marinus* is under the control of circalunar and circadian clocks^1,2^. Notably, although the lowest low tides recur invariably at a given location, their timing differs between geographic locations^3^. Consequently, *C. marinus* strains from different locations (Extended Data Fig. 1a) show local adaptation in circadian and circalunar emergence times (Extended Data Fig. 1b, c). Crosses between the Jean and Por strains showed that the differences in circadian and circalunar timing are genetically determined^4,5^ and largely explained by two circadian and two circalunar quantitative trait loci (QTLs)^6^.

Studies on timing variation or chronotypes in animals and humans have often focused on candidate genes from the circadian transcription–translational oscillator. In *D. melanogaster*, polymorphisms in the core circadian clock genes *period*, *timeless* and *cryptochrome* are associated with adaptive differences in temperature compensation^7^, photo-responsiveness of the circadian clock^8^ and emergence rhythms^9^. While these studies offer insights into the evolution of known circadian-clock molecules, genome-wide association studies^10,11^ and other forward genetic approaches (reviewed in ref. 12) are essential to provide a comprehensive, unbiased assessment of natural timing variation, for instance underlying human sleep-phase disorders. While the adaptive nature of human chronotypes remains unclear, the chronotypes of *C. marinus* represent evolutionary adaptations to their habitat. Our study aimed to identify the genetic basis of *C. marinus* adaptation to its specific ecological ‘timing niche’. In addition, the genetic dissection of adaptive natural variants of non-circadian rhythms^13^, as also present in *C. marinus,* may provide an entry point into their unknown molecular mechanisms.

As a starting point for these analyses, we sequenced, assembled, mapped and annotated a *C. marinus* reference genome.

## The *Clunio* genome and QTLs for timing

**Figure 1:**
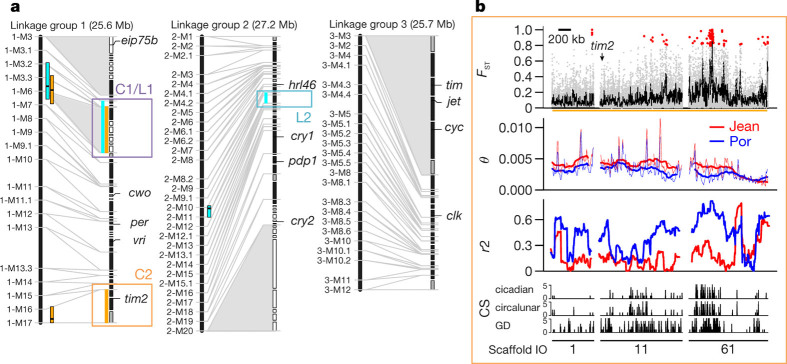
Identification of candidate regions in the timing QTLs by combined genetic and molecular maps. **a**, The three linkage groups of *C. marinus* with reference scaffolds (right) anchored on a genetic linkage map (left). Scaffolds which are ordered and oriented, black bars; not oriented, grey bars; neither ordered nor oriented, white bars. Grey shadings, large non-recombining regions. QTLs, circadian (orange), circalunar (cyan). One circadian and circalunar QTL overlap, resulting in three physical QTL regions (C1/L1, C2 and L2, in purple, orange and cyan, respectively). **b**, Population genomic analysis of QTL C2. Analysis of Por and Jean strains (in blue and red, respectively, in middle two panels). Top panel, genetic differentiation for single SNPs (red dots) and in 5-kb windows (black line). Second panel, genetic diversity (*θ*) in 20-kb (thin line) and 200-kb (thick line) windows. Third panel, linkage disequilibrium (*r*^2^) in 100-kb windows. Bottom panel, correlation score (CS) for genetic differentiation with values for circadian timing (top), circalunar timing (middle) and geographic distance (bottom) for Vigo, Jean, Por, He and Ber strains. Bottom numbers, scaffold IDs. For further details, including QTLs C1/L1 and L2, see Extended Data Fig. 5a, b. PowerPoint slide

**Extended Data Figure 2:**
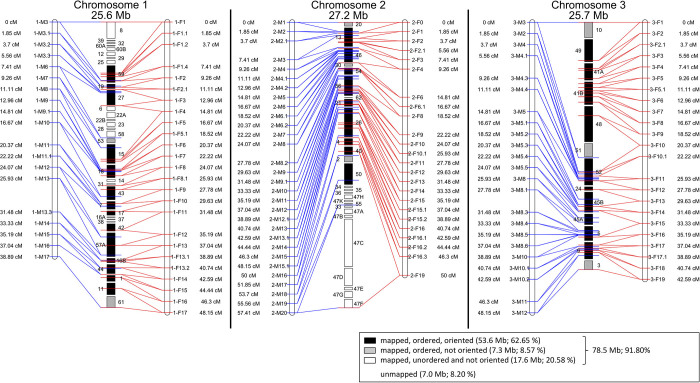
The reconstructed chromosomes of *C. marinus* based on the genetic linkage map. Left map, male informative markers. Right map, female informative markers. See Fig. 1a legend for further details.

**Extended Data Figure 3:**
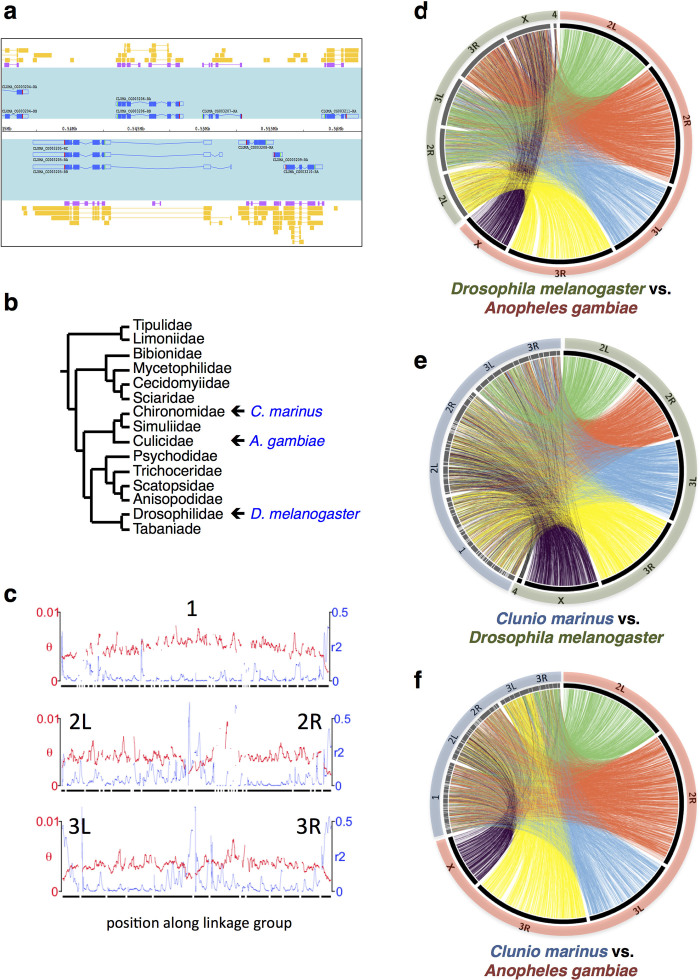
*C. marinus* genome characterization. **a**, Representative genomic region with densely packed gene models (super-scaffold 1, 535–565 kb). Gene models are shown in blue on a turquoise background. Gene predictions (SNAP) are shown in purple. Transcript evidence is shown in yellow. **b**, Phylogenetic relationships of *C. marinus to* other Diptera (according to ref. 77). **c**, Genetic diversity (*θ*; red) and linkage disequilibrium (*r*^2^; blue) of the Jean strain plotted for the three *C. marinus* linkage groups, revealing characteristic signatures of telomeres and centromeres. **d**–**f**, Synteny comparisons among the genomes of *C. marinus*, *A. gambiae* and *D. melanogaster* based on 5,388 1:1:1 orthologues.

Our reference genome CLUMA_1.0 of the Jean laboratory strain contained 85.6 Mb of sequence (Table 1), close to the previous flow-cytometry-based estimate of 95 Mb^6^, underlining that chironomids generally have small genomes^14,15,16^. The final assembly has a scaffold N50 of 1.9 Mb. Genome-wide genotyping of a mapping family with restriction-site associated DNA sequencing allowed 92% of the reference sequence to be consistently anchored along a genetic linkage map (Fig. 1a and Extended Data Fig. 2), improving the original linkage map (Supplementary Methods 5). Automated genome annotation resulted in 21,672 gene models. Protein similarity and available transcripts support 14,041 gene models (Supplementary Table 1), within the range of gene counts for *D. melanogaster* (15,507) and *Anopheles gambiae* (13,460). Thus, the very small *C. marinus* genome appears to be complete (Table 1, Extended Data Fig. 3, Supplementary Note 1 and Supplementary Table 2). The *C. marinus* reference genome makes chironomids the third dipteran subfamily with an annotated genome reconstructed to chromosome scale (Fig. 1a and Extended Data Figs 2, 3b–f).

**Table 1:** Comparison of the *C. marinus* genome assembly with published model insect genomes

	***Clunio marinus***	***Danaus plexippus***^43^	***Tribolium castaneum***^44^	***Apis mellifera***^45^
Total bases (Mb)	86	278	160	236
Mapped sequence (%)	92	NA	90	79
Scaffold N50 (Mb)	1.9	0.2	1	0.4
Contig N50 (kb)	79	50	41	41
AT content (%)	68.3	68.4	67	66
Completeness (%)	98.0	98.5	NA	NA
Platform	Illumina	Illumina + 454	Sanger + BAC	Sanger + BAC

**Extended Data Figure 4:**
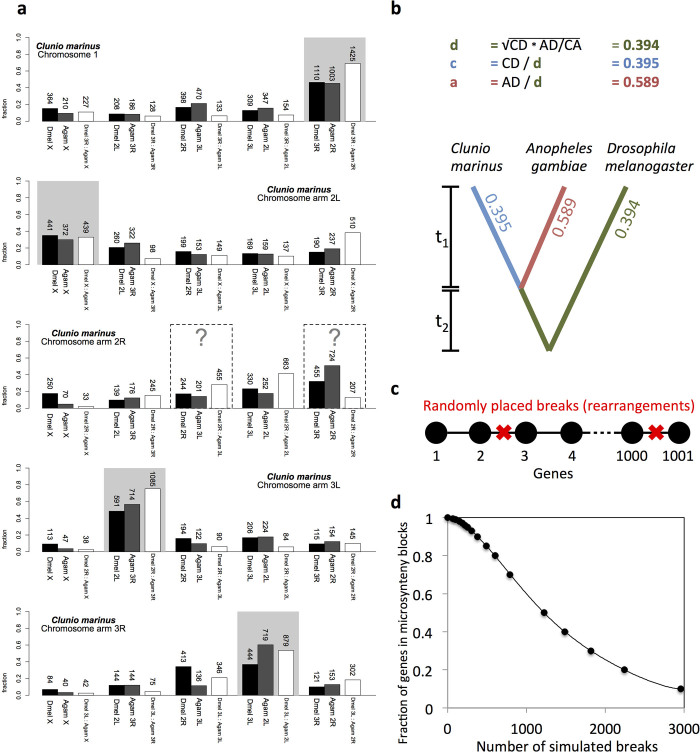
Synteny analyses of *C. marinus* chromosome arms. **a**, Gene content of the *C. marinus* chromosome arms relative to the chromosome arms of *D. melanogaster* (black bars) and *A. gambiae* (grey bars). The very small chromosome 4 of *D. melanogaster* is neglected. Chromosome arms of *D. melanogaster* and *A. gambiae* are paired according to their published homology^78^. For four of the chromosome arms of *C. marinus* the homologous arms in *D. melanogaster* and *A. gambiae* are identified (grey shading). For comparison, the conservation of the identified *D. melanogaster* and *A. gambiae* homologues to each other is given by plotting the gene content of the homologous *D. melanogaster* chromosome arm relative to the different chromosome arms of *A. gambiae* (white bars). The numbers of orthologous genes considered in each comparison are given above the bars. For chromosome arm 2R of *C. marinus* the homologies are unclear. Possibly, chromosome arm 2R of *C. marinus* has undergone so many re-arrangements with other chromosome arms that it is no longer recognizable, which is consistent with complex polymorphic re-arrangements in this chromosome arm of *C. marinus* (see Supplementary Note 3). **b**, Microsynteny is analysed relative to *D. melanogaster* and *A. gambiae*, based on 5,388 1:1:1 orthologues. The fraction of genes in conserved microsynteny blocks is calculated and distributed along the phylogenetic tree. **c**, **d**, A simulation was used to estimate how many chromosomal re-arrangements are required to produce the observed degree of microsyntenic conservation (for details see Supplementary Note 3).

We performed a basic genome characterization and comparison to other dipterans. We delineated the five *C. marinus* chromosome arms (Supplementary Note 2, Extended Data Fig. 3c and Supplementary Table 3) and homologized them to *D. melanogaster* and *A. gambiae* by synteny comparisons (Extended Data Figs 3 and 4, Supplementary Note 2 and Supplementary Table 3). We also found the ZW-like sex-linked locus in *C. marinus*^6^ outside the X chromosome homologue (Supplementary Note 2) and detected an elevated rate of chromosomal re-arrangement (Fig. 1a, Supplementary Note 3 and Extended Data Figs 2, 3b–f, 4). Taken together, the *C. marinus* reference genome appears well assembled.

As the next step towards identifying the molecular basis of circadian and circalunar timing adaptations in *C. marinus*, we refined the previously identified timing QTL positions^6^ based on the new high-density, restriction-site-associated DNA sequencing markers (Supplementary Table 4 and Supplementary Note 4) and determined the reference sequence corresponding to the QTL confidence intervals (Fig. 1, orange and cyan bars; and Supplementary Table 4). None of the core circadian clock genes were found to be located within these QTLs (Fig. 1a). Only *timeout/timeless2*, a *timeless* homologue with a minor role in circadian clock resetting^17^, is located within the QTLs.

## Genetic variation in *Clunio* timing strains

**Extended Data Figure 5:**
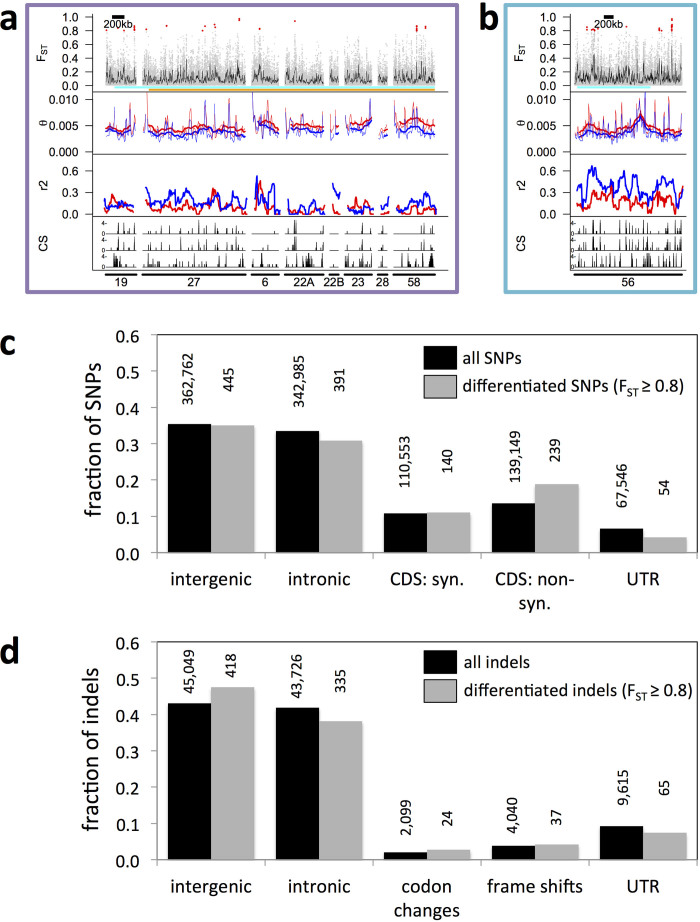
Population genomic analysis of QTLs C1/L1 and C2 and genome-wide analysis of locations and putative effects of SNPs and indels. **a**, **b**, Population genomic analysis of QTLs C1/L1 and C2. Panels 1–3: Por versus Jean strains in blue and red, respectively, in panel 2 and 3. From top to bottom, panel 1, genetic differentiation (red dots, SNPs with *F*_ST_ ≥ 0.8; grey dots, *F*_ST_ < 0.8; black line, average *F*_ST_ in 5-kb sliding windows). Panel 2, genetic diversity (*θ*) in 20-kb (thin line) and 200-kb (thick line) windows. Panel 3, linkage disequilibrium (*r*^2^) for SNP pairs 0–600 bp apart in 100-kb windows (step size: 5 kb). Panel 4, correlation score (CS; 0–5) for genetic differentiation with circadian timing (top), circalunar timing (middle) and geographic distance (bottom) for five European *C. marinus* strains (Vigo, Jean, Por, He and Ber). Bottom numbers, scaffold IDs. See also Fig. 1. **c**, **d**, Locations and putative effects of SNPs (**c**) and indels (**d**) with respect to the annotated gene models. The fractions of SNPs or indels in each category are compared for all SNPs and indels (black bars) versus differentiated SNPs and indels (*F*_ST_ ≥ 0.8 between Por and Jean strains; grey bars). Absolute numbers are given above the bars. In gene models with several splice forms, SNPs and indels can have different effects, for example, ‘CDS, non-synonymous’ for one splice form and ‘intronic’ for another splice form. Therefore, the sum across locations is slightly larger than the actual numbers of SNPs and indels. ‘Codon changes’ are all codon insertions or deletions that do not result in frame shifts beyond the actual insertion/deletion site. CDS, coding sequence; syn., synonymous; non-syn., non-synonymous; UTR, untranslated region.

We then re-sequenced the Por and Jean strains (Extended Data Fig. 1), for which the initial QTL analysis was performed^6^. Two pools of 300 field-caught individuals were sequenced at >240× coverage (Supplementary Table 5). Mapping reads against the reference genome identified 1,010,052 single nucleotide polymorphisms (SNPs), 72% of which were present in both the Por and Jean strains. Based on all SNPs we determined genetic differentiation (*F*_ST_), genetic diversity (*θ*) and short-range linkage disequilibrium (measured as *r*^2^) (Fig. 1b and Extended Data Figs 3c, 5a, b).

Genome-wide genetic differentiation between the Por and Jean strains is moderate (*F*_ST_ = 0.11), providing a good basis for screening the genome for local timing adaptation based on genetic divergence. According to QTL analysis, the two circadian QTLs explain 85% of the daily timing difference and the two circalunar QTLs explain the entire monthly timing difference (Supplementary Table 4 and ref. 6). As each locus therefore has a strong effect on timing, selection against maladapted alleles must be strong and timing loci should be strongly differentiated.

Within the confidence intervals of the QTLs, 158 SNPs and 106 indels (insertions or deletions) are strongly differentiated (*F*_ST_ ≥ 0.8; Fig. 1b and Extended Data Fig. 5; SNPs, red dots in *F*_ST_ panels, for genome-wide comparison see Supplementary Note 5). We compiled a list of candidate genes for circadian and circalunar timing adaptations based on their proximity to differentiated SNPs and indels in the QTLs (Supplementary Table 6). The candidate genes neither comprise core circadian clock genes (*timeless2/timeout*, max. *F*_ST_ ≤ 0.5; average *F*_ST_ = 0.07), nor are enriched for any particular pathway (gene ontology-term analysis; Supplementary Table 7).

## Timing phenotype with genotype correlation

Assuming that the alleles associated with timing adaptation probably originated from standing genetic variation (Supplementary Note 5), genetic variation at timing loci should not vary freely between strains, but rather strains with similar timing should share functionally relevant alleles. To identify such loci, we extended the genomic screen to three additional strains: from Vigo (Vigo), Helgoland (He) and Bergen (Ber; Extended Data Fig. 1 and Supplementary Tables 5, 8). We then tested all five sequenced strains for correlations between genetic differentiation (*F*_ST_) and timing differences, or geographic distances as a null model (Supplementary Table 8).

Overall, genome-wide genetic differentiation was not correlated with circadian (*r* = 0.10, *P* = 0.31) or circalunar (*r* = 0.56, *P* = 0.12) timing differences, but with geographic distance (‘isolation by distance’; *r* = 0.88, *P* = 0.008). Against this genomic background signal of isolation by distance, we screened the genome in 5-kb sliding windows for peaks of correlation between genetic differentiation and timing, resulting in a correlation score (Fig. 1b and Extended Data Fig. 5a, b, CS panels, score ranging from 0 to 5; for details see Methods). Combining the evidence from the Por versus Jean strain *F*_ST_ screen (Supplementary Table 6) with these patterns of correlation between timing and genetic divergence reduced the candidate gene list to 49 genes (Supplementary Table 9).

Of particular note, a single region in circadian QTL C2 was strikingly differentiated (Fig. 1b). In this region, linkage disequilbrium in the Por strain was significantly elevated (permutation test; *P* = 0.002), and genetic diversity significantly decreased in some stretches (permutation test; *P* = 0.037 and 0.020), compared to the Por genome average. This may indicate a recent episode of selection in Por, potentially during timing adaptation, as this region is also strongly enriched for timing-correlated polymorphisms (Fig. 1b, CS panel). The most extreme values of genetic differentiation, genetic diversity and timing correlation localize to the *CaMKII.1* locus and the anterior section of a gene homologous to the *big bang (bbg)* gene.

## CaMKII affects the circadian core clock

Not only does the *CaMKII.1* locus harbour the highest number of differentiated polymorphisms (Supplementary Table 9), but CaMKII has also been shown to affect circadian timing. Mouse CaMKIIα phosphorylates CLOCK and facilitates its dimerization with BMAL *in vivo*^18^. Mice with inactive, kinase-dead CaMKIIα^K42R^ have dampened circadian rhythms and a lengthened circadian free-running period^18^. CaMKII also phosphorylates the CLOCK protein^19^ in the *D. melanogaster* S2 cell line, and *in vivo* inhibition of *Dme-*CaMKII in a sensitized background with reduced Ca^2+^ levels lengthens the circadian free-running period^20^, suggesting that the role of CAMKII in circadian timing is conserved across animals.

**Figure 2:**
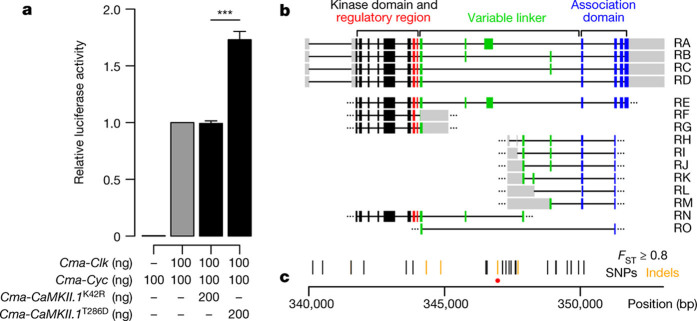
CaMKII.1 regulates Clk and Cyc transcriptional activity and exhibits strain specific splice variants. **a**, Additional *C. marinus* CaMKII.1 increases the transcriptional activity of *C. marinus Clk* and *Cyc* in a *D. melanogaster* S2 cell luciferase assay using the 3X69 E-box containing enhancer (*period* 3X69–*luc* (ref. 21)). Data are represented as mean ± s.e.m.; two-sided Welch two-sample *t*-test; biological replicates, *n* = 5, except for no *clk* control, *n* = 3, each biological replicate represents the average of three preparation replicates. ***P < 0.0005. **b**, Exons of full (RA–RD) and partial (RE–RO) *Cma-CaMKII.1* transcripts. **c**, Distribution of SNPs (black), indels (orange) and a 125-bp insertion (red dot) along the *Cma-CaMKII.1* locus, all with *F*_ST_ ≥ 0.8. PowerPoint slide

**Extended Data Figure 6:**
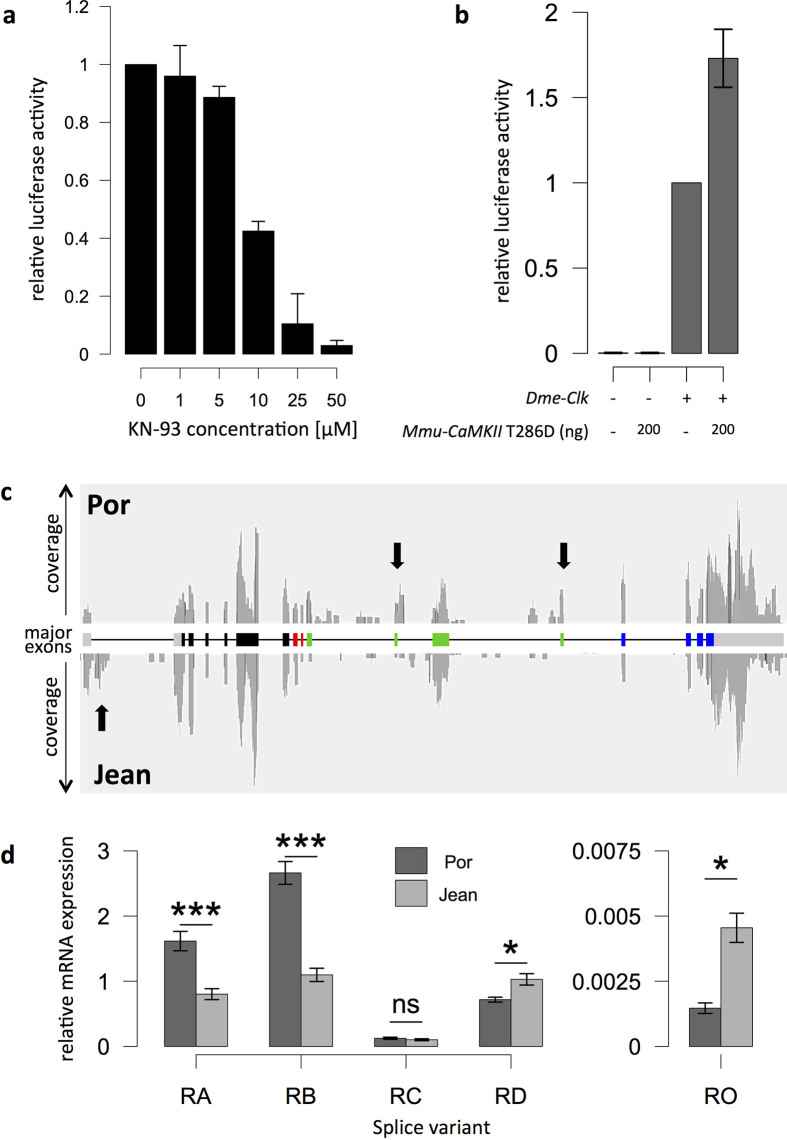
CaMKII regulates CLK/CYC transcriptional activity and exhibits strain-specific splice variants. **a**, Quantification of luciferase activity under the control of an artificial 3X69 E-box containing enhancer in *D. melanogaster* S2 cells. Increasing amounts of the CaMKII inhibitor KN-93 decrease luciferase activity in a concentration-dependent manner, providing evidence that endogenous CaMKII activity regulates the transcriptional activity of *D. melanogaster* CLOCK-CYCLE. **b**, Without co-transfection of *D. melanogaster clock*, there is no detectable luciferase activity. The constitutively active form of CaMKII (mouse T286D) increases luciferase activity (normalized to CLOCK^+^; data are shown as mean ± s.e.m.; *n* = 4 biological replicates). **c**, RNA sequencing reads mapped to the *CaMKII.1* genomic locus. Arrows, major differences between the strains. **d**, Relative expression levels of the four major CaMKII.1 transcripts (RA–RD) and the minor variant RO in the Por and Jean strains of *C. marinus*, as measured by qPCR (data are shown as mean ± s.e.m.; two-sided Wilcoxon rank-sum test; ***P < 0.0005; *P < 0.05; NS, not significant; Holm correction for multiple testing; biological replicates, Por *n* = 9, Jean *n* = 10; except for RO: Por *n* = 3, Jean *n* = 8). RO was not detectable in six additional biological replicates of the Por strain, suggesting that the expression differences are even greater than currently estimated. Figure 3a shows the same data, normalized to the respective Por strain variants.

To determine whether CaMKII can also affect the circadian core clock in *C. marinus*, we tested the effect of *Cma*-CaMKII.1 in a cell-based assay using *D. melanogaster* S2 cells^19,21^. We repeated previous experiments^19^ showing that the chemical inhibition of endogenous *Dme*-CaMKII reduces the amount of generated luciferase (Extended Data Fig. 6a), whereas addition of a [Ca^2+^]-independent, and therefore constitutively active, variant of CaMKII (mouse, T286D) increases luciferase amounts (Extended Data Fig. 6b). Then we generated constructs for *C. marinus clock*, *C. marinus cycle*, and mutated kinase-dead (K42R) and [Ca^2+^]-independent (T286D) versions of *Cma-CaMKII.1*. Transfection of *Cma-clock* and *Cma-cycle* into *D. melanogaster* S2 cells leads to luciferase activity driven from the 3X69 promoter derived from the *Dme-period* promoter (Fig. 2a). The addition of [Ca^2+^]-independent *Cma-CaMKII.1*^T286D^ leads to a substantial increase in the luciferase signal (Fig. 2a), whereas addition of the kinase-dead *Cma*-*CaMKII.1*^K42R^ does not enhance luciferase activity (Fig. 2a). These data suggest that CaMKII kinase activity enhances E-box dependent transcription, as indicated by luciferase production driven by the *3X69*promoter, via the CLOCK–CYCLE dimer in *C. marinus*.

## *CaMKII.1* splicing correlates with timing

**Figure 3:**
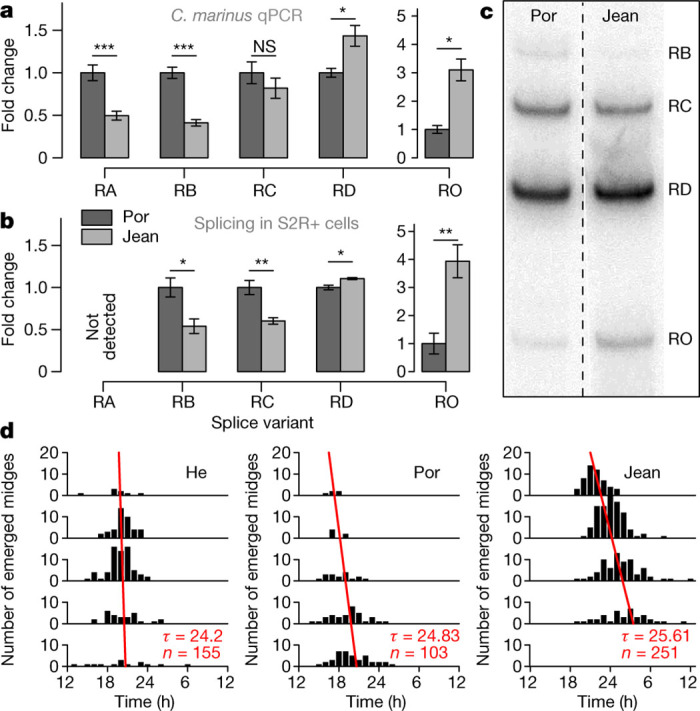
Differential CaMKII.1 splicing depends on sequence differences in the CaMKII.1 locus and correlates with endogenous circadian period lengths. **a**, qPCR values for *CaMKII.1* splice variants from Por and Jean strains, normalized to Por (for non-normalized data, see Extended Data Fig. 6d). Data are represented as mean ± s.e.m.; Por, *n* = 9 biological replicates; Jean, *n* = 10; RO, Por, *n* = 3; Jean *n* = 8; RO was not detected in six Por biological replicates, suggesting an even larger expression difference; two-sided Wilcoxon rank-sum test; *P < 0.05; **P < 0.005; ***P < 0.0005; NS, not significant; Holm correction for multiple testing. For RNA-seq data quantification see Extended Data Fig. 6c. **b**, Differential splicing of the *CaMKII.1* linker region in *D. melanogaster* S2R+ cells, normalized to Por, *n* = 7 biological replicates; two-sided two-sample *t*-test, otherwise as **a**. **c**, Representative phosphorimaging gel sections as quantified for **b**, two separate lanes from the same gel (for full gel, see Source Data). **d**, Free-running rhythm of adult emergence under constant dim white light (approximately 100 lx). He and Por share *CaMKII.1* alleles, while Jean has the other allele. To calculate the free-running period, time between subsequent emergence peaks was averaged, weighting each peak by the number of individuals. PowerPoint slide

**Extended Data Figure 7:**
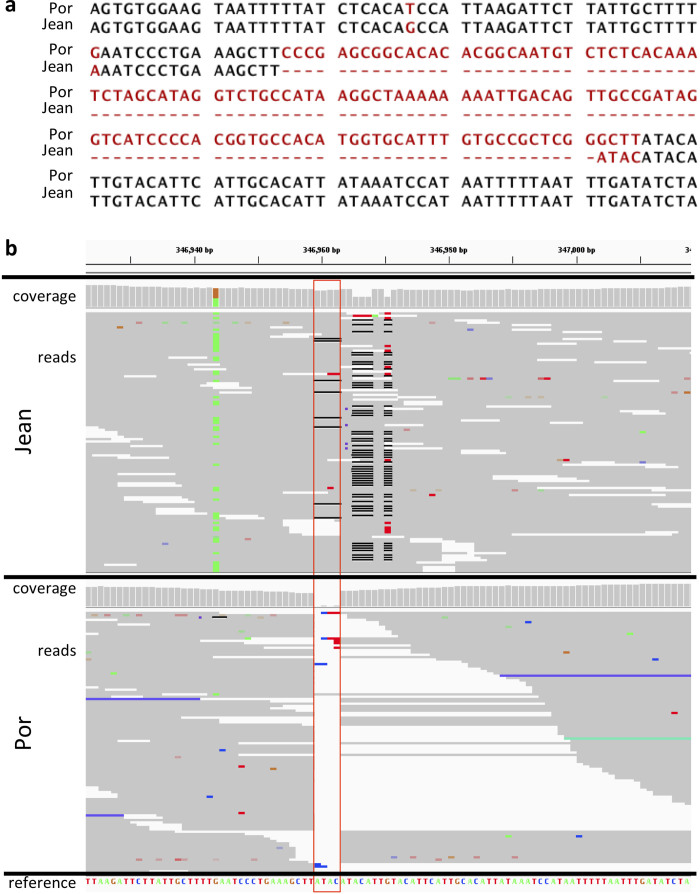
A differentiated 125-bp insertion in the CaMKII locus. **a**, Alignment of the part of the CaMKII locus of the Por and Jean strains that carries a 125-bp insertion in the Por strain. **b**, Pool–seq reads (>150× coverage) of this position for Por and Jean, as shown in the integrated genome viewer (IGV). The reference genome does not have the 125-bp insertion. At the position marked by the red box, the Jean strain has a 4-bp polymorphic indel (ATAC, frequently misaligned due to a SNP 8 bp downstream), whereas the Por strain has the 125-bp insertion (but not the 4-bp ATAC insertion). In Jean all reads span the indel, suggesting that if the 125-bp insertion is present in Jean at all, its frequency is very low. In contrast, in Por all reads but one end at this position, suggesting the frequency of the 125-bp insertion in Por is 154 of 155 reads or >0.99.

We then investigated how polymorphisms in the *Cma-CaMKII.1* locus affect the enzyme. We found two *CaMKII.1* alleles: one in the early emerging Por, He and Ber strains, and another in the late emerging Jean and Vigo strains. Most strain-specific polymorphisms are located in introns (Fig. 2b, c and Supplementary Table 9). If these polymorphisms were meaningful, then they should affect *CaMKII.1* expression and/or splicing. *Cma-CaMKII.1* has four functional domains^22^ (Fig. 2b). The majority of differentiated polymorphisms cluster in the region of the variable linker domain (Fig. 2b, c), including a 125-bp insertion (red dot in Fig. 2c; Extended Data Fig. 7). We identified four alternatively spliced full-length transcripts of *Cma-CaMKII.1* (RA–RD), which differ in the length of the linker (Fig. 2b). High-coverage RNA sequencing gave evidence for differential exon usage between the Jean and Por strains, as well as for previously non-annotated exons within the variable linker region (Extended Data Fig. 6c). PCR and Sanger sequencing confirmed several partial transcripts of additional splice variants of the linker region (RE–RO; Fig. 2b). We used transcript-specific qPCR to quantify all transcripts from third instar larvae. Generally, transcripts RE–RO are expressed at very low levels. Of those, only RO showed quantifiable expression differences between the Jean and Por strains (Fig. 3a and Extended Data Fig. 6d). Importantly, transcript-specific qPCR confirmed significant differential expression of the major transcripts in the Jean versus Por strains (Fig. 3a, Extended Data Fig. 6d), matching the RNA-sequencing (RNA-seq) data (Extended Data Fig. 6c). Consistently, variants with long linkers (RA, RB) showed higher expression in the Por strain, whereas shorter variants (RD, RO) showed higher expression in the Jean strain (Fig. 3a and Extended Data Fig. 6c, d).

If the detected differences in the abundance of *CaMKII.1* splice variants are associated with the timing differences, they should be directly caused by the strain-specific polymorphisms at the *CaMKII.1* locus. In order to test this, we generated minigenes that contained the alternatively spliced linker region of the *CaMKII.1* locus from either the Jean or Por strains. The two minigenes were transfected into cells of the *D. melanogaster* S2R+ cell line and expression of splice variants was analysed by radioactive RT–PCR (Fig. 3b, c). We detected four variants, corresponding to splice variants RB, RC, RD and RO. All variants showed the same strain-specific abundance differences in the S2R+ cell assay as in the *C. marinus* strains *in vivo* (Fig. 3a, b). As the cellular context is the same for both the Jean and Por minigenes in the S2R+ assay, *trans*-acting elements can be excluded as the cause of differential splicing, implying that it is a direct result of the genomic sequence differences at the *Cma-CaMKII.1* locus. While splice variants RB, RC and RD and their constituting exons are conserved in *D. melanogaster* (see Flybase annotations and ref. 23), a *D. melanogaster* RA counterpart does not exist. This may explain why this variant is undetectable in S2R+ cells (Fig. 3b).

## From splice variants to timing differences

CaMKII linker-length variants have been investigated in several species. *D. melanogaster* CaMKII isoforms corresponding to the RB, RC and RD variants of *C. marinus* have different substrate affinities and rates of target phosphorylation^23^. These activity differences are explained by the fact that CaMKII functions as a dodecamer, and the linker length determines the compactness and thus the substrate accessibility of the holoenzyme—enzymes with long linkers have higher activity. This structure–function relationship is possibly universal, as it is conserved between humans and *C. elegans*^22,24^.

Inactivation or inhibition of CaMKII lengthens circadian periods in mouse and fruitflies^18,20^. A connection between circadian period length and phase of activity in light–dark cycles is known from mutations in *period* in *D. melanogaster*^25^ and human chronotypes^26^. These findings imply that in *C. marinus* the more active and more readily Ca^2+^-activated, long-linker *CaMKII.1* variants should advance adult emergence by shortening the circadian clock period. Indeed, we find that the early emerging Por and He strains, which possess the same long-linker biased CaMKII.1 alleles, have shorter free-running circadian clock periods than the late emerging Jean strain (Fig. 3d).

**Extended Data Figure 8:**
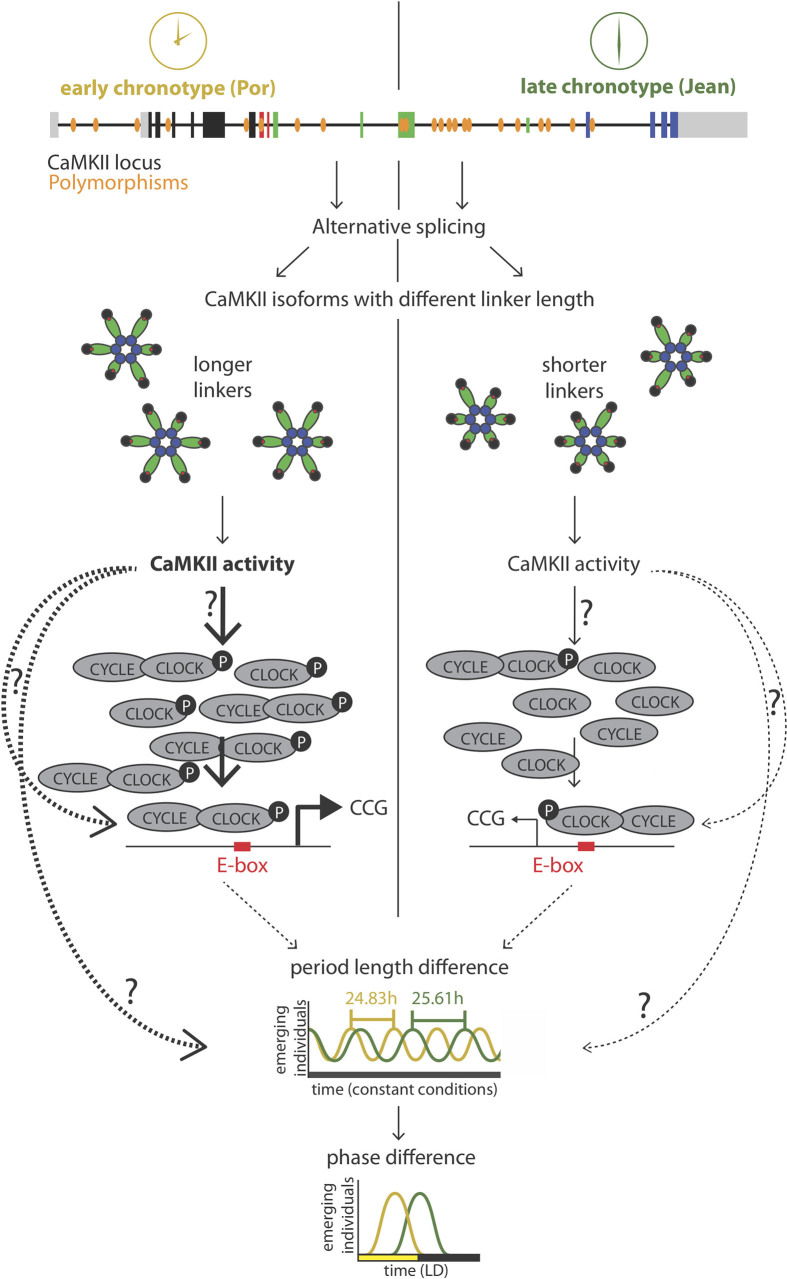
Model of circadian timing adaptation via sequence differences in the *CaMKII.1* genomic locus. Exon coloration as in Fig. 2b. The arrows with question marks indicate possible pathways that alone or in combination could mediate the effect of CaMKII.1 on timing. Dotted lines, indirect effects.

Integrating our results with those from the aforementioned literature, we propose that regulation of the ratio of *CaMKII.1* splice variants constitutes an evolutionary mechanism to adapt circadian timing (Extended Data Fig. 8): differences in the genomic sequence of *CaMKII.1* lead to differential *CaMKII.1* splicing and activity. Among a number of possible targets, this influences CLOCK–CYCLE dimer-dependent transcription, which in turn affects circadian period length and ultimately results in differences in adult emergence time.

## Discussion

Annual, lunar and tidal rhythms, as well as natural timing variation between individuals, are important and widespread phenomena that are poorly understood. The *C. marinus* reference genome and the genetic variation panel for five strains with differing circadian and circalunar timing establish new resources for further studies of these topics.

We identified *C. marinus* orthologues for all core circadian clock genes, none of which appear to be involved in circadian or circalunar timing adaptations. For circalunar timing, this supports the molecular independence of the circalunar clock from the circadian clock, as reported for *Platynereis dumerilii*^27^.

For circadian timing, strain-specific modulation in alternative splicing of *CaMKII.1* emerges as a possible mechanism for natural adaptation. In the light of previous experiments in *D. melanogaster* and mice^18,19,20,23^, it seems most likely that differences in CaMKII activity of the different splice forms lead to circadian timing differences via phosphorylation of CLOCK–CYCLE (Extended Data Fig. 8).

It is also conceivable that CaMKII affects circadian timing via other targets. For example, CaMKII is known to phosphorylate the cAMP response element binding protein (CREB)^28,29^. CREB is linked to the circadian clock by cAMP response elements (CRE) in the promoters of the *period* and *timeless* genes^30,31^, and by physical interaction of the CREB-binding protein (CBP) with CREB, CLOCK and CYCLE^32,33^. Furthermore, one of the most well-studied roles of CaMKII is the morphological modulation of neuronal plasticity and connectivity^34,35,36^. Such changes in connectivity have been increasingly implicated as part of the circadian timing mechanism in *D. melanogaster* and mammals^37^. Interestingly, the role of CaMKII in shaping neuronal connectivity has also been suggested to link to several neuropsychiatric diseases^38^, which often co-occur with chronobiological disruptions^39,40,41,42^. Further studies are needed to determine whether the modulation of CaMKII activity constitutes a molecular link between these phenomena.

## Methods

No statistical methods were used to predetermine sample size. The experiments were not randomized and the investigators were not blinded to allocation during experiments and outcome assessment.

### Animal culture and light regimes

The *C. marinus* laboratory stocks were bred according to Neumann^1^, care was provided by the MFPL aquatic facility. Briefly, *C. marinus* were kept in 20 × 20 × 5 cm plastic containers with sand and natural seawater diluted to 15‰ with desalted water, fed diatoms (*Phaeodactylum tricornutum*, strain UTEX 646) in early larval stages and nettle powder in later stages. Temperature in the climate chambers was set to 20 °C and the light–dark cycle was 12:12 (except where noted differently). Moonlight was simulated with an incandescent flashlight bulb (about 1 lx), which was switched on all night for four successive nights every 30 days.

### Genome assembly

**Extended Data Figure 9:**
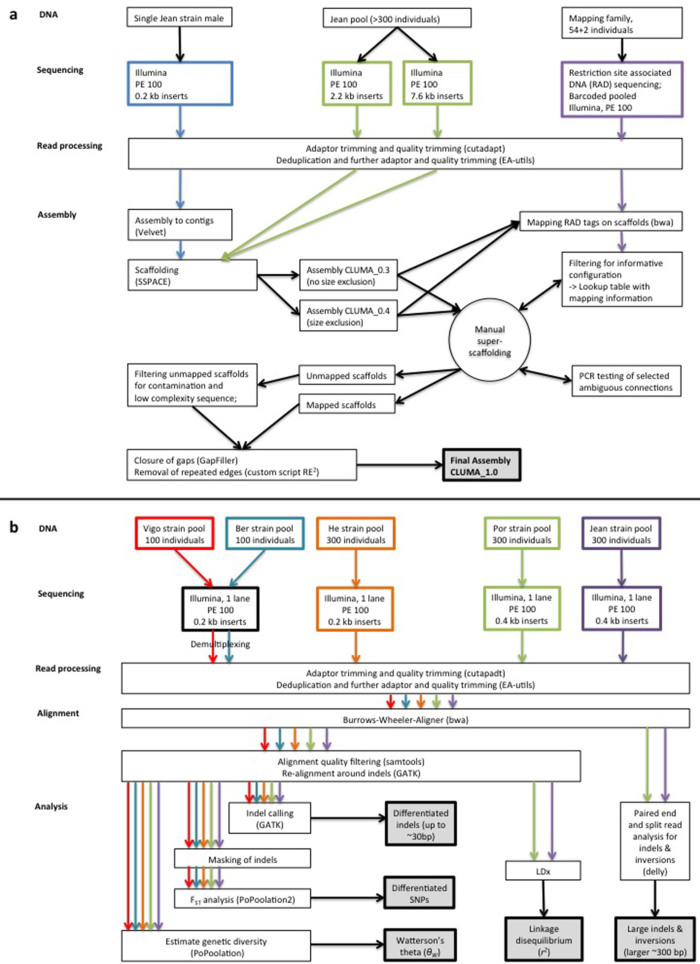
Analyses overview. **a**, Overview of the genome assembly process. **b**, Overview of the population genomic analyses.

The genome assembly process (Extended Data Fig. 9a) was based on three sequencing libraries (Supplementary Table 10): a 0.2-kb insert library was prepared from a single adult male of the Jean laboratory strain (established from field samples taken at St. Jean-de-Luz, France, in 2007; >12 generations in the laboratory), which was starved and kept in seawater with penicillin (60 units per ml), streptomycin (60 μg ml^−1^) and neomycin (120 μg ml^−1^) during the last 2 weeks of development. DNA was extracted with a salting-out method^46^, sheared on a Covaris S2 sonicator (frequency sweeping mode; 4 °C; duty cycle, 10%; intensity, 7; cycles per burst, 300; microTUBE AFA fibre 6 × 16 mm; 30 s) and prepared for Illumina sequencing with standard protocols. A 2.2-kb and a 7.6-kb insert library were prepared from a polymorphic DNA pool of >300 field-caught Jean adult males by Eurofins MWG Operon (Ebersberg, Germany) according to the manufacturer’s protocol. Each library was sequenced in one lane of an Illumina HiSeq2000 with 100-bp paired-end reads at the Next Generation Sequencing unit of the Vienna Biocenter Core Facilities (http://vbcf.ac.at).

Reads were filtered for read quality, adaptor and spacer sequences with cutadapt^47^ (−b −n 3 −e 0.1 −O 8 −q 20 −m 13) and duplicates were removed with fastq-mcf from ea-utils^48^ (−D 70). Read pairs were interleaved with ngm-utils^49^, leaving only paired reads. Contamination with human DNA found in the 0.2-kb library was removed by deleting reads matching the human genome at a phred-scaled quality score ≥ 20 (alignment with BWA^50^).

Assembly into contigs with Velvet^51^ (scaffolding disabled; 57-bp kmers as determined by VelvetOptimiser^52^) was based solely on the less polymorphic 0.2-kb library. About 600 remaining adaptor sequences at the ends of assembled contigs were trimmed with cutadapt (−O 8 −e 0.1 −n 3). For assembly statistics see Supplementary Table 11.

Scaffolding of the contigs was based on all three libraries and performed with SSPACE^53^ in two iterations, that is, scaffolds from the first round were scaffolded again. Using different parameters in the iterations (Supplementary Table 12) allowed different connections to be made and thus increased scaffold connectivity (Supplementary Table 13). The effect is probably owing to the polymorphic nature of the 2.2-kb and 7.6-kb libraries; it results in a ‘population-consensus most common arrangement of the scaffolds’. The iterative scaffolding process was performed with and without applying a size cut-off excluding contigs <1 kb, resulting in two independent assemblies (CLUMA_0.3 and CLUMA_0.4; see Extended Data Fig. 9a), which differed in overall connectivity and sequence content (Supplementary Table 11), but also in the identity and structure of the large scaffolds. In order to combine both connectivity and sequence content, and in order to resolve the contradictions in the structure of the largest scaffolds, the two assemblies were compared and reconciled in a manual super-scaffolding process, as detailed in Supplementary Method 1. Briefly, the overlap of scaffolds from the two assemblies was tested with BLAST searches and represented in a graphical network structure. Scaffolds with congruent sequence content in both assemblies would result in a linear network, whereas scaffolds with contradictory sequence content would result in branching networks. At the same time, both assemblies were subject to genetic linkage mapping based on genotypes obtained from restriction-site-associated DNA sequencing (RAD sequencing) of a published mapping family^6^ (Supplementary Method 2). The resulting genetic linkage information served to resolve the branching networks into the longest possible unambiguous linear sub-networks with consistent genetic linkage information (see scheme A in Supplementary Method 1). Finally, the structure of the resulting super-scaffolds was coded in YAML format and translated into DNA sequence with Scaffolder^54^, resulting in 75 mapped super-scaffolds.

**Extended Data Figure 10:**
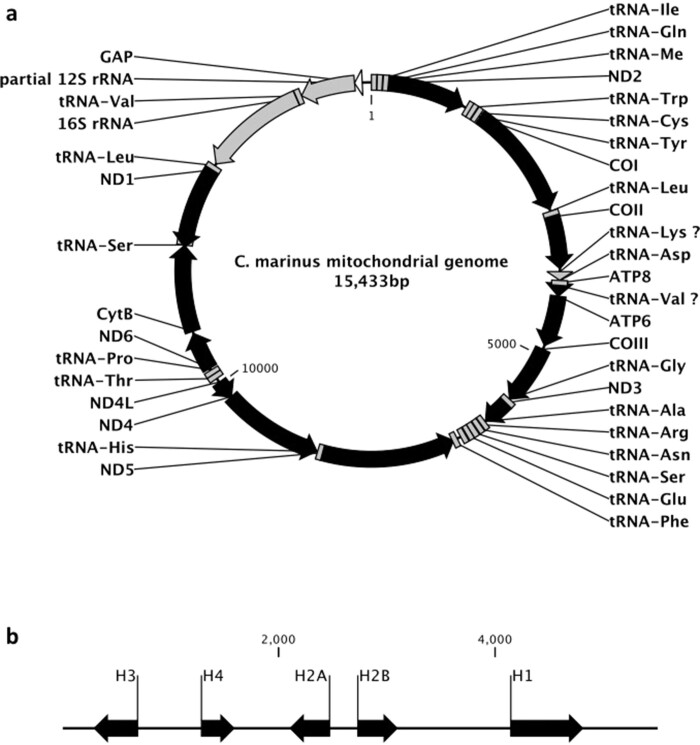
Arrangement of the mitochondrial genome and of the histone gene cluster in *C. marinus*. **a**, **b**, mitochondrial genome (**a**) and histone gene cluster (**b**) arrangements in *C. marinus*. Protein-coding genes are shown in black, tRNAs and rRNAs in grey.

The remaining small and unmapped scaffolds were filtered for fragments of the mitochondrial genome, the histone gene cluster and 18S/28S ribosomal rDNA gene cluster, which were assembled separately (Supplementary Method 3; Extended Data Fig. 10). Unmapped scaffolds were also filtered for obvious contamination from other species (Supplementary Method 3). The degree to which the remaining unmapped scaffolds are leftover polymorphic variants of parts of the mapped super-scaffolds was estimated by blasting the former against the latter (Supplementary Method 3 and Supplementary Table 14).

All scaffolds were subject to gap closing with GapFiller^55^ and repeated edges, that is, gaps with almost identical sequences at both sides that are generally not closed because of genetic polymorphisms, were assessed and if possible removed with a custom script (Supplementary Method 4; code available supplied as Source Data File).

The final assembly CLUMA_1.0 was submitted under project PRJEB8339 (75 mapped scaffolds; 23,687 unmapped scaffolds ≥100 bp). The assembly and further information can also be obtained from *ClunioBase* (http://cluniobase.cibiv.univie.ac.at).

### Reconstruction of chromosomes and QTL analysis

Genetic linkage information for the final 75 super-scaffolds was obtained by repeating read mapping to genotype calling for the RAD sequencing experiment as described above (Supplementary Method 2), but now with assembly CLUMA_1.0 as a reference. This allowed us to place and orient super-scaffolds along the genetic linkage map (Fig. 1a and Extended Data Fig. 2). The positions of the recombination events within a scaffold were approximated as the middle between the positions of the two RAD markers between which the marker pattern changed from one map location to the next. The published genetic linkage map was refined and revised (Supplementary Method 5 and Extended Data Fig. 2). Based on the refined linkage map, QTL analysis of the published mapping family was repeated as described^6^ (Supplementary Table 4 and Supplementary Note 5). Using the correspondence between the reference assembly and the genetic linkage map, we were able to directly identify the genomic regions corresponding to the confidence intervals of the QTLs (Fig. 1 and Extended Data Fig. 5a, b).

### Transcript sequencing

Assembled transcripts of a normalized cDNA library of all life stages and various *C. marinus* strains (454 sequencing) were available from previous experiments and RNA sequencing data was available for Jean strain adults (Illumina sequencing). Furthermore, specifically for genome annotation, RNA from 80 third instar larvae from the Jean and Por laboratory strains each was prepared for RNA sequencing according to standard protocols (Supplementary Method 6). Each sample was sequenced on a single lane of an Illumina HiSeq 2000. All transcript reads were submitted to the European Nucleotide Archive (ENA) under project PRJEB8339.

For the adult and larval RNA sequencing data, raw reads were quality checked with fastqc^56^, trimmed for adaptors quality with cutadapt^47^ and filtered to contain only read pairs using the interleave command in ngm-utils^49^. Reads were assembled separately for larvae and adults with Trinity^57^ (path_reinforcement_distance: 25; maximum paired-end insert size: 1,500 bp; otherwise default parameters).

### Genome annotation

Automated annotation was performed with MAKER2^58^. Repeats were masked based on all available databases in repeatmasker. MAKER2 combined evidence from assembled transcripts (see above), mapped protein data sets from *Culex quinquefasciatus* (CpipJ1), *Anopheles gambiae* (AgamP3), *Drosophila melanogaster* (BDGP5), *Danaus plexippus* (DanPle_1.0), *Apis mellifera* (Amel4.0), *Tribolium castaneum* (Tcas3), *Strigamia maritima* (Smar1) and *Daphnia pulex* (Dappu1) and *ab initio* gene predictions with AUGUSTUS^59^ and SNAP^60^ into gene models. AUGUSTUS was trained for *C. marinus* based on assembled transcripts from the normalized cDNA library. SNAP was run with parameters for *A. mellifera*, which had the highest congruence with known *C. marinus* genes in preliminary trials (Supplementary Method 7). MAKER was set to infer gene models from all evidence combined (not transcripts only) and gene predictions without transcript evidence were allowed. Splice variant detection was enabled, single-exon genes had to be larger than 250 bp and intron size was limited to a maximum of 10 kb.

All gene models within the QTL confidence intervals, as well as all putative circadian clock genes and light receptor genes were manually curated: exon–intron boundaries were corrected according to transcript evidence (approximately 500 gene models), chimeric gene models were separated into the underlying individual genes (approximately 100 gene models separated into around 300 gene models) and erroneously split gene models were joined (approximately 15 gene models). Finally, this resulted in 21,672 gene models, which were given IDs from CLUMA_CG000001 to CLUMA_CG021672 (‘CLUMA’ for *Clunio marinus*, following the controlled vocabulary of species from the UniProt Knowledgebase; CG for ‘computated gene’). Splice variants of the same gene (detected in 752 gene models) were identified by the suffix ‘-RA’, ‘-RB’ and so on, and the corresponding proteins by the suffix ‘-PA’, ‘-PB’ and so forth.

Gene models were considered as supported if they overlapped with mapped transcripts or protein data (Supplementary Table 1). Gene counts for *D. melanogaster* were retrieved from BDGP5, version 75.546 and for *A. gambiae* from AgamP3, version 75.3. The putative identities of the *C. marinus* gene models were determined in reciprocal BLAST searches, first against UniProtKB/Swiss-Prot (8,379 gene models assigned) and if no hit was found, second against the non-redundant protein sequences (nr database) at NCBI (1,802 additional genes assigned). Reciprocal best hits with an *e* value < 1 × 10^−10^ were considered putative orthologues (termed ‘putative gene X’), non-reciprocal hits with the same *e* value were considered paralogues (termed ‘similar to’). All remaining gene models were searched against the PFAM database of protein domains (111 gene models assigned; termed ‘gene containing domain X’). If still no hit was found, the gene models were left unassigned (‘NA’).

### Synteny comparisons

Genome-wide synteny between the *C. marinus*, *D. melanogaster* and *A. gambiae* genomes was assessed based on reciprocal best BLAST hits (*e* value < 10 × 10^-10^) between the three protein data sets (Ensembl Genomes, Release 22, for *D. melanogaster* and *A. gambiae*). Positions of pairwise orthologous genes were retrieved from the reference genomes (BDGP5, AgamP3 and CLUMA_1.0) and plotted with Circos^61^. *C. marinus* chromosome arms were delimited based on centromeric and telomeric signatures in genetic diversity and linkage disequilibrium (Extended Data Fig. 3c and Supplementary Table 3; for data source see ‘strain re-sequencing’ below). Homologues for *C. marinus* chromosome arms were assigned based on enrichment with putative orthologous genes from specific chromosome arms in *D. melanogaster* and *A. gambiae* (Extended Data Figs 3, 4 and Supplementary Table 3). Additionally, for the 5,388 detected putative 1:1:1 orthologues (*C. marinus*:*D. melanogaster*:*A. gambiae*), microsynteny was assessed by testing if all pairs of directly adjacent genes in one species were also directly adjacent in the other species. The degree of microsynteny was then calculated as the fraction of conserved adjacencies among all pairs of adjacent genes. From this fraction the relative levels of chromosomal rearrangements in the evolutionary lineage leading to *C. marinus* were estimated (Supplementary Note 3 and Extended Data Fig. 4).

### Strain re-sequencing

Genetic variation in five *C. marinus* strains (Extended Data Fig. 1) was assessed based on pooled-sequencing data from field-caught males from the strains of St. Jean-de-Luz (Jean; Basque Coast, France; sampled in 2007; *n* = 300), Port-en-Bessin (Por; Normandie, France; 2007; *n* = 300), as well as Vigo (Spain; 2005; *n* = 100), Helgoland (He; Germany; 2005; *n* = 300) and Bergen (Ber; Norway; 2005; *n* = 100). Samples from Vigo and Bergen, were provided by D. Neumann and C. Augustin, respectively. For each strain we chose the largest available number of individuals to obtain the best possible resolution of allele frequencies. Females are not available, because they are virtually invisible in the field. For an overview of the experimental procedure, see Extended Data Fig. 9b. DNA was extracted with a salting-out method^46^ from sub-pools of 50 males, the DNA pools were mixed at equal DNA amounts, sheared and prepared as described above and sequenced on four lanes of an Illumina HiSeq2000 with paired-end 100-bp reads (Ber and Vigo combined in one lane, distinguished by index reads). All reads were submitted to the European Nucleotide Archive (ENA) under project PRJEB8339. Sequencing reads were filtered for read quality and adaptor sequences with cutadapt^47^ (−b −n 2 −e 0.1 −O 8 −q 13 −m 15), interleaved with ngm-utils^49^ and duplicates were removed with fastq-mcf from ea-utils^48^ (−D 70). Reads were aligned to the mapped super-scaffolds of assembly CLUMA_1.0 with BWA^50^ (aln and sampe; maximal insert size (bp): −a 1500).

### Detection of re-arrangements

Based on the unfiltered alignments, the samples from Por and Jean were screened for genomic inversions and indels relative to the reference sequence with the multi-sample version of DELLY^62^. Paired-end information was only considered if the mapping quality was high (*q* ≥ 20) (see also Supplementary Note 3).

### Population genomic analysis of the timing strains

For population genomic analysis (Extended Data Fig. 9b), the alignments of the pool-sequencing (pool–seq) data from Vigo, Jean, Por, He and Ber were filtered for mapping quality (*q* ≥ 20), sorted, merged and indexed with SAMtools^63^. Reads were re-aligned around indels with the RealignerTargetCreator and the IndelRealigner in GATK^64^. The resulting coverage per strain is given in Supplementary Table 5.

For identification of SNPs, a pileup file was created with the mpileup command of SAMtools^63^. Base Alignment Quality computation was disabled (−B); instead, after creating a synchronized file with the mpileup2sync script in PoPoolation2^65^, indels that occurred more than ten times were masked (including 3 bp upstream and downstream) with the identify-indel-regions and filter-sync-by-gtf scripts of PoPoolations2. *F*_ST_ values were determined with the fst-sliding script of PoPoolation2, applying a minimum allele count of 10 (so that any false-positive SNPs resulting from the remaining unmasked indels were effectively excluded) and a minimum coverage of 40× for the comparison between Por and Jean or 10× for the comparison of all five strains. *F*_ST_ was calculated at a single base resolution, as well as in windows of 5 kb (step size, 1 kb). Individual SNPs were only considered for further analyses or plotted if they were significantly differentiated as assessed by Fisher’s exact test (fisher-test in PoPoolation2).

Average genome-wide genetic differentiation between timing strains, as obtained by averaging over 5-kb sliding-windows, was compared to the respective timing differences and geographic distances (see Supplementary Table 8) in Mantel tests (Pearson’s product moment correlation; 9,999 permutations), as implemented in the vegan package in the R statistical programming environment (ref. 66). Geographic distances and circadian timing differences were determined as described previously^67^ (see Supplementary Table 8). For determination of lunar timing differences when comparing lunar with semilunar rhythms see Supplementary Note 6. In order to find genomic regions for which genetic differentiation is correlated with the timing differences between strains, the Mantel test was then applied to 5-kb genomic windows every 1 kb along the reference sequence. 5 kb is roughly the average size of a gene locus in *C. marinus*. Windows with a correlation coefficient of *r* ≥ 0.5 were tested for significance (999 permutations). For each genomic position the number of overlapping significantly correlated 5-kb windows was enumerated, resulting in a correlation score (CS; ranging from 0 to 5).

Genetic diversity, measured as Watterson’s theta (*θ*_W_), for each strain was assessed with PoPoolation1.1.2 (ref. 68) in 20-kb windows with 10-kb steps. In order to save computing time, the pileup files of Jean, Por and He were linearly downscaled to 100× coverage with the subsample-pileup script (‘fraction’ option), positions below 100× coverage were discarded. Indel regions were excluded (default in PoPoolation 1.1.2) and a minimum of 66% of a sliding window needed to be covered. SNPs were only considered in *θ*_W_ calculations if present ≥2 times, leading to slight inconsistencies in *θ*_W_ estimates between strains due to differing coverage, but not affecting diversity comparisons within strains.

Linkage disequilibrium between the SNPs was determined for the Por and Jean strains with LDx^69^, assuming physical linkage between alleles on the same read or read pairs. *r*^2^ was determined by a maximum likelihood estimator, minimum and maximum read depths corresponded to the 2.5% and 97.5% coverage depths for each population (Jean, 111–315; Por, 98–319), total insert distance was limited to 600 bp, minimum phred-scaled base quality was 20, minimum allele frequency was 0.1 and a minimum coverage per pair of SNPs was 11. SNPs were binned by their physical distance for the plots (0–200 bp, 200–400 bp, 400–600 bp), with the mean value plotted.

Finally, small indels (<30 bp) in the Por and Jean strains were detected with the UnifiedGenotyper (−glm INDEL) in GATK^64^ for positions with more than 20× coverage. Genetic differentiation for indels was calculated with the classical formula *F*_ST_ = (*H*_T_−*H*_S_)/*H*_T_, where *H*_S_ is the average expected heterozygosity according to Hardy–Weinberg Equilibrium (HWE) in the two subpopulations and *H*_T_ is the expected heterozygosity in HWE of the hypothetical combined total population. If more than two alleles were present, only the two most abundant alleles were considered in the calculation of *F*_ST_.

### Assessment of candidate genes

Gene models from the automated annotation were considered candidate genes, if they fulfilled the following criteria. (1) The gene was located within the reference sequence corresponding to the QTL confidence intervals as determined for the Por and Jean strains. (2) The gene contained a strongly differentiated SNP or small indel or it was directly adjacent to such a SNP or small indel (*F*_ST_ ≥ 0.8 for Por versus Jean, that is, the strains used in QTL mapping). This resulted in a preliminary list of 133 genes based on the comparison between Por and Jean (Supplementary Table 6). These candidate genes were narrowed down based on their overlap with genomic 5-kb windows, for which genetic differentiation between five European timing strains correlated with their timing differences (Fig. 1a, Extended Data Fig. 5a, b and Supplementary Table 9).

The location and putative effects of the SNPs and indels relative to the gene models were assessed with SNPeff^70^ (−ud 0, otherwise default parameters; Extended Data Fig. 5c, d and Supplementary Tables 6, 9).

For Gene Ontology (GO) term analysis, all *C. marinus* gene models with putative orthologues in the UniProtKB/Swiss-Prot and non-redundant protein sequences (nr) databases based on reciprocal best BLAST hits (see above) were annotated with the GO terms of their detected orthologues (6,837 gene models). Paralogues were not annotated. The enrichment of candidate SNPs and indels (*F*_ST_ ≥ 0.8 between Por and Jean) in specific GO terms was tested with SNP2GO^71^ (min.regions = 1, otherwise default parameters). Hyper-geometric sampling was applied to test if individual genes of a GO term or a whole pathway of genes are enriched for SNPs (Supplementary Table 7).

### Molecular characterization of CaMKII.1

RNA-seq data of the Por and Jean strains for *CaMKII.1* were obtained from the larval RNA sequencing experiment described above. Besides four assembled full-length transcripts (RA–RD) from RNA-seq and assembled EST libraries, additional partial transcripts (RE–RO) were identified by PCR amplification (for PCR primers see Supplementary Table 15), gel extraction (QIAquick Gel Extraction Kit, Qiagen), cloning with the CloneJET PCR Cloning Kit (Thermo Scientific) and Sanger sequencing with pJET1.2 primers (LGC Genomics & Microsynth). cDNA was prepared from RNA extracted from third instar larvae of the Por and Jean laboratory strains (RNA extraction with RNeasy Plus Mini Kit, Qiagen; reverse transcription with QuantiTect Reverse Transcription Kit, Qiagen).

qPCR was performed with variant-specific primers and actin was used as a control gene (Supplementary Table 16). cDNA was obtained from independent pools of 20 third instar larvae of the Por and Jean strains. Sample size was ten pools per strain to cover different time points during the day and to test for reproducibility (two samples each at zeitgeber times 0, 4, 8, 16 and 20; for one Por sample extraction failed; RNA extraction and reverse transcription as above). qPCR was performed with Power SYBR Green PCR Master Mix on a StepOnePlus Real Time System (both Applied Biosystems). Fold-changes were calculated according to ref. 72 in a custom excel sheet. The assumption of equal variance was violated for the RD comparison (*F*-test) and the assumption of normal distribution was violated for the data of RA and RC in the Por strain (Shapiro–Wilk normality test), possibly reflecting circadian effects in the samples from different times of day. Thus, expression differences were assessed for significance in a two-tailed Wilcoxon rank-sum test (wilcox.test in R^66^). Holm correction^73^ was used for multiple testing (default in p.adjust function of R).

### CaMKII.1 minigenes

PCR fragments containing the CaMKII.1 linker region (exons 10–15) were amplified from genomic Por or Jean DNA, respectively, with primers CaMKII-Sc61-F-344112 and CaMKII-Sc61-R-351298 (Supplementary Table 15), cloned with the CloneJET PCR Cloning Kit (Thermo Scientific), transferred into the pcDNA3.1+ vector using NotI and XbaI (Thermo Scientific). These constructs were transfected into *D. melanogaster* S2R+ cells and RNA was prepared 48 h after transfection. After DNase digestion, isoform expression was analysed by radioactive, splicing-sensitive RT–PCR (primers in Supplementary Table 17) and phosphorimager quantification as described^74^. Identity of isoforms is based on size and sequencing of PCR products. To test for reproducibility, there were seven biological replicates (raw data in Supplementary Table 18). As the assumptions of equal variance (*F*-test) and normal distribution of data (Shapiro–Wilk normality test) were not violated, the significance of expression differences was assessed in unpaired, two-sided two-sample *t*-tests. Holm correction^73^ was used for multiple testing (default in p.adjust function of R). S2R+ cells were obtained from the laboratory of S. Sigrist, regularly authenticated by morphology and routinely tested for absence of mycoplasma contamination. The entire experiment was reproduced several months later with three biological replicates (raw data in Supplementary Table 18).

### S2 cell luciferase assay

Firefly luciferase is driven from a *period* 3X69 promoter under control of the CLOCK and CYCLE protein^19,21^. The *D. melanogaster pAc–clk* construct was obtained from F. Rouyer, *pCopia–Renilla luciferase* and *period 3X69–luc* reporter constructs from M. Rosbash, a [Ca^2+^]-independent mouse *CaMKII*^T286D^ was provided by M. Mayford. The CaMKII inhibitor KN-93 was purchased from Abcam (#ab120980).

*C. marinus Cyc*, *C. marinus Clk* and *C. marinus CaMKII.1–RD* were cloned into the pAc5.1/V5–His A plasmid (Invitrogen) with stop codons before the tag. The Q5 Site-Directed Mutagenesis Kit (NEB) was used to make kinase-dead and [Ca^2+^]-independent versions of *C. marinus CaMKII.1–RD* (for primers, see Supplementary Table 17).

*D. melanogaster* S2 cells (Invitrogen) were cultured at 25 °C in Schneider’s *D. melanogaster* medium (Lonza) supplemented with fetal bovine serum (FBS, 10%, heat-inactivated), penicillin (100 U ml^−1^), streptomycin (100 μg ml^−1^) and 2 mM l-glutamine; Sigma). Cells were seeded into 24-well plates (800,000 cells per well) and transfected with Effectene transfection reagent (Qiagen) according to the manufacturer’s instructions. Experiment with mouse [Ca^2+^]-independent CaMKII: 25 ng *pCopia–Renilla*, 10 ng *period 3X69–luc*, 0.5 ng *D. melanogaster pAc–clk*, 200 ng mouse *pAc–CaMKII*^T286D^. Experiment with CaMKII inhibitor KN-93: 25 ng *pCopia–Renilla*, 10 ng *period 3X69–luc*, 0.5 ng *D. melanogaster pAc–clk*, various amounts of KN-93. Experiment with *C. marinus* genes: 25 ng *pCopia–Renilla*, 10 ng *period 3X69–luc*, 100 ng *C. marinus pAc–cyc*, 100 ng *C. marinus pAc–clk*, 200 ng *C. marinus CaMKII.1–RD*^K42R^ or 200 ng *C. marinus CaMKII.1–RD*^T286D^. In all experiments, the transfection mix was filled up with empty pAc5.1/V5–His A vector to a total of 435 ng DNA per well. After 48 h, cells were washed with PBS and lysed with Passive Lysis Buffer (Promega). Luciferase activities were determined on a Synergy H1 plate reader (Biotek) using a Dual-Luciferase Reporter Assay System (Promega). For each biological replicate three independent cell lysates were measured and their mean value determined. Firefly luciferase activity was normalized to Renilla luciferase activity and values were normalized to controls transfected with *D. melanogaster pAc–clk* or *C. marinus pAc–clk* and *C. marinus pAc–cyc*, respectively. S2 cells (Invitrogen/Life Technologies, Cat.no. R690-07) were regularly authenticated by morphology and routinely tested for absence of mycoplasma contamination (Lonza MycoAlert). Sample size was chosen to test for reproducibility.

### Circadian free-run experiments

For circadian free-run experiments, culture boxes of the Por, He and Jean strains were transferred from light–dark cycle (16:8) to constant dim light (light–light cycle, about 100 lx). Emerging adults were collected in 1-h intervals by a custom made *C. marinus* fraction collector (similar to those described in ref. 75) and counted once a day. Because collection was automated, the experimenter had no influence on the results and blinding was not necessary. As the circalunar clock restricts adult emergence to a few days, the circadian emergence rhythm can only be assessed over a few days. Several culture boxes were transferred to a light–light cycle at different time points. The resulting emergence data were combined for each strain using the switch to a light–light cycle as a common reference point. We used the maximum number of available individuals. Free-running period was calculated as the mean interval between subsequent emergence peaks, weighting each peak by the number of individuals.

### Data availability

All sequence data are deposited in the European Nucleotide Archive (ENA) under PRJEB8339. The reference genome is also on *ClunioBase* (http://cluniobase.cibiv.univie.ac.at). Machine readable super-scaffolding data and the computer source code for the removal of repeated edges are supplied as source data files.

## Supplementary information

Supplementary Information This file contains Supplementary Tables 1-19, Supplementary Methods, Supplementary Notes, Supplementary References and a Supplementary Figure – see contents page for details. (PDF 2893 kb)

Supplementary Data This zipped file contains .yaml files containing all changes made to the genome assembly during the super-scaffolding process. (ZIP 20 kb)

Supplementary data This zipped file contains the source code for the software "Repeated Edge Remover (RE^2^)". (ZIP 241 kb)

## PowerPoint slides

PowerPoint slide for Fig. 1
PowerPoint slide for Fig. 2
PowerPoint slide for Fig. 3
