# Supplementary material for: The genomic basis of circadian and circalunar timing adaptations in a midge
Source: Nature. 2016 Nov 21;540(7631):69–73. doi: 10.1038/nature20151 (PMC5133387; doi:10.1038/nature20151)

doi:10.1038/nature20151

Table of Contents

Supplementary Tables 2
Table S1 Gene models with transcript and/or protein evidence 2
Table S2 The *C. marinus* mitochondrial ribosomal proteins 3
Table S3 *C. marinus* chromosome arms, corresponding reference scaffolds and homologous chromosome arms in *D. melanogaster* and *A. gambiae* 4
Table S4 QTL analysis on the original and revised genetic linkage maps 4
Table S5 Average coverage and covered positions (%) in strain resequencing 4
Table S6 Computed list of candidate genes for the *Por* vs *Jean* comparison 5
Table S7 Candidate-SNP-enriched GO terms for “Molecular function” in QTL C2 9
Table S8 Genetic divergence, timing differences and geographic distances for the five tested *C. marinus* strains 10
Table S9 Refined List of candidate genes for circadian and circalunar timing alterations 11
Table S10 Sequencing data in this study 12
Table S11 Statistics for different steps of the reference genome assembly process 13
Table S12 Scaffolding parameters for SSPACE 14
Table S13 Effect of scaffolding parameters and iterative scaffolding 14
Table S14 Putative fraction of polymorphic variants in the set of unmapped scaffolds 14
Table S15 PCR primers for detecting CaMKII.1 splice variants 15
Table S16 Primers for splice-variant specific quantitative real-time PCR 15
Table S17 Primers for S2 cell assays 16
Table S18 Raw data of relative quantification in slicing assay in S2R+ cells 17
Table S19 Manual edits to the assembly based on Sanger sequencing 18

Supplementary Methods 19
1 Super-scaffolding and PCR testing 19
2 Genetic linkage mapping 24
3 Assessing the unmapped scaffolds: Contamination, mitochondrial genome, gene clusters, polymorphic variants 26
4 Removal of repeated edges, gap closing 30
5 Revision of the genetic linkage map 34
6 Larval RNA sequencing 37
7 Gene prediction with SNAP 38

Supplementary Notes 39
1 Completeness of the reference genome and estimated gene numbers 39
2 Genome evolution in dipterans 41
3 An elevated rate of chromosomal rearrangements in the lineage leading to *C. marinus* 43
4 Refined QTL analysis for circadian and lunar timing 48
5 Differentiated SNPs in in the *C. marinus* timing strains 49
6 Determining lunar peak phase for semi-lunar rhythms 50

Supplementary References 52
Supplementary Figure 55

doi:10.1038/nature20151

Supplementary Tables

Table S1 Gene models with transcript and/or protein evidence

Annotation type	All gene models	Transcript support		Protein support		Transcript AND protein support		Transcript OR protein support	
	n	n	%	n	%	n	%	n	%
Combined evidence used in MAKER	7,103	6,574	92.6	5,993	84.4	5,701	80.3	6,866	96.7
AUGUSTUS prediction	3,646	3,084	84.6	2,917	80.0	2,580	70.8	3,421	93.8
SNAP prediction	10,390	2,935	28.3	920	8.9	631	6.1	3,224	31
Curated annotations	533	507	95.1	466	87.4	443	83.1	530	99.4
Sum	21,672	13,100	60.4	10,296	47.5	9335	43.2	14,041	64.8

Table S2 The *C. marinus* mitochondrial ribosomal proteins

mRp	*D. melanogaster* ID	*C. marinus* ID	*C. marinus* scaffold
mRpL1	gi|17737843|ref|NP_524275.1|	CLUMA_CG005923	31
mRpL2	gi|17647679|ref|NP_524022.1|	CLUMA_CG018042 2)	57A
mRpL3	gi|24642280|ref|NP_511166.2|	CLUMA_CG020684	7
mRpL4	gi|17933744|ref|NP_524939.1|	CLUMA_CG021264	9
mRpL9	gi|28571728|ref|NP_524363.3|	CLUMA_CG002113	15
mRpL10	gi|17647667|ref|NP_523440.1|	CLUMA_CG007521	41B
mRpL11	gi|17737961|ref|NP_524351.1|	CLUMA_CG008234	43
mRpL12	gi|17864338|ref|NP_524742.1|	CLUMA_CG000067	10
mRpL13	gi|24585068|ref|NP_523598.2|	CLUMA_CG014062 2)	48
mRpL14	gi|17986013|ref|NP_525048.1|	CLUMA_CG006577	40
mRpL15	gi|17737689|ref|NP_524185.1|	CLUMA_CG005458	27
mRpL16	gi|18079268|ref|NP_525041.1|	CLUMA_CG004164 2)	25
mRpL17	gi|24654665|ref|NP_523870.2|	CLUMA_CG005457	27
mRpL18	gi|20129927|ref|NP_610818.1|	CLUMA_CG014113	48
mRpL19	gi|17737855|ref|NP_524284.1|	CLUMA_CG012078	47D
mRpL20	gi|17647671|ref|NP_524051.1|	CLUMA_CG020398	61
mRpL21	gi|24666641|ref|NP_649095.2|	CLUMA_CG013628	48
mRpL22	gi|24642645|ref|NP_523379.2|	CLUMA_CG000196 2) 3)	10
mRpL23	gi|17647675|ref|NP_523889.1|	CLUMA_CG011977	47D
mRpL24	gi|17647677|ref|NP_523476.1|	CLUMA_CG005580	27
mRpL27	gi|28574706|ref|NP_787971.1|	CLUMA_CG008367	43
mRpL28	gi|20129239|ref|NP_608887.1|	CLUMA_CG013813	48
mRpL30	gi|17933570|ref|NP_525073.1|	CLUMA_CG005105	27
mRpL32	gi|17864144|ref|NP_524606.1|	CLUMA_CG003701	22A
mRpL33	gi|24639667|ref|NP_524981.2|	CLUMA_CG015230	50
mRpL34	gi|116007708|ref|NP_001036552.1|	CLUMA_CG011015	47C
mRpL35	gi|28571807|ref|NP_651001.2|	CLUMA_CG000845	12
mRpL36	gi|21357279|ref|NP_652658.1|	CLUMA_CG009823	45B
mRpL37	gi|281361540|ref|NP_524306.3| 1)	CLUMA_CG020397	61
mRpL38	gi|24641946|ref|NP_511152.2|	CLUMA_CG006531	40
mRpL39	gi|24664387|ref|NP_524075.2|	CLUMA_CG019062	59
mRpL40	gi|17737911|ref|NP_524318.1|	CLUMA_CG019971	60B
mRpL41	gi|21687222|ref|NP_611022.2|	CLUMA_CG017755	56
mRpL42	gi|17647695|ref|NP_523673.1|	CLUMA_CG021386	9
mRpL43	gi|17647665|ref|NP_523828.1|	CLUMA_CG005109	27
mRpL44	gi|21357105|ref|NP_649541.1|	CLUMA_CG012824	47G
mRpL45	gi|21355709|ref|NP_651072.1|	CLUMA_CG013102	47K
mRpL46	gi|21358503|ref|NP_647661.1|	CLUMA_CG013105 2)	47K
mRpL47 / rcl1	gi|28573151|ref|NP_788610.1|	CLUMA_CG018887	59
mRpL48	gi|19920526|ref|NP_608613.1|	CLUMA_CG010298	47A
mRpL49	gi|20129021|ref|NP_572839.1|	CLUMA_CG011035	47C
mRpL50	gi|21357011|ref|NP_648092.1|	CLUMA_CG019036	59
mRpL51	gi|19920958|ref|NP_609239.1|	CLUMA_CG019426	5
mRpL52	gi|20129763|ref|NP_610313.1|	CLUMA_CG012962	47H
mRpL53	gi|24653506|ref|NP_725343.1|	CLUMA_CG021246	9
mRpL54	gi|20130195|ref|NP_611541.1|	CLUMA_CG000133	10
mRpL55	gi|21356717|ref|NP_650780.1|	CLUMA_CG001722	15
mRpS2	gi|28574694|ref|NP_523473.2|	CLUMA_CG017279 2) 3)	54
mRpS5	gi|116007908|ref|NP_001036652.1| 1)	CLUMA_CG005908	31
mRpS6	gi|17986127|ref|NP_523925.1|	CLUMA_CG011922	47D
mRpS7	gi|17647699|ref|NP_523537.1|	CLUMA_CG007921 2) 3)	41B
mRpS9	gi|24644917|ref|NP_524270.2|	CLUMA_CG011885 2)	47C
mRpS10	gi|28571716|ref|NP_731985.2|	CLUMA_CG015746	51
mRpS11	gi|17738009|ref|NP_524382.1|	CLUMA_CG001939	15
mRpS12 / tko	gi|17933526|ref|NP_525050.1|	CLUMA_CG010667	47C
mRpS14	gi|24643241|ref|NP_728245.1|	CLUMA_CG005286	27
mRpS15 / bonsai	gi|19922752|ref|NP_611691.1|	CLUMA_CG021342	9
mRpS16	gi|17647683|ref|NP_523737.1|	CLUMA_CG009715 2)	45B
mRpS17	gi|24762582|ref|NP_525119.1|	CLUMA_CG016273	52
mRpS18A	gi|24645087|ref|NP_731252.1|	CLUMA_CG013095 2)	47K
mRpS18B	gi|24585392|ref|NP_724248.1|	CLUMA_CG000226 2)	10
mRpS18C	gi|24651373|ref|NP_524593.1|	CLUMA_CG021223	9
mRpS21	gi|24646553|ref|NP_731803.1|	CLUMA_CG007322	41B
mRpS22	gi|17738257|ref|NP_524537.1|	CLUMA_CG008994 2)	45A
mRpS23	gi|24584213|ref|NP_723847.1|	CLUMA_CG006535	40
mRpS24	gi|17986187|ref|NP_524476.1|	CLUMA_CG008692	44
mRpS25	gi|17986025|ref|NP_511153.1|	CLUMA_CG014351	49
mRpS26	gi|17647687|ref|NP_524134.1|	CLUMA_CG015061	50
mRpS28	gi|28573726|ref|NP_523785.2|	CLUMA_CG016491 2)	52
mRpS29	gi|17647691|ref|NP_523811.1|	CLUMA_CG017183	54
mRpS30	gi|17530957|ref|NP_511167.1|	CLUMA_CG011186	47C
mRpS31	gi|17977676|ref|NP_524100.1|	CLUMA_CG001835	15
mRpS33	gi|17738005|ref|NP_524380.1|	CLUMA_CG011878 2)	47C
mRpS34	gi|24665233|ref|NP_524104.2|	CLUMA_CG009323	45B
mRpS35	gi|17647689|ref|NP_523893.1|	CLUMA_CG006311	3

1) The original NCBI entry referred to in Marygold et al. 2007 has meanwhile been replaced by this new entry.
2) Chimeric gene model; contains additional gene(s).
3) The MRP is wrongly considered as UTR, i.e. it is not present in the predicted protein.

Table S3 *C. marinus* chromosome arms, corresponding reference scaffolds and homologous chromosome arms in *D. melanogaster* and *A. gambiae*

C. marinus chromosome arm	*C. marinus* reference scaffolds	*D. melanogaster* chromosome arm	*A. gambiae* chromosome arm	Muller element
1	60B, 60A, 12, 8, 39, 32, 29, 25, 59, 19, 27, 6, 22A, 22B, 23, 28, 58, 53, 15, 18, 14, 31, 43, 7, 17, 16A, 37, 38, 42, 57A, 16B, 44, 1, 11, 61	3R	2R	E
2L	20, 13, 46, 30, 54, 56, 62, 21, 26, 4, 40	X	X	A
2R	2, 50, 34, 35, 36, 47H, 47K, 55, 33, 47A, 47B, 47C, 47D, 47E, 47G, 47F	?	?	?
3L	10, 49, 41A, 41B, 48	2L	3R	B
3R	51, 52, 24, 45B, 45A, 5, 9, 3	3L	2L	D

Table S4 QTL analysis on the original and revised genetic linkage maps

	Kaiser & Heckel 2012[1]			Revised estimates			
	Location	R2	Additive effect	Location	R2	Additive effect	Size (Mb)
**Circadian QTL C1**	1-M5	0.29	1.17 h	1-M6	0.14	0.96 h	5.04
**Circadian QTL C2**	1-M16	0.12	0.75 h	1-M16 or 1-M17	0.13	0.93 h	3.26
**Circalunar QTL L1**	1-M4	0.23	3.2 d	1-M6	0.21	3.2 d	4.46
**Circalunar QTL L2**	2-M10	0.14	2.5 d	2-M10	0.13	2.5 d	1.17

h = hours d = days

Table S5 Average coverage and covered positions (%) in strain resequencing

**Strain**	**Jean**	**Por**	**He**	**Vigo**	**Ber**
**Sampled chromosomes (n)**	600	600	600	200	200
**Average Coverage**	243x	251x	177x	68x	101x
**% of positions with coverage > 100x**	97.4	97.0	95.0	2.7	58.3
**% of positions with coverage > 50x**	98.3	97.9	97.0	86.8	94.9
**% of positions with coverage > 20x**	98.9	98.6	98.0	97.1	97.5

doi:10.1038/nature20151

Table S6 Computed list of candidate genes for the *Por* vs *Jean* comparison

Gene ID	Putative gene	Scaffold	QTL	Correlation	SNPs							Indels						
					CDS: non-synonymous	CDS: synonymous	Splice site	5' UTR	3' UTR	Intron	Intergenic	Frameshift	Splice site	Stop gained	5' UTR	3' UTR	Intron	Intergenic
CLUMA_CG002902	NA	19	L1	L	.	.	.	.	.	.	1	.	.	.	.	.	.	1
CLUMA_CG002903	NA	19	L1	L	.	.	.	.	.	5	2	.	.	.	.	.	1	1
CLUMA_CG002904	NA	19	L1	L	.	.	.	.	.	.	1	.	.	.	.	.	.	1
CLUMA_CG002970	(sp) putative DNA fragmentation factor subunit alpha	19	L1	L	.	.	.	.	1	.	.	.	.	.	.	.	.	.
CLUMA_CG002971	NA	19	L1	L	1	.	.	.	.	.	.	.	.	.	.	.	.	.
CLUMA_CG002998	(sp) similar to Lachesin	19	L1	L	.	.	.	.	.	1	.	.	.	.	.	.	1	.
CLUMA_CG005125	(sp) similar to Insulin-like growth factor-binding protein complex acid labile subunit	27	L1	.	.	.	.	.	.	.	1	.	.	.	.	.	.	3
CLUMA_CG005126	(sp) similar to Tubulin polyglutamylase TTLL4	27	L1	. .	.	.	.	.	.	.	1	.	.	.	.	.	.	3
CLUMA_CG005135	(sp) similar to Translocator protein	27	L1	L	.	.	.	.	.	.	.	.	.	.	.	.	1	.
CLUMA_CG005214	(sp) putative Survival motor neuron protein	27	L1, C1	. .	.	.	.	.	.	.	2	.	.	.	.	.	.	.
CLUMA_CG005215	(nr) putative hypothetical protein AND_009556	27	L1, C1	. .	.	.	.	.	.	.	2	.	.	.	.	.	.	.
CLUMA_CG005305	(sp) putative Doublesex- and mab-3-related transcription factor A2	27	L1, C1	. .	.	.	.	.	.	.	.	.	.	.	.	.	.	1
CLUMA_CG005306	(sp) similar to General odorant-binding protein 99a	27	L1, C1	. .	.	.	.	.	.	.	.	.	.	.	.	.	.	1
CLUMA_CG005356	(sp) similar to Xanthine dehydrogenase	27	L1, C1	. .	.	.	.	.	.	.	.	.	.	.	.	.	.	1
CLUMA_CG005357	(sp) putative Dystonin	27	L1, C1	. .	.	.	.	.	.	.	.	.	.	.	.	.	.	1
CLUMA_CG005383	(sp) similar to TWiK family of potassium channels protein 18	27	L1, C1	. .	.	.	.	.	.	.	1	.	.	.	.	.	.	.
CLUMA_CG005385	NA	27	L1, C1	. .	.	.	.	.	.	.	1	.	.	.	.	.	.	.
CLUMA_CG005397	(sp) similar to Tubulin polyglutamylase ttll6	27	L1, C1	. .	.	.	.	1	.	.	.	.	.	.	.	.	.	.
CLUMA_CG005411	(sp) putative Synapsin	27	L1, C1	. .	.	.	.	.	.	.	.	.	.	.	.	.	1	.
CLUMA_CG005434	(nr) similar to AGAP006216-PB [Anopheles gambiae str. PEST]	27	L1, C1	. .	.	.	.	.	.	.	.	.	.	.	.	.	1	.
CLUMA_CG005447	(nr) similar to conserved hypothetical protein [Culex quinquefasciatus]	27	L1, C1	. .	1	.	.	.	.	.	.	.	.	.	.	.	.	.
CLUMA_CG005451	(sp) similar to Serine protease easter	27	L1, C1	L C	1	.	.	.	.	.	.	.	.	.	.	.	.	.
CLUMA_CG005525	(sp) putative Eukaryotic translation initiation factor 4E type 2	27	L1, C1	. .	.	.	.	.	.	.	.	.	.	.	.	.	.	1
CLUMA_CG005526	(sp) similar to Membrane-bound alkaline phosphatase	27	L1, C1	. .	.	.	.	.	.	.	.	.	.	.	.	.	.	1
CLUMA_CG005564	(sp) putative S-adenosylmethionine synthase	27	L1, C1	L C	.	.	.	.	.	.	.	.	.	.	.	.	1	.
CLUMA_CG005572	NA	27	L1, C1	. .	1	1	.	.	.	.	.	.	.	.	.	.	.	.
CLUMA_CG020438	(sp) putative DNA-directed RNA polymerase III subunit RPC8	6	L1, C1	. .	.	.	.	.	.	.	.	.	.	.	.	.	.	1
CLUMA_CG020439	(sp) putative Probable prefoldin subunit 6	6	L1, C1	. .	.	.	.	.	.	.	.	.	.	.	.	.	.	1
CLUMA_CG020460	NA	6	L1, C1	. .	.	.	.	.	.	.	.	.	.	.	.	.	.	1
CLUMA_CG020461	(sp) putative SprT-like domain-containing protein Spartan	6	L1, C1	. .	.	.	.	.	.	.	.	.	.	.	.	.	.	1
CLUMA_CG020468	(sp) similar to Membrane-associated protein Hem	6	L1, C1	. .	.	2	.	.	.	.	.	.	.	.	.	.	.	.
CLUMA_CG003668	(nr) similar to PREDICTED: uncharacterized protein LOC101740474 [Bombyx mori]	22A	L1, C1	. .	1	.	.	.	.	.	.	.	.	.	.	.	.	2
CLUMA_CG003669	(sp) similar to Protein ariadne-2	22A	L1, C1	. .	.	.	.	.	.	.	.	.	.	.	.	.	.	2
CLUMA_CG003769	(nr) putative hypothetical protein AaeL_AAEL004946 [Aedes aegypti]	22A	L1, C1	L .	.	.	.	.	.	.	.	.	.	.	1	.	.	.
CLUMA_CG003815	(sp) putative DmX-like protein 2	22B	L1, C1	. .	.	.	.	.	.	.	.	.	.	.	.	1	.	.
CLUMA_CG003925	(sp) similar to Chaoptin	23	L1, C1	. .	.	.	.	.	.	.	.	.	.	.	.	1	.	.
CLUMA_CG018806	NA	58	L1, C1	. .	.	.	.	.	.	.	4	.	.	.	.	.	.	2
CLUMA_CG018807	NA	58	L1, C1	. .	3	1	.	.	1	.	4	.	.	.	.	2	.	2
CLUMA_CG018841	(sp) putative Ras-related protein Rab-3	58	L1, C1	. .	.	.	.	.	.	.	2	.	.	.	.	.	.	.
CLUMA_CG018842	(sp) putative SH3 and cysteine-rich domain-containing protein 3	58	L1, C1	. .	.	.	.	.	.	.	2	.	.	.	.	.	.	.

Table S6 (continued)

Gene ID	Putative gene	Scaffold	QTL	Correlation	SNPs: CDS: non-synonymous	SNPs: CDS: synonymous	SNPs: Splice site	SNPs: 5' UTR	SNPs: 3' UTR	SNPs: Intron	SNPs: Intergenic	Indels: Frameshift	Indels: Splice site	Indels: Stop gained	Indels: 5' UTR	Indels: 3' UTR	Indels: Intron	Indels: Intergenic
CLUMA_CG003079	(sp) similar to Venom serine protease 34	1	C2	.	.	.	.	.	.	.	2	.	.	.	.	.	.	.
CLUMA_CG003080	NA	1	C2	.	.	2	.	.	.	.	2	.	.	.	.	.	.	.
CLUMA_CG003102	NA	1	C2	.	.	.	.	.	.	.	.	.	.	.	.	.	.	1
CLUMA_CG003103	(nr) putative hypothetical protein AaeL_AAEL005789 [Aedes aegypti]	1	C2	.	.	.	.	.	.	.	.	.	.	.	.	.	.	1
CLUMA_CG000458	(sp) similar to Chymotrypsin BI	11	C2	.	.	.	.	.	.	.	.	.	.	.	.	.	.	1
CLUMA_CG000459	NA	11	C2	.	.	.	.	.	.	.	.	.	.	.	.	.	.	1
CLUMA_CG000511	NA	11	C2	.	.	.	.	.	.	.	.	.	.	.	.	.	.	1
CLUMA_CG000512	NA	11	C2	.	.	.	.	.	.	.	.	.	.	.	.	.	.	1
CLUMA_CG000516	NA	11	C2	.	.	.	.	.	.	.	1	.	.	.	.	.	.	.
CLUMA_CG000517	NA	11	C2	.	.	.	.	.	.	.	1	.	.	.	.	.	.	.
CLUMA_CG000519	NA	11	C2	.	.	.	.	.	.	.	1	.	.	.	.	.	.	.
CLUMA_CG000520	NA	11	C2	.	.	.	.	.	.	.	1	.	.	.	.	.	.	.
CLUMA_CG000521	NA	11	C2	.	.	.	.	.	.	.	.	.	.	.	.	.	.	1
CLUMA_CG000522	NA	11	C2	C	.	.	.	.	.	1	.	.	.	.	.	.	1	1
CLUMA_CG000529	NA	11	C2	.	.	.	.	.	.	.	.	.	.	.	.	.	.	1
CLUMA_CG000530	NA	11	C2	.	.	.	.	.	.	.	1	.	.	.	.	.	.	1
CLUMA_CG000531	NA	11	C2	.	.	.	.	.	.	.	1	.	.	.	.	.	.	.
CLUMA_CG000538	(sp) putative L-ascorbate oxidase	11	C2	.	1	.	1	.	.	.	.	.	.	.	.	.	.	.
CLUMA_CG000587	(sp) similar to Zinc finger protein 836	11	C2	.	.	.	.	.	.	2	.	.	.	.	.	.	.	.
CLUMA_CG000589	(sp) putative RWD domain-containing protein 4	11	C2	.	.	.	.	2	.	.	.	.	.	.	.	.	.	.
CLUMA_CG000594	(sp) similar to Venom carboxylesterase-6 A	11	C2	C	.	.	.	.	.	.	.	.	.	.	.	.	.	1
CLUMA_CG000595	(sp) similar to Venom carboxylesterase-6 B	11	C2	.	.	1	.	.	.	.	.	.	.	.	.	.	.	1
CLUMA_CG000613	(sp) putative Low-density lipoprotein receptor-related protein 6	11	C2	C	.	.	.	.	.	.	.	.	.	.	.	.	.	1
CLUMA_CG000614	(sp) putative Probable U3 small nucleolar RNA-associated protein 11	11	C2	C	.	.	.	.	.	.	.	.	.	.	.	.	.	1
CLUMA_CG000621	NA	11	C2	C	.	.	.	.	.	.	1	.	.	.	.	.	.	.
CLUMA_CG000622	NA	11	C2	C	.	.	.	.	.	.	1	.	.	.	.	.	.	.
CLUMA_CG000679	(sp) similar to Elongation of very long chain fatty acids protein AAEL008004	11	C2	C	.	.	.	.	.	.	.	.	.	.	.	1	.	.
CLUMA_CG020082	(sp) putative Pre-rRNA-processing protein TSR1 homolog	61	C2	.	1	.	1	.	.	.	.	.	.	.	.	.	.	.
CLUMA_CG020094	(nr) similar to GSTD1-5 protein, putative [Pediculus humanus corporis]	61	C2	.	.	.	.	.	.	.	.	.	.	.	.	.	.	2
CLUMA_CG020095	(sp) putative Neurexin-3	61	C2	.	.	.	.	.	.	.	.	.	.	.	.	.	.	2
CLUMA_CG020099	(sp) putative Protein held out wings	61	C2	.	.	.	.	.	.	1	.	.	.	.	.	.	1	.
CLUMA_CG020100	NA	61	C2	.	.	1	.	.	.	.	.	.	.	.	.	.	.	.
CLUMA_CG020107	NA	61	C2	.	.	.	.	.	.	1	.	.	.	.	.	.	.	.
CLUMA_CG020117	(sp) putative RNA-binding protein squid	61	C2	C	.	.	.	.	.	1	.	.	.	.	.	.	.	.
CLUMA_CG020122	(sp) putative Probable pyruvate dehydrogenase E1 component subunit alpha, mitochondrial	61	C2	.	.	.	.	.	.	.	.	.	.	.	.	1	.	.
CLUMA_CG020133	NA	61	C2	C	.	.	.	.	.	.	1	.	.	.	.	.	.	.
CLUMA_CG020134	(nr) putative conserved hypothetical protein [Culex quinquefasciatus]	61	C2	C	.	.	.	.	.	.	1	.	.	.	.	.	.	.
CLUMA_CG020136	(sp) putative Protein tamozhennic	61	C2	C	.	.	.	.	.	.	1	.	.	.	.	.	.	.
CLUMA_CG020137	(sp) similar to Filamin-A / D. melanogaster: *jitterbug*	61	C2	C	.	1	.	.	.	.	1	.	.	.	.	.	2	.
CLUMA_CG020138	(sp) similar to Filamin-A / D. melanogaster: *cheerio*	61	C2	C	1	1	.	.	.	.	.	.	.	.	.	.	.	.
CLUMA_CG020150	(sp) putative Synaptosomal-associated protein 25	61	C2	.	.	.	.	.	.	1	.	.	.	.	.	.	2	.
CLUMA_CG020153	(sp) putative Sterol O-acyltransferase 1	61	C2	C	.	.	.	.	.	.	1	.	.	.	.	.	.	.
CLUMA_CG020154	(sp) similar to Serine protease 42	61	C2	C	.	.	.	.	.	.	1	.	.	.	.	.	.	.
CLUMA_CG020157	(sp) putative Protein groucho	61	C2	C	.	.	.	.	.	1	.	.	.	.	.	1	.	.
CLUMA_CG020162	(sp) putative Dedicator of cytokinesis protein 6	61	C2	C	.	1	.	.	.	.	.	.	.	.	.	.	.	.

doi:10.1038/nature20151

Table S6 (continued)

Gene ID	Putative gene	Scaffold	QTL	Correlation	SNPs: CDS: non-synonymous	SNPs: CDS: synonymous	SNPs: Splice site	SNPs: 5' UTR	SNPs: 3' UTR	SNPs: Intron	SNPs: Intergenic	Indels: Frameshift	Indels: Splice site	Indels: Stop gained	Indels: 5' UTR	Indels: 3' UTR	Indels: Intron	Indels: Intergenic
CLUMA_CG020163	(sp) putative Vacuolar protein sorting-associated protein 26	61	C2	C	.	.	.	.	1	.	1	.	.	.	.	.	.	1
CLUMA_CG020164	(sp) putative Calcium/calmodulin-dependent protein kinase type II alpha chain	61	C2	C	2	1	.	.	.	20	1	.	.	.	.	.	5	1
CLUMA_CG020165	(sp) putative Protein fem-1 homolog CG6966	61	C2	.	.	.	.	.	1	1	5	.	.	.	.	1	.	1
CLUMA_CG020166	NA	61	C2	.	1	.	.	.	.	.	6	.	.	.	.	.	.	4
CLUMA_CG020167	(sp) putative Y+L amino acid transporter 2	61	C2	C	.	.	.	2	.	12	1	.	.	.	1	.	5	3
CLUMA_CG020169	(nr) putative cuticular protein glycine-rich 13 precursor [Bombyx mori]	61	C2	C	.	.	.	.	.	6	.	.	1	.	.	.	3	.
CLUMA_CG020170	(sp) putative Angio-associated migratory cell protein	61	C2	C	.	1	.	1	.	.	1	.	.	.	.	.	1	2
CLUMA_CG020171	(sp) putative Pro-interleukin-16 / D. melanogaster: *big bang*	61	C2	C	7	2	.	1	.	.	1	.	.	.	.	.	1	2
CLUMA_CG020172	(sp) putative Kinesin-like protein unc-104	61	C2	C	.	.	.	.	.	5	.	.	.	.	.	2	1	.
CLUMA_CG020173	NA	61	C2	C	.	.	.	.	.	1	.	.	.	.	.	.	.	.
CLUMA_CG020174	(sp) similar to Venom dipeptidyl peptidase 4	61	C2	C	1	.	.	.	.	1	.	.	.	.	.	.	.	.
CLUMA_CG020179	(sp) putative Tubulin alpha-3 chain	61	C2	C	.	.	.	.	.	.	.	.	.	.	.	.	.	1
CLUMA_CG020180	NA	61	C2	C	.	.	.	.	.	.	.	.	.	.	.	.	.	1
CLUMA_CG020181	NA	61	C2	C	.	.	.	.	.	.	.	.	.	.	.	.	1	.
CLUMA_CG020195	(sp) putative Sodium/potassium-transporting ATPase subunit alpha	61	C2	C	.	.	.	1	.	1	.	.	.	.	.	.	.	.
CLUMA_CG020207	(sp) putative Probable tyrosyl-DNA phosphodiesterase	61	C2	.	.	.	.	.	.	.	1	.	.	.	.	.	.	.
CLUMA_CG020208	(sp) similar to Netrin receptor DCC	61	C2	.	.	.	.	.	.	.	1	.	.	.	.	.	4	.
CLUMA_CG020213	(sp) putative Peripheral plasma membrane protein CASK	61	C2	.	.	.	.	.	.	4	.	.	.	.	.	.	5	.
CLUMA_CG020254	(sp) putative Mitochondrial import inner membrane translocase subunit Tim22	61	C2	.	.	.	.	.	.	.	.	.	.	.	1	.	.	.
CLUMA_CG020270	(sp) putative Odorant receptor 56a	61	C2	.	.	.	.	.	.	.	.	.	.	.	.	.	1	.
CLUMA_CG020290	(sp) putative UDP-N-acetylhexosamine pyrophosphorylase	61	C2	.	1	.	.	.	.	.	.	.	.	.	.	1	.	.
CLUMA_CG020298	(sp) putative Vacuolar protein sorting-associated protein 13D	61	C2	.	.	1	.	.	.	.	.	.	.	.	.	.	.	.
CLUMA_CG020303	(sp) putative Vesicle transport protein USE1	61	C2	.	.	.	.	.	.	.	.	.	.	.	.	.	.	1
CLUMA_CG020304	(sp) putative Helicase domino	61	C2	.	.	.	.	.	.	.	.	.	.	.	.	.	.	1
CLUMA_CG020358	(sp) putative Ras-related protein Rab6	61	C2	C	.	.	.	1	.	.	.	.	.	.	.	.	.	.
CLUMA_CG020378	(sp) putative Helicase ARIP4	61	C2	.	.	1	.	.	.	.	.	.	.	.	.	.	.	.
CLUMA_CG020388	(nr) putative conserved hypothetical protein [Culex quinquefasciatus]	61	C2	.	.	1	.	.	.	.	.	.	.	.	.	.	1	.
CLUMA_CG020394	(sp) similar to Sodium-dependent nutrient amino acid transporter 1	61	C2	.	.	.	.	.	.	.	1	.	.	.	.	.	.	.
CLUMA_CG020395	(nr) putative fau [Drosophila yakuba]	61	C2	C	.	.	.	.	.	.	1	.	.	.	.	.	1	.
CLUMA_CG020403	(sp) putative KAT8 regulatory NSL complex subunit 1	61	C2	.	.	.	.	.	.	1	.	.	.	.	.	.	.	.
CLUMA_CG020404	(nr) similar to PREDICTED: similar to myoblast city CG10379-PA [Tribolium castaneum]	61	C2	.	1	.	.	.	.	2	.	.	.	.	.	.	1	.
CLUMA_CG017399	NA	56	L2	.	.	.	.	.	.	.	2	.	.	.	.	.	.	2
CLUMA_CG017400	(sp) putative Transient-receptor-potential-like protein	56	L2	.	.	.	.	.	.	.	2	.	.	.	.	.	.	2
CLUMA_CG017426	NA	56	L2	.	.	.	.	.	.	.	1	.	.	.	.	.	.	.
CLUMA_CG017427	(nr) similar to hypothetical protein AaeL_AAEL015357 [Aedes aegypti]	56	L2	.	.	.	.	.	.	.	1	.	.	.	.	.	.	.

Table S6 (continued)

Gene ID	Putative gene	Scaffold	QTL	Correlation	SNPs							Indels						
					CDS: non-synonymous	CDS: synonymous	Splice site	5' UTR	3' UTR	Intron	Intergenic	Frameshift	Splice site	Stop gained	5' UTR	3' UTR	Intron	Intergenic
CLUMA_CG017434	(sp) putative Cyclin-dependent kinase 5 activator 1	56	L2	L	.	.	.	.	.	.	.	.	.	.	.	.	.	1
CLUMA_CG017435	NA	56	L2	L	.	.	.	.	.	.	.	1	.	1	.	.	.	2
CLUMA_CG017436	(sp) putative Serine/threonine-protein kinase PLK4	56	L2	L	1	.	.	.	.	.	.	.	.	.	.	1	.	3
CLUMA_CG017437	NA	56	L2	L	.	.	.	.	.	.	.	.	.	.	.	.	.	2
CLUMA_CG017449	NA	56	L2	L	.	.	.	.	.	.	3	.	.	.	.	.	.	4
CLUMA_CG017450	(sp) putative Peroxisome biogenesis factor 10	56	L2	.	.	.	.	.	.	.	3	.	.	.	.	.	.	4
CLUMA_CG017473	(sp) similar to Zinc finger and SCAN domain-containing protein 10	56	L2	.	1	.	.	.	.	.	.	.	.	.	.	.	.	.
CLUMA_CG017474	(sp) putative Iron/zinc purple acid phosphatase-like protein	56	L2	.	.	.	.	.	1	.	.	.	.	.	.	.	.	.
CLUMA_CG017507	(sp) putative 3-phosphoinositide-dependent protein kinase 1	56	L2	.	.	.	.	.	.	.	.	.	.	.	.	.	.	1
CLUMA_CG017508	(nr) similar to hypothetical protein CAPTEDRAFT_186396 [Capitella teleta]	56	L2	.	.	.	.	.	.	.	.	.	.	.	.	.	.	1
CLUMA_CG017606	NA	56	L2	.	.	.	.	.	.	.	.	.	.	.	.	.	.	1
CLUMA_CG017607	(sp) putative Zinc finger protein squeeze	56	L2	L	.	.	.	.	.	.	.	.	.	.	.	.	.	1
CLUMA_CG017653	(sp) putative Cytochrome c oxidase assembly factor 5	56	L2	.	.	.	.	.	1	.	.	.	.	.	.	.	.	.

doi:10.1038/nature20151

Table S7 Candidate-SNP-enriched GO terms for “Molecular function” in QTL C2

GO	P	FDR	p.L	p.G	nc	GO term definition
GO:0005102	3,23E-05	0.0107	0	1	12	Interacting selectively and non-covalently with one or more specific sites on a receptor molecule, a macromolecule that undergoes combination with a hormone, neurotransmitter, drug or intracellular messenger to initiate a change in cell function.
GO:0005125	5,77E-21	1,62E-17	NA	NA	12	Functions to control the survival, growth, differentiation and effector function of tissues and cells.
GO:0005516	0.0001	0.0291	0.0891	0.9996	25	Interacting selectively and non-covalently with calmodulin, a calcium-binding protein with many roles, both in the calcium-bound and calcium-free states.
GO:0016301	0.0002	0.0435	0.0219	0.9996	25	Catalysis of the transfer of a phosphate group, usually from ATP, to a substrate molecule.
GO:0016773	0.0002	0.0435	0.0222	0.9996	25	Catalysis of the transfer of a phosphorus-containing group from one compound (donor) to an alcohol group (acceptor).
GO:0004672	0.0001	0.0274	0.0217	0.9996	25	Catalysis of the phosphorylation of an amino acid residue in a protein, usually according to the reaction: a protein + ATP = a phosphoprotein + ADP.
GO:0015297	4,65E-18	4,88E-15	NA	NA	14	Enables the active transport of a solute across a membrane by a mechanism whereby two or more species are transported in opposite directions in a tightly coupled process not directly linked to a form of energy other than chemiosmotic energy. The reaction is: solute A(out) + solute B(in) = solute A(in) + solute B(out).
GO:0008509	3,04E-08	1,97E-05	0.0008	1	14	Catalysis of the transfer of a negatively charged ion from one side of a membrane to the other.
GO:0015171	4,30E-06	0.0019	0.0040	1	14	Catalysis of the transfer of amino acids from one side of a membrane to the other. Amino acids are organic molecules that contain an amino group and a carboxyl group.

GO terms in child-parent relationships are in blocks of the same colour.
P: P-value of the Fisher's exact test.
FDR: Adjusted P-Value after applying the Benjamini-Hochberg method.
p.L: Proportion of iterations in which the hypergeometric sampling found less or equal candidate regions than observed. Low values indicate, that the SNPs cluster in fewer genes than expected if they were randomly distributed over the GO term.
p.G: Proportion of iterations in which the hypergeometric sampling found more or equal candidate regions than observed. High values indicate, that the SNPs are not overdispersed, i.e. not the whole GO term is enriched for candidate SNPs.
nc: Number of candidate SNPs

QTLs C1/L1 and L2 are not given, because in those QTLs no GO term was significantly enriched for candidate SNPs.

doi:10.1038/nature20151

Table S8 Genetic divergence, timing differences and geographic distances for the five tested *C. marinus* strains

**Genetic divergence (F_{ST})**	**Jean**	**Por**	**He**	**Ber**
**Vigo**	0.088	0.142	0.162	0.157
**Jean**	-	0.113	0.137	0.145
**Por**		-	0.084	0.119
**He**			-	0.120

**Circadian timing difference (hours)**	**Jean**	**Por**	**He**	**Ber**
**Vigo**	0.97	2.89	0.89	2.15
**Jean**	-	3.86	1.86	3.12
**Por**		-	2.00	0.74
**He**			-	1.26

**Circalunar timing difference (days)**	**Jean**	**Por**	**He**	**Ber**
**Vigo**	1.9	9.3	6.0	9.8
**Jean**	-	11.2	7.9	11.7
**Por**		-	3.3	0.5
**He**			-	3.8

**Geographic distance (km)**	**Jean**	**Por**	**He**	**Ber**
**Vigo**	685	1810	2620	3305
**Jean**	-	1125	1935	2620
**Por**		-	810	1495
**He**			-	685

Table S9 Refined List of candidate genes for circadian and circalunar timing alterations

Gene ID	Putative gene	QTL	SNPs (F_{ST} >= 0.8)						Indels (F_{ST} >= 0.8)						
			CDS: non-synonymous	CDS: synonymous	5' UTR	3' UTR	Intron	Intergenic	Frameshift	Splice site	Stop gained	5' UTR	3' UTR	Intron	Intergenic
CLUMA_CG002902	NA	L1	.	.	.	.	.	1	.	.	.	.	.	.	1
CLUMA_CG002903	NA	L1	.	.	.	.	5	2	.	.	.	.	.	1	1
CLUMA_CG002904	NA	L1	.	.	.	.	.	1	.	.	.	.	.	.	1
CLUMA_CG002970	putative DNA fragmentation factor subunit alpha	L1	.	.	.	1	.	.	.	.	.	.	.	.	.
CLUMA_CG002971	NA	L1	1	.	.	.	.	.	.	.	.	.	.	.	.
CLUMA_CG002998	similar to lachesin	L1	.	.	.	.	1	.	.	.	.	.	.	1	.
CLUMA_CG005135	similar to translocator protein	L1	.	.	.	.	.	.	.	.	.	.	.	1	.
CLUMA_CG005451	similar to serine protease easter	L1, C1	1	.	.	.	.	.	.	.	.	.	.	.	.
CLUMA_CG005564	putative S-adenosylmethionine synthase	L1, C1	.	.	.	.	.	.	.	.	.	.	.	1	.
CLUMA_CG003769	putative hypothetical protein AaeL_AAEL004946	L1, C1	.	.	.	.	.	.	.	.	.	1	.	.	.
CLUMA_CG000522	NA	C2	.	.	.	.	1	.	.	.	.	.	.	1	1
CLUMA_CG000594	similar to venom carboxylesterase-6 A	C2	.	.	.	.	.	.	.	.	.	.	.	.	1
CLUMA_CG000595	similar to venom carboxylesterase-6 B	C2	.	1	.	.	.	.	.	.	.	.	.	.	1
CLUMA_CG000613	putative low-density lipoprotein receptor-related protein 6	C2	.	.	.	.	.	.	.	.	.	.	.	.	1
CLUMA_CG000614	putative probable U3 small nucleolar RNA-associated protein 11	C2	.	.	.	.	.	.	.	.	.	.	.	.	1
CLUMA_CG000621	NA	C2	.	.	.	.	.	1	.	.	.	.	.	.	.
CLUMA_CG000622	NA	C2	.	.	.	.	.	1	.	.	.	.	.	.	.
CLUMA_CG000679	similar to elongation of very long chain fatty acids protein AAEL008004	C2	.	.	.	.	.	.	.	.	.	.	1	.	.
CLUMA_CG020117	putative RNA-binding protein squid	C2	.	.	.	.	1	.	.	.	.	.	.	.	.
CLUMA_CG020133	NA	C2	.	.	.	.	.	1	.	.	.	.	.	.	.
CLUMA_CG020134	putative conserved hypothetical protein	C2	.	.	.	.	.	1	.	.	.	.	.	.	.
CLUMA_CG020136	putative protein tamozhennic	C2	.	.	.	.	.	1	.	.	.	.	.	.	.
CLUMA_CG020137	putative filamin-A *jitterbug*	C2	.	1	.	.	.	1	.	.	.	.	.	2	.
CLUMA_CG020138	putative filamin-A *cheerio*	C2	1	1	.	.	.	.	.	.	.	.	.	.	.
CLUMA_CG020153	putative sterol O-acyltransferase 1	C2	.	.	.	.	.	1	.	.	.	.	.	.	.
CLUMA_CG020154	similar to serine protease 42	C2	.	.	.	.	.	1	.	.	.	.	.	.	.
CLUMA_CG020157	putative protein groucho	C2	.	.	.	.	1	.	.	.	.	.	1	.	.
CLUMA_CG020162	putative dedicator of cytokinesis protein 6	C2	.	1	.	.	.	.	.	.	.	.	.	.	.
CLUMA_CG020163	putative vacuolar protein sorting-associated protein 26	C2	.	.	.	1	.	1	.	.	.	.	.	.	1
CLUMA_CG020164	putative Ca2+/calmodulin-dependent protein kinase type II alpha chain	C2	2	1	.	.	20	1	.	.	.	.	.	5	1
CLUMA_CG020167	putative Y+L amino acid transporter 2	C2	.	.	2	.	12	1	.	.	.	1	.	5	3
CLUMA_CG020169	putative cuticular protein glycine-rich 13 precursor	C2	.	.	.	.	6	.	.	1	.	.	.	3	.
CLUMA_CG020170	putative angio-associated migratory cell protein	C2	.	1	1	.	.	1	.	.	.	.	.	1	2
CLUMA_CG020171	putative pro-interleukin 16 / *big bang*	C2	7	2	1	.	.	1	.	.	.	.	.	1	2
CLUMA_CG020172	putative kinesin-like protein unc-104	C2	.	.	.	.	5	.	.	.	.	.	2	1	.
CLUMA_CG020173	NA	C2	.	.	.	.	1	.	.	.	.	.	.	.	.
CLUMA_CG020174	similar to venom dipeptidyl peptidase 4	C2	1	.	.	.	1	.	.	.	.	.	.	.	.
CLUMA_CG020179	putative tubulin alpha-3 chain	C2	.	.	.	.	.	.	.	.	.	.	.	.	1
CLUMA_CG020180	NA	C2	.	.	.	.	.	.	.	.	.	.	.	.	1
CLUMA_CG020181	NA	C2	.	.	.	.	.	.	.	.	.	.	.	1	.
CLUMA_CG020195	putative sodium/potassium-transporting ATPase subunit alpha	C2	.	.	1	.	1	.	.	.	.	.	.	.	.
CLUMA_CG020358	putative ras-related protein Rab6	C2	.	.	1	.	.	.	.	.	.	.	.	.	.
CLUMA_CG020395	putative fau	C2	.	.	.	.	.	1	.	.	.	.	.	1	.
CLUMA_CG017434	putative cyclin-dependent kinase 5 activator 1	L2	.	.	.	.	.	.	.	.	.	.	.	.	1
CLUMA_CG017435	NA	L2	.	.	.	.	.	.	1	.	1	.	.	.	2
CLUMA_CG017436	putative serine/threonine-protein kinase PLK4	L2	1	.	.	.	.	.	.	.	.	.	1	.	3
CLUMA_CG017437	NA	L2	.	.	.	.	.	.	.	.	.	.	.	.	2
CLUMA_CG017449	NA	L2	.	.	.	.	.	3	.	.	.	.	.	.	4
CLUMA_CG017607	putative zinc finger protein squeeze	L2	.	.	.	.	.	.	.	.	.	.	.	.	1

NA = no homology identified based on reciprocal BLAST against the the UniProtKB/Swiss-Prot database, the nr database at NCBI or the PFAM database
Homologs are termed "putative ..." if reciprocal best blast hits suggest orthology.
Homologs are termed "similar to ...", if the reciprocal blast does not give the same hit, so that paralogy is suggested.

Table S10 Sequencing data in this study

Strain	Sample	Origin	Library	Read pairs	Raw data (Gbp)
**Reference genome assembly**					
Jean	1 male	Laboratory strain; partially reared on antibiotics	Paired-end; 0.2 kb inserts	75,010,280	15.0
Jean	> 300 males	field-caught	Paired-end; 2.2 kb inserts	167,846,208	33.6
Jean	> 300 males	field-caught	Paired-end; 7.6 kb inserts	121,877,597	24.4
**Restriction-site Associated DNA (RAD) sequencing for genetic mapping**					
NA	Mapping family; 2 parents, 54 progeny	Backcross of laboratory strains: Jean x (Jean x Por)	Paired-end; individuals barcoded	187,471,717	18.7
**Strain resequencing**					
Jean	300 males	field-caught	Paired-end; 0.4 kb inserts	192,528,404	38.5
Por	300 males	field-caught	Paired-end; 0.4 kb inserts	179,623,466	35.9
Vigo	100 males	field-caught	Paired-end; 0.2 kb inserts	46,638,962	9.3
Helgoland	300 males	field-caught	Paired-end; 0.2 kb inserts	136,199,228	27.2
Bergen	100 males	field-caught	Paired-end; 0.2 kb inserts	70,822,367	14.2
**RNA sequencing**					
Jean	80 larvae; stage LIII	Laboratory strain	Paired-end; 0.4 kb inserts	103,791,980	20.8
Por	80 larvae; stage LIII	Laboratory strain	Paired-end; 0.4 kb inserts	115,335,790	23.1

doi:10.1038/nature20151

Table S11 Statistics for different steps of the reference genome assembly process

Assembly	Contigs	CLUMA_0.3	CLUMA_0.4	CLUMA_0.5	CLUMA_1.0	CLUMA_1.0-M	CLUMA_1.0-U
**Characteristics**	Only contigs after assembly with *Velvet*	After scaffolding with *SSPACE*	After scaffolding with *SSPACE*; 1kb size cutoff	After super-scaffolding, PCR editing and filtering of unmapped scaffolds	After gap-filling and repeated edge removal	only mapped scaffolds	only unmapped scaffolds
**Total length (bp)**	83,680,134	93,902,885	82,804,957	91,460,826	85,566,647	78,546,749	7,019,898
**Gaps (n)**	NA	27,339	15,296	26,749	2,500	2,151	349
**Gaps (bp)**	NA	7,195,205	9,561,599	7,153,469	1,125,375	835,876	289,499
**Contigs (n)**	57,531	57,161	16,041	52,017	27,768	2,226	25,542
**Contig N50 (bp)**	5,472	5,387	6,641	5,520	79,428	87,461	292
**Contig N90 (bp)**	750	674	2,062	779	6,024	22,119	126
**Largest contig (bp)**	47,664	47,719	47,664	47,719	458,179	458,179	31,926
**Scaffolds (n)**	NA	29,822	745	25,268	25,268	75	25,193
**Scaffold N50 (bp)**	NA	819,709	1,106,940	1,997,709	1,871,155	1,896,271	317
**Scaffold N90 (bp)**	NA	44,575	310,679	252,023	162,901	498,469	128
**Largest scaffold (bp)**	NA	2,125,375	4,219,199	5,726,594	5,381,421	5,381,421	78,969
**AT content (%)**	67.90	67.93	68.08	68.23	68.19	68.28	67.18

doi:10.1038/nature20151

Table S12 Scaffolding parameters for SSPACE

Parameter	1st iteration	2nd iteration
Minimum number of links (k)	4	13
Maximal ratio of best connection to second-best connection (a)	0.3	0.5
Contig overlap in bp required for merging contigs (n)	15	15
Contig extension enabled (x)	1	0
Number of supporting reads needed to extend a contig (o)	20	NA
Required read overlap during extension in basepairs (m)	35	NA
Required base ratio to accept a overhanging consensus base (r)	0.9	NA
Basepairs to be trimmed if extension is not possible (t)	0	NA
Contig size cutoff (z)	0 / 1 [1)]	NA

Both parameter sets are stricter than SSPACE default parameters.
[1)] The iterative scaffolding procedure was performed once without size cutoff (leading to Assembly CLUMA_0.3), and once with size cutoff (leading to Assembly CLUMA_0.4). Compare Extended Data Fig. 9a and Table S11.

Table S13 Effect of scaffolding parameters and iterative scaffolding

Parameter set	Scaffold N50 (kb)	Largest scaffold (kb)
a=0.3, k=4	169	744
a=0.7, k=12	245	1497
a=0.3, k=4 followed by a=0.5, k=13	820	2125

a = maximum ratio of best to second best connections
k = minimum number of links
Only the two most extreme parameter sets tested for single scaffolding steps are presented, either being very strict on the requirement for the best connection, but not very strict on the required minimum number of links (a=0.3, k=4), or the other way around (a=0.7, k=12). The parameters applied in the iterative scaffolding are stricter than those of the extreme cases and stricter than SSPACE defaults.
A third iteration does increase the connectivity of the assembly notably.

Table S14 Putative fraction of polymorphic variants in the set of unmapped scaffolds

Parsing parameters		Unmapped scaffolds with hits in mapped scaffolds			
Min. Identity	Min. length of the hit[1]	Nr of scaffolds	% of scaffolds	bp	% of bp
0.98	0.9	11,385	45.2	2,099,216	29.9
0.95	0.9	16,973	67.4	3,255,821	46.4
0.9	0.9	18,534	73.6	3,604,868	51.4

[1] expressed as fraction of the contig length

doi:10.1038/nature20151

Table S15 PCR primers for detecting CaMKII.1 splice variants

Primer name	Sequence (5’ to 3’)
CaMKII-Sc61-F-341701-Start	ACGACTTTAGAAAAGAAACTTTAATCA
CaMKII-Sc61-F-344112	AAAAAGTGAAGGATCGCAAG
CaMKII-Sc61-F-347315	CAAACTTTCGCGGTACGAG
CaMKII-Sc61-R-345139	TTAGTGCAACTGAAAGGCTGAA
CaMKII-Sc61-R-347928	TCAACACTAAGAAGACTCCCAACA
CaMKII-Sc61-R-351298	CAACGACTCCGGTTCAAATG
CaMKII-Sc61-R-351793-Stop	TATAATCCTAGTTTCATTTGCTTCCT

Table S16 Primers for splice-variant specific quantitative real-time PCR

Primer name	Sequence (5’ to 3’)
CaMKII-RA-qF	CTGACTCAAGTACAACCATTGAAGA
CaMKII-RA-qR_te	CGTTGATTTGCCTTGACATT▴CTT
CaMKII-RB-qF	ACTGACTCAAGTACAACCATTGAA
CaMKII-RB-qR_te	AGGACAAACAATCCTTACAT▴CTTC
CaMKII-RC-qF_te	GAAGATGATGATGTGAAAG▴ATGT
CaMKII-RC-qR	TCATTTTGATGATTTCCTGACG
CaMKII-RD-qF	GAAGTTCAATGCGAGACGAA
CaMKII-RD-qR_te	TTCCTGACGCCGAG▴CTTT
CaMKII-RE-qF_te	CATAGCTAAAGATCCTGAAG▴GTG
CaMKII-RE-qR	ATTGCACTCGTTCCTGGAGT
CaMKII-RF-qF	TTTGAAGCACCCTTGGATCT
CaMKII-RF-qR_te	CGTAATCATACTTTTAC▴CTTTAG
CaMKII-RG-qF_te	GCGAGACGAAAACTAAAG▴GGTGC
CaMKII-RG-qR	GGTTCTCTTCGAAAATACTTAGCC
CaMKII-RH-qF_te	CTCAATCGGTCTTG▴GTCCA
CaMKII-RH-qR	TCCCAACAGACCCACTTTTC
CaMKII-RI-qF	TCAATTATTTCTCTACATAGGTCCA
CaMKII-RI-qR_te	GTCAAGCATATTGAGATTGT▴ATATTAG
CaMKII-RJ-qF	CTTTCATTTTCTGCTCTTTTCAA
CaMKII-RJ-qR_te	GACAAACAATCCTTACAT▴CCGA
CaMKII-RK-qF_te	GTTGAATCAATATTTTCGG▴ATGA
CaMKII-RK-qR	TGATGGCTTCGATAAGTTGTTC
CaMKII-RL-qF	TTTTCCCTTTCAACTTCTTTCAA
CaMKII-RL-qR_te	TTTCCTGACGCCGAG▴CCC
CaMKII-RM-qF	GAAGCAAAACTATTAACTGATAAACC
CaMKII-RM-qR	TCATTTTGATGATTTCCTGACG
CaMKII-RN-qF	AAAAAGTGAAGGATCGCAAGTT
CaMKII-RN-qR_te	AAGCATATTGAGATTGT▴CTTCAGG
CaMKII-RO-qF_te	TGAAGATGATGATGTGAAAG▴CAAG
CaMKII-RO-qR	CCCTTCTACTAAATTTCCCAACG
Act-qF	GCGGTATTCACGAGACAACAT
Act-qR	TCAGCGATTCCAGGATACATT

▴ Position at which the primer is crossing a variant specific splice site

Table S17 Primers for S2 cell assays

Primer name	Sequence (5’ to 3’)
RT PCR for minigenes	
BGH reverse	TAGAAGGCACAGTCGAGG
T7 forward primer	TAATACGACTCACTATAGGG
Primers for Q5 site-directed mutagenesis of CmCaMKII.1 (mutation underlined)	
CaMKII_K42R_mut_F	AGAATAATCAACACAAAAAAATTAACTTCC
CaMKII_K42R_mut_R	TGCAGCGAACTCCAAGCT
CaMKII-T286D_mut_F	GCAAGAAGACGTTGACTGTTTG
CaMKII-T286D_mut-R	CTATGAACAACTGACGCAACACG

Table S18 Raw data of relative quantification in slicing assay in S2R+ cells

Experiment 1 (shown in Fig. 3b)

	CaMKII.1 allele	Por				Jean			
	Splice Variant	RB	RC	RD	RO	RB	RC	RD	RO
Replicate	1	1,28	20,15	75,86	2,71	1,39	12,07	79,55	6,99
	2	2,32	31,04	66,11	0,53	1,63	20,09	74,12	4,16
	3	2,70	23,32	72,22	1,76	0,53	14,80	77,42	7,25
	4	1,34	23,62	72,15	2,89	1,10	15,47	78,18	5,25
	5	2,96	24,44	71,80	0,80	1,65	17,18	79,40	1,76
	6	2,34	20,79	76,87	0,00	1,29	13,98	81,22	3,51
	7	1,98	35,64	62,38	0,00	0,46	14,11	80,13	5,29

Experiment 2 (not shown)

	CaMKII.1 allele	Por				Jean			
	Splice Variant	RB	RC	RD	RO	RB	RC	RD	RO
Replicate	1	3,19	42,01	52,96	1,84	0,31	8,03	63,43	28,22
	2	5,88	27,57	66,55	0,00	2,22	18,46	68,52	10,80
	3	1,83	31,99	61,22	4,97	1,47	10,07	69,57	18,89

Table S19 Manual edits to the assembly based on Sanger sequencing

Super-scaffold	Position	Gene	Comments
7	866870 - 872231	*period*	sequence inserted; available unpubl. sequence
16A	38643	NA	sequence/gap removed based on PCR testing
16A	39056	NA	sequence/gap removed based on PCR testing
16B	4741	NA	sequence/gap removed based on PCR testing
16B	26734	NA	sequence/gap removed based on PCR testing
16B	50451 - 50534	NA	sequence inserted; generated during PCR testing
19	361434 - 363423	NA	sequence inserted; generated during PCR testing
22A	579413 - 580237	NA	sequence inserted; generated during PCR testing
25	184032 - 185169	NA	sequence inserted; generated during PCR testing
26	1406840	*cry1*	sequence/gap removed; available from [1]
27	400189	*cOps2*	sequence/gap removed; available from [1]
43	1081840	*cOps1*	sequence/gap removed; available from [1]
43	1082101..1082142	*cOps1*	sequence inserted; available from [1]
43	1083016	*cOps1*	sequence/gap removed; available from [1]
47C	640899	*rpS12*	sequence/gap removed; available from [1]
47C	2596356	*rOps2*	sequence/gap removed; available from [1]
58	661518 - 663177	NA	sequence inserted; generated during PCR testing
58	685728	NA	sequence/gap removed based on PCR testing
58	704553 - 705004	NA	sequence inserted; generated during PCR testing
58	711416	NA	sequence/gap removed based on PCR testing
59	684	NA	sequence/gap removed based on PCR testing
59	1397 - 1606	NA	sequence inserted; generated during PCR testing

doi:10.1038/nature20151

Supplementary Methods

1 Super-scaffolding and PCR testing

Comparison between assemblies CLUMA_0.3 and CLUMA_0.4 revealed that they were not fully identical in structure, leading to asymmetric overlaps of scaffolds but also to a number of ambiguities (compare scheme A below). We made use of the overlaps in a manual super-scaffolding approach, at the same time attempting to resolve the ambiguities based on genetic linkage information (see Methods and Supplementary Method 2) and testing of connections by polymerase chain reaction (PCR) and Sanger sequencing.

First, overlaps of scaffolds of the two different assemblies were assessed by searching the first and the last contig >1kb in a scaffold in the respective other assembly (the size requirement being due to the fact that assembly CLUMA_0.4 only contains contigs >1kb). Then the potentially overlapping regions of two scaffolds were blasted against each other and the results were visualized in a dot blot to assure that the overlapping regions are indeed close to identical in sequence and structure. The process of overlap detection was carried out until all scaffolds of assembly CLUMA_0.3, which had genetic linkage information and were larger than 10 kb, were assessed for their overlap with scaffolds in assembly CLUMA_0.4. The overlaps were represented in a graphic network structure (see scheme A, panel A), which highlighted conflicts such as inversions (Case I), ambiguities (Cases II and III) and scaffolding errors (Case IV).

In a second step, the network structure was resolved into individual super-scaffolds according to the following rules:

1) *Inversions*. Comparison of the two assemblies revealed a number of polymorphic inversions, usually at scaffold ends where they hindered further extension, having one arrangement in assembly CLUMA_0.3 and the other in assembly CLUMA_0.4 (see scheme A, panel A, Case I). For three cases we tested if these structures were truly inversions by PCR amplification over the inversion breakpoints (scheme B, panel A). In all three cases PCR on DNA from the *Jean* strain samples (300 individuals) confirmed that both arrangements exist in the strain, supporting the idea

of polymorphic inversions (scheme B, panel B). In the case of these inversions we arbitrarily decided for the arrangement found in assembly CLUMA_0.3 (see scheme A, panels A and B, Case I)

Scheme A

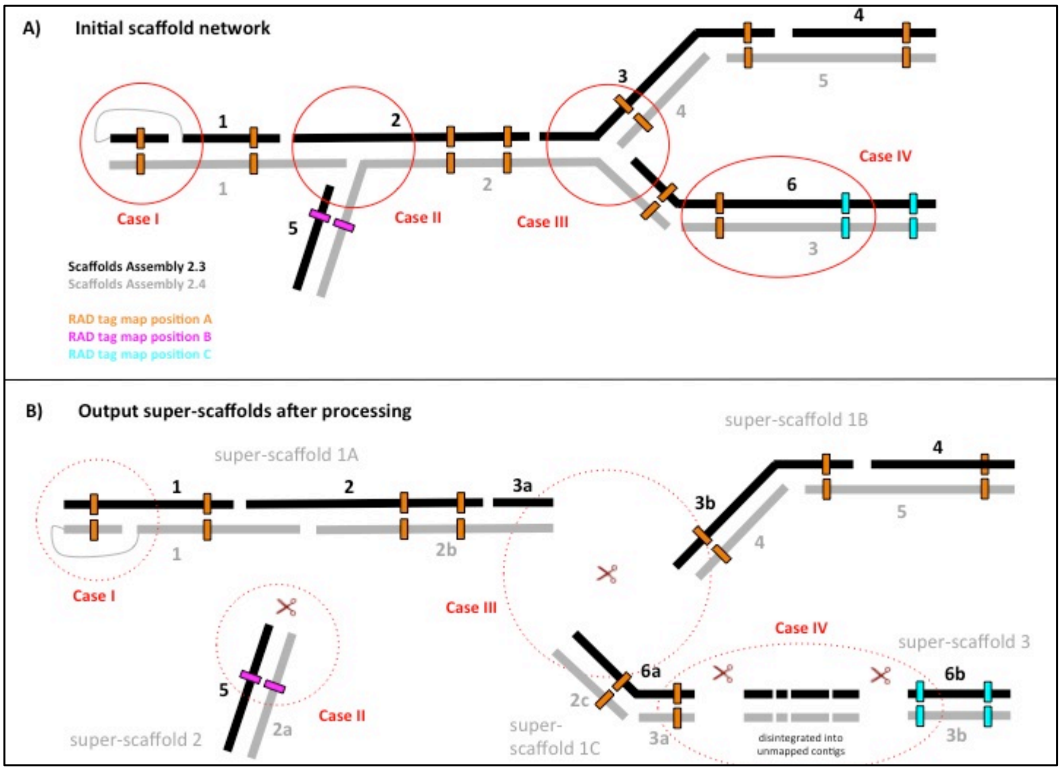

Scheme B

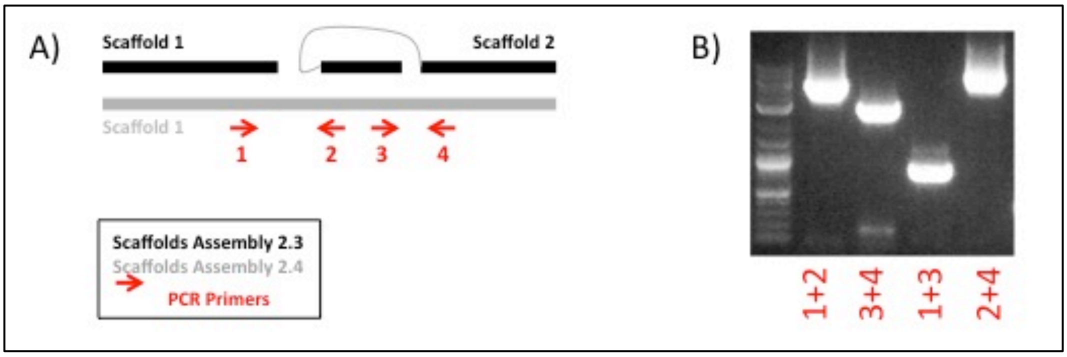

2) *Ambiguities*. Where the assemblies are contradictory in structure, the graphical representation results in a branching point (scheme A, panel A, Case II and III). For the three connected branches we first consulted the genetic linkage information (compare scheme A, coloured boxes on scaffolds).

2.1) If two branches had consistent map positions (according to genetic linkage information), but the third branch differed in map position (scheme A, Case II), we kept the connection of the first two branches and cut the third branch. The third branch would either be connected to other scaffolds and form a super-scaffold with those or it would be treated as an individual super-scaffold.

2.2) If all three branches had the same map position (scheme A, Case III), we checked if they are inside or outside our regions of interest, i.e. the quantitative trait loci (QTLs) identified in a previous study[1].

2.2.1) If the ambiguities were outside the QTLs, we separated all branches into individual super-scaffolds (scheme A, panel B, Case III).

2.2.2) If the ambiguities were inside the QTLs we designed PCR primers across the three possible connections in the ambiguity and tried to confirm connections by PCR. We accepted the amplified connection only if one connection was clearly amplified and the other two clearly not. However, this was only successful in a single case. In all other cases, several or even all possible connections were successfully amplified, suggesting that the ambiguities may be due to repetitive sequences or polymorphic genomic rearrangements (e.g. inversions that go beyond the scope of the scaffolds involved). These ambiguous connections were also broken into three individual super-scaffolds. The super-scaffolds resulting from breaking a single network with consistent map location were marked as potentially connected by naming them with the same number, but different letters as identifiers (e.g. scaffold 47A and scaffold 47B, etc.).

3) *Scaffolding errors*. In a small number of scaffolds, there were no ambiguities in the scaffold network, but genetic linkage information indicated that the scaffolds from both assembly CLUMA_0.3 and assembly CLUMA_0.4 were mis-scaffolded in the same way (scheme A, panel A, Case IV). These cases suffer from the problem that the position of the error cannot be inferred from the branching point of the network (as there is no branching point). In these cases we first checked if these scaffolding-errors are inside or outside the previously determined QTLs.
   3.1) If the scaffolding errors were outside the QTLs, we broke the scaffolds at the ends of the last contigs with known map location. The region in between was disintegrated into contigs and the contigs were treated as unmapped (scheme A, panel B, Case IV).
   3.2) If the scaffolding errors were inside the QTLs, we designed PCR primers in the middle between the two last contigs with known map location. We decided on four informative progeny from the mapping family based on the adjacent marker patterns, so that for two individuals the marker pattern would change across the wrong connection and for the other two it would not change across that connection. We then performed PCR on the four informative progeny and on the mapping parents, directly Sanger sequenced the PCR products and screened the chromatograms for informative polymorphisms. This allowed to decide on the map location of the amplified fragment. Then the process was repeated iteratively for the remaining unassigned sequence until the position of the scaffolding-error was pinned down to a defined gap between two contigs within the scaffold.

The resulting structure of the super-scaffolds was coded in YAML format. The code for all edited super-scaffolds is given in Supplementary File 1 to document the changes made to the assembly during super-scaffolding. Super-scaffolds not listed in Supplementary File 1 correspond to unedited scaffolds from assembly CLUMA_0.3. The software *Scaffolder*[2] was used to read the YAML code and output the .fasta files of the resulting super-scaffolds. In order to retain

the full sequence information, the sequence of the super-scaffolds was always compiled from the scaffolds in assembly CLUMA_0.3, which did not have a contig size cutoff during scaffolding.

Manual super-scaffolding resulted in 75 mapped super-scaffolds and 29,715 unmapped scaffolds. Notably, the manual super-scaffolding procedure does not insert any connections beyond those that were initially present in the automated scaffolding with SSPACE and which are supported by the respective quality criteria. Furthermore, all resulting super-scaffolds are supported by consistent genetic linkage information.

Finally, a number of available Sanger sequences were used in order to fill gaps or resolve small ambiguous regions (Table S19).

2 Genetic linkage mapping

A Restriction-site Associated DNA (RAD) sequencing library was prepared for the DNA of the same mapping family that was originally used to establish a genetic linkage map of the *C. marinus* genome[1]. The published RAD protocol[3] was slightly modified: For each of the 56 individuals (F1 parent, backcross parent and 54 progeny) 200ng of genomic DNA were digested with *BamHI* (NEB; 1h at 37°C). A combinatorial barcoding approach was used, i.e. both P1 and P2 adapters were barcoded. A set of 28 custom P1-adapters with 6bp-barcodes were ligated to the sticky ends and individuals were pooled into two groups, so that each barcode was unique in each of the two pools. Then DNA in both pools was sheared to an average fragment size of 700 bp on a *Covaris S2* sonicator (in frequency sweeping mode at 4°C; duty cycle: 10%; intensity: 7; cycles/burst: 300; in a TUBE AFA Fiber 12x12 mm). The pooled samples were concentrated by precipitation (0.2M NaCl and 0.8 vol. isopropanol; washing of the pellet with 70% ethanol), run out on a 1% agarose gel, size selected to 400-1000bp and extracted from the gel (ZymoClean Gel DNA Recovery Kit, Zymo Research). Then the sheared DNA ends were blunted (Quick Blunting™ Kit, NEB), an A overhang was added (Klenow Fragment (3’-->5’ exo-), NEB) and for each pool a P2 adaptor with a pool-specific 4-bp barcode was ligated to the A overhang. Both samples were subject to 18 cycles of PCR amplification (30s at 95°C; 18 cycles of 10s at 95°C, 30s at 65°C, 30s at 72°C; 5 min at 72°C) with the published P1 and P2 primers. The PCR products were size selected and cleaned up again. Both samples were run in a 1:1 ratio for 100bp paired-end reads in one lane of an Illumina HiSeq sequencer at the CSF Next Generation Sequencing facility of the Vienna Biocenter. All reads were submitted to the European Nucleotide Archive (ENA) under project PRJEB8339.

The reads were quality trimmed with cutadapt[4] (-q 20). For each individual reads were aligned to the reference scaffolds in assemblies CLUMA_0.3 and CLUMA_0.4 using the *aln* and *samse* commands of the Burrows-Wheeler-Aligner (BWA)[5] allowing a maximum of 4% divergence, producing 2 x 56 alignments. Alignments were filtered for mapping quality (>=20) and merged into one sorted and indexed alignment file for each assembly using the *view*, *sort*, *merge* and

index commands of SAMtools[6]. Then variants and genotypes were called using the UnifiedGenotyper implemented in the Genome Analysis Toolkit (GATK)[7]. The resulting genotypes were filtered with a custom script to have a minimum phred-scaled genotype quality (GQ) of 20; genotypes below the threshold were treated as "missing". Then the variable sites were parsed to have a minimum of 50 unambiguous genotypes (out of 56) and a minimum of 15 heterozygous genotype calls. The resulting genotypes were imported into an Excel spreadsheet and further filtered for male informative markers (that were heterozygous in the hybrid father of the mapping family and homozygous for the reference allele in the backcross mother, i.e. QTL informative) or for female informative markers (that were homozygous for the reference allele in the father and heterozygous in the mother). This resulted in 3,031 male informative markers and 1,850 female informative markers for assembly CLUMA_0.3 and 3,339 male informative markers and 2,569 female informative markers for assembly CLUMA_0.4 respectively. Two individuals had mostly missing or low quality genotypes, which slightly reduced the resolution of the map. All markers were inspected visually. Both male and female informative markers were assessed for their marker patterns along all scaffolds longer than 10 kb in order to determine the preliminary map location of these scaffolds.

doi:10.1038/nature20151

3 Assessing the unmapped scaffolds: Contamination, mitochondrial genome, gene clusters, polymorphic variants

The unmapped scaffolds and contigs were expected to contain four major types of sequences: (1) Sequence contamination from other organisms, (2) fragments of the mitochondrial genome and of gene clusters that are hard to assemble, (3) short polymorphic variants of parts of the nuclear genome that are already contained in the mapped scaffolds, and finally (4) truly unmapped and unique scaffolds of the *C. marinus* nuclear genome. The first three classes are problematic sequences and were dealt with in the following way:

(1) **Contamination**. All unmapped scaffolds were subject to a blastx search against the nr database at NCBI. Sequences > 2kb and sequences <= 2kb were treated independently.

Scaffolds larger 2kb were searched in pieces of 1kb. All blast results were loaded into the metagenomic analysis pipeline MEGAN4[8]. MEGAN4 was used to analyze all blast hits with a bit score larger 50. Based on the ten best blast hits, sequences were assigned to the following phylogenetic classes (number of assignments in brackets): *root* (12), *cellular organism* (27), *bacteria* (240), *eukaryota* (36), *ophistokonta* (3), *metazoa* (503), *not assigned* (254), *no hit* (582). For example, if the ten best blast hits of a sequence would only be metazoans, the sequence would be assigned to the class *metazoa*. A sequence that would hit both metazoans and bacteria, would be assigned to the class *root*. As scaffolds were blast searched in 1kb pieces, all scaffolds >2kb had a minimum of two blast hits, increasing confidence in the assignment of the complete scaffold. All scaffolds, which had hits in the class *bacteria*, contained only hits in the class *bacteria*. These scaffolds were removed from the assembly. There was a single exception, where one 1 kb fragment of a scaffold had a hit in *bacteria* (best blastx hit: *Wolbachia*) and another 1kb fragment had a hit in *metazoa* (all ten hits: insects). This suggests that *Wolbachia* sequences might be integrated in the *C. marinus* genome; this scaffold was not discarded. All other scaffolds were composed of a mixture of all classes but *bacteria*. All of these scaffolds contained hits in the class *metazoa*. The blast hits in the classes *root*, *cellular organism*, *eukaryota* or

ophistokonta contained either highly conserved genes or low complexity regions, and therefore gave no reason to discard the scaffolds. All of these scaffolds were kept.

Scaffolds < 2kb were searched as a whole in a blastx search against nr. Blast hits were also loaded into MEGAN4 and in a first round all hits with a bit-score larger 35 were assigned to the classes *root* (168), *viruses* (31), *archaea* (30), *bacteria* (3189), *cellular organism* (1169), *eukaryota* (6531), *not assigned* (564) and *no hit* (17503). They were treated in the following way:

- Hits in the class *root* were inspected individually. This class contained four cloning vector sequences (removed), 117 sequences of phage *phiX174*, which is spiked into the library during sequencing (removed), three *Wolbachia* sequences (kept) and 44 unclear hits (kept).
- Hits in the classes *viruses*, *archaea* and *bacteria* were removed.
- For the class *cellular organism* 50 hits (of 1169) were inspected individually. All of these could not be clearly assigned. Many of them hit both insects and bacteria, suggesting they may be sequences of bacteria commonly found in or on insects and that were in some insect genomes wrongly annotated as insect sequences. In order to be conservative, the sequences of the class *cellular organism* were not removed.
- Sequences in the class *eukaryota* were subject to further analysis (see below).
- Sequences in the classes *not assigned* and *no hit* were kept.

For sequences in the class *eukaryota* a new bit-score cutoff of 50 was applied and then they were assigned within *eukaryota* to the classes *eukaryota* (basically the "root" of *eukaryota*; 407), *alveolata* (105), *viridiplantae* (16), *ophistokonta* (50), *fungi* (116) and *metazoa* (3075). These hits were treated in the following way:

- Hits in the class *eukaryota* were inspected individually. There were 275 low complexity sequences (removed), two minisatellite sequences (removed), 129 sequences of highly conserved genes, e.g. ubiquitin, actin and histones (kept) and a single sequence likely representing the alveolate *Perkinsus marinus* (removed).

- Hits in the class *alveolata* were inspected individually. There were 104 low complexity sequences (removed) and one sequence representing *Perkinsus marinus* (removed).
- Hits in the class *viridiplantae* were inspected individually. They were exceptionally good hits and may represent remainders of plant powder, which is fed in *C. marinus* laboratory cultures. These sequences were removed.
- Hits in the class *ophistokonta* were inspected individually. They were all sequences of highly conserved genes, e.g. ubiquitin, actin and histones (kept).
- Hits in the class *fungi* were inspected individually. There were 11 sequences of fungi (removed) and 105 low complexity sequences (removed).
- Hits in the class *metazoa* were kept.

(2) **Mitochondrial genome and gene clusters.** The mitochondrial genome is difficult to assemble due to its circular nature. Gene clusters are difficult to assemble, because they are repetitive. These sequences were only found in fragmented and incomplete versions within the assembly. Thus, the unmapped scaffolds were searched for fragments of the mitochondrial genome, as well as fragments of the histone gene cluster and 18S/28S ribosomal DNA gene clusters. First, sequences from other insects served as a query against the unmapped scaffolds in a blastn search. Then, the obtained fragments from *C. marinus* served as queries in subsequent blastn search rounds for more overlapping fragments. When no additional fragments were found, the mitochondrial genome, the histone gene cluster and the 18S/28S rDNA gene clusters were assembled manually from the obtained fragments. The mitochondrial genome was submitted as one scaffold under project PRJEB8339, the histone gene cluster (ENA accession: LN833886) and the 18S/28S rDNA gene cluster (ENA accession: LN833602) had to be submitted separately. The arrangement of the mitochondrial genome and of the histone gene cluster are given in Extended Data Fig. 10. Finally, the assembled mitochondrial genome, the histone gene cluster and the 18S/28S rDNA gene clusters were searched again against all unmapped scaffolds in a blastn search. All unmapped scaffolds which had a full-

length hit against one of the above sequences were removed from the set of unmapped scaffolds.

(3) **Short polymorphic variants of parts of larger scaffolds.** After the first two steps of processing, the remaining unmapped scaffolds contained 25,193 sequences. To assess, how many of the unmapped scaffolds are merely polymorphic variants of parts of the large mapped scaffolds, a blastn search of the unmapped vs. the mapped scaffolds was performed. The blast hits were parsed according to identity and length of the hit (see Table S14). The results indicate, that approximately half of the unmapped scaffolds could be merely short polymorphic variants of parts of the mapped scaffolds. This suggests that the actual assembled sequence of the *C. marinus* genome may rather be around 83 Mb, which in turn implies that after subtracting these polymorphic variants close to 95% of the assembled sequence could be considered as mapped.

However, based on available data it is not possible to decide which scaffolds are polymorphic variants (alleles) and which are part of variable gene duplications, clusters and repeats. Therefore, none of these scaffolds were removed from the set of unmapped scaffolds. But as the European Nucleotide Archive (ENA) would not accept scaffolds <100bp, only the 23,687 unmapped scaffolds >= 100bp were submitted under project PRJEB8339. The full set of 25,193 unmapped scaffolds is available at *ClunioBase* (http://cluniobase.cibiv.univie.ac.at).

4 Removal of repeated edges, gap closing

The 75 mapped scaffolds of assembly CLUMA_0.5 contained 24,869 gaps, while the 25,193 unmapped scaffolds of assembly CLUMA_0.5 contained 1,880 gaps. We used a combination of *GapFiller*[9] (version: GapFiller_v1-10_linux-x86_64) and a custom script we call *Repeated Edge Remover* (RE[2]; for source code see Supplementary File 2) to close these gaps in the assembly.

GapFiller tries to close the gaps based on paired-end sequence reads, either by extending the edges of contigs or by trying to assemble new sequence from reads which according to paired end information should fall into a gap. The software allows to trim contig ends in order to circumvent the problems caused by misassembled sequence at the contig ends. However, this strategy is in many cases not successful in dealing with so-called *repeated edges* (see following paragraph for definition), which often are several hundred bp long, so that trimming them from the contig ends would lead to a considerable sequence loss in case the gap is not closed successfully.

A *repeated edge* is given when the sequences to the left and to the right directly adjacent to the gap are largely identical. In most cases, repeated edges are assembly errors which occur if two contigs are detected to be neighboring in the scaffolding process, but cannot be merged because the contig ends have two different variants of a polymorphic sequence. In assembly CLUMA_0.5 in about half of the cases, the scaffolding algorithm detected that the repeated edges are overlapping based on paired-end information, but it could not match the two edges due to the polymorphism. In these cases, only a single N was inserted between the repeated edges. In cases with larger gaps, repeated edges could represent truly repeated sequence that cannot be fully assembled and therefore contains a gap. To assess this possibility, we chose 15 gaps ranging from 2 bp to 2084 bp in size. We designed PCR primers to span the gap, PCR amplified across the gap and then did Sanger sequencing of the PCR products. In all 15 cases, there was no repeated sequence in the PCR product and gel electrophoresis did not give any indication for multiple bands, i.e. for (polymorphic) sequence duplications. Given this observation, the decision was made to close gaps with repeated edges with a custom script (*Repeated Edge Remover*, RE[2]).

Briefly, RE^2 identifies repeated edges based on a k-mer search: A short k-mer to either side of the gap is searched in the sequence on the respective other side of the gap (scheme C, panel A). The size of the k-mer and the size of the searched subject sequence on the other side of the gap are user-defined. RE^2 also allows to specify mismatches or shifts of the k-mer, to account for cases in which the search k-mer should be slightly polymorphic. If both k-mers at the edge of the gap are detected on the respective other side of the gap, they delimit the repeated edges. The repeated sequences to both sides of the gap are then subject to a global alignment (scheme C, panel B). If the alignment meets a user-defined threshold with respect to sequence identity (in our case 0.95), the gap is closed by arbitrarily deciding for one version of the polymorphic sequence (scheme C, panel C).

Scheme C

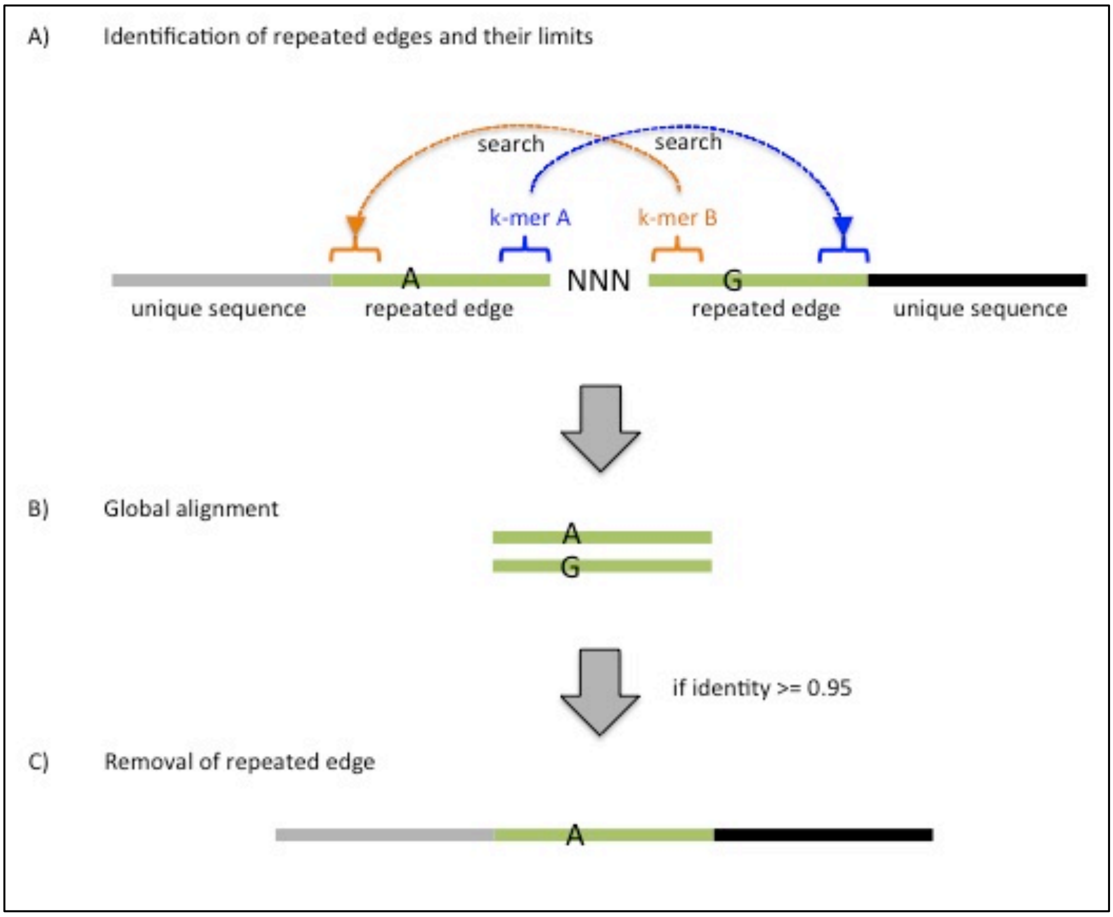

The gaps in assembly CLUMA_0.5 were closed by alternatingly running *GapFiller* and RE^2 on the scaffolds. After two initial rounds of RE^2, eight iterations of *GapFiller* were applied, each round of *GapFiller* followed by another round of RE^2. In the initial two runs of RE^2 as well as for the RE^2 runs after the first 4 rounds of *GapFiller*, RE^2 was set to assess gaps of up to 1 kb in size. Repeated edges were searched with k-mers of 20 bp, allowing 2 mismatches in the k-mer and two shifts of the k-mer if no match was found in the first place. K-mer search was restricted to 3 kb to either side of the gap and gaps were only closed if the identity of the repeated edges was >= 95%. For the last four rounds of RE^2 gaps of up to 3kb were assessed, the other parameters remaining unchanged.

GapFiller was run with all three sequencing libraries that were previously used in the assembly process. Parameters were set so that gaps were closed with reads which had a minimum overlap of 50 bp with the contig end (-m 50), coverage for sequence extension needed to be at least 2-fold (-o 2) and a minimum of 50% of the reads needed to lead to a single base extension (-r 0.5). For closing a gap, the contig ends had to overlap by at least 20 bp (-n 20) and the difference between the size of the gap and the gap-closing sequence could not be larger than 500 bp (-d 500). *GapFiller* was allowed to trim a maximum of 15 bp at the end of contigs if gap-closing was not possible (-t 15). In order to avoid the continuous trimming of sequence or the continuous insertion of large amounts of sequence by sequence extension at gaps which simply could not be closed, the parameters for *GapFiller* were changed for the last four rounds in order to disable trimming (-t 0) and to limit the maximum difference of gapsize and gap-closing sequence to 50 bp (-d 50). All other parameters remained unchanged.

The first two iterations of RE^2 already closed 14,382 gaps in the mapped scaffolds (57.8 %). After the process was finished 22,716 gaps in the mapped scaffolds were closed (91.3 %). The total sequence (mapped and unmapped scaffolds) shortened by 6.4 % (see Table S11).

Finally, to assess again if we had erroneously removed many tandem gene duplications by repeated edge removal, we plotted the average read coverage of the pooled-sequencing data of the *Jean* strain in in 100bp windows every 50 bp along the reference sequence, giving a crude estimate of copy number variation (CNV) along the sequence. If true duplications had been removed wrongly by

repeated edge removal, this would result in peaks of CNV of 2x or higher coverage along the sequence. We removed 22,716 gaps in the mapped super-scaffolds. However, in the mapped super-scaffolds we only find 1,537 peaks in CNV, where the average local coverage exceeds 1.75x average genome-wide coverage. This suggests that at most 6.7% of the closed gaps were associated with true duplications that should not have been removed. But notably, the observed peaks in CNV may also stem from other steps of the assembly process.

5 Revision of the genetic linkage map

On the 75 mapped super-scaffolds of the final assembly CLUMA_1.0 there were 3,386 male informative and 2,275 female informative markers. Thus, compared to the originally published linkage map[1] marker density was about 10-fold higher. As the RAD tags were based on the same mapping family, the original linkage map could be refined and revised (see Extended Data Fig. 2; Fig. 1a). New marker groups, which were either introduced into gaps or which replaced incorrect previous marker groups (for which the pattern of inheritance had not been detected correctly with Amplified Fragment Length Polymorphisms in the original study), were named so that the previous system of marker pattern names would be preserved. For example, if between the previous marker groups 1-M4 and 1-M5 a newly resolved recombination event was introduced, the intermediate marker pattern would be named 1-M4.1. The following changes were made to the original genetic linkage map (beyond the mere insertion of additionally resolved recombination events):

- RAD sequencing gave no evidence for the marker groups 1-M1 and 1-M2, 1-M18, 2-M21, 3-M1 and 2-F20. These marker groups were at the ends of the linkage groups and were previously supported only by one or two AFLP bands[1]. Probably these AFLP bands had single mis-scored individuals, which lead to the false assignment of non-existent marker groups. These marker groups were removed from the genetic linkage map.
- In the absence of marker group 3-M1, the location of the marker groups 3-M3 and 3-M2 are reversed.
- There is evidence for an additional marker group at the end of the female informative map of linkage group 2. This group was introduced as new marker group 2-F0.
- The marker groups 1-M4 and 1-M5 were found not to exist with the previously reported marker pattern (which was only supported by single AFLP markers). These marker groups were replaced with the revised marker groups 1-M3.1 to 1-M3.3.
- Marker group 3-M6 was found not to exist with the previously reported marker pattern. Marker group 3-M7 was slightly misplaced, as its pattern was

incomplete. Both marker groups were replaced with the revised marker groups 3-M5.1 to 3-M5.5.

- Marker group 3-M9 was found not to exist with the previously reported marker pattern. It was replaced and further resolved with the revised marker groups 3-M8.1 to 3-M8.6. Marker group 3-M8.2 is not given, as the respective recombination event is not resolved.
- The recombination events leading to 2-F7 and 2-F8 were found to be reversed in their order, due to the fact that a single individual in single AFLP band was previously mis-scored. As a consequence, marker group 2-F7 does not exist with the previously reported marker pattern and was replaced with marker group 2-F6.1.
- Marker groups 2-F16 to 2-F19 were originally separated by seven recombination events. However, a double recombination event, which had support by three AFLP markers, did not get support in RAD sequencing. Thus, only five recombination events remain, one of which is not resolved, as the informative backcross individual did not have sufficient coverage in the RAD sequencing experiment. As a consequence of the revisions, marker groups 2-F17 and 2-F18 were replaced with the marker groups 2-F16.1 to 2-F16.3. Marker group 2-F16.1 is only resolved to the extent that is must happen between two super-scaffolds.
- There is no evidence for the previously reported double recombination event from marker group 2-M16 to 2-M18. The distance between these two marker groups shrinks from four recombination events to two.
- The exact location of the recombination event from 1-M16 to 1-M17 is not resolved on the current map, as the informative backcross individual did not have sufficient coverage in the RAD sequencing experiment. Therefore, it is not shown in Fig. 1 and Extended Data Fig. 2.
- The recombination events from 3-M4 (over 6 intermediate steps) to 3-M5 were further resolved, but not completely. For one step the informative backcross individual did not have sufficient coverage in the RAD sequencing experiment.

These changes led to a reduction of the length of the genetic map to 144.45 cM for the male informative and 140.75 cM for the female informative map respectively. This results in two estimates of genome length of 150.58 cM or 146.87 cM respectively, according to method 4 in [10].

6 Larval RNA sequencing

For larval RNA sequencing, two sets of 80 third instar larvae of the *Por* and *Jean* strains respectively were snap-frozen in liquid nitrogen. RNA was extracted using the RNeasy Plus Mini Kit from Qiagen. Total RNA was checked for integrity on a 2100 Bioanalyzer with the RNA 6000 Nano Kit from Agilent. Total RNA was subject to one round of enrichment for mRNA with the Dynabeads® mRNA Purification Kit from life technologies. After mRNA enrichment, the RNA was run on a 2100 Bioanalyzer with the RNA 6000 Pico Kit from Agilent. The remaining fraction of rRNA was estimated to 14% for the *Por* strain and 19% for the *Jean* strain. Then RNA was fragmented by incubating it for 3 min at 94°C in fragmentation buffer, containing 200 mM TrisOAc (pH = 8.2), 500 mM KOAc and 50 mM MgOAc in DEPC-treated water. Fragmented RNA was cleaned up on a RNeasy spin column and RNA amount and quality were checked again on a 2100 Bioanalyzer with the RNA 6000 Pico Kit from Agilent. Then first strand cDNA was synthesized with the SuperScript® VILO cDNA Synthesis Kit from life technologies. Remaining dNTPs were removed with a MiniQuick Spin Column for DNA from Roche. Second-strand cDNA was synthesized by adding dATP, dCTP, dGTP and dUTP (final concentration: 0.2 mM), 2nd strand buffer, DNA Polymerase I (final concentration: 0.27 U/µl), DNA ligase (*E. coli*, final concentration: 0.06 U/µl) and RNAse H (final concentration: 0.01 U/µl) to the clean first-strand cDNA and incubating the mixture for 2h at 16°C. Double-stranded cDNA was cleaned up with the MinElute Reaction Cleanup Kit from Qiagen. A strand-specific Illumina sequencing library was then prepared by CSF Next Generation Sequencing facility of the Vienna Biocenter. Each sample was sequenced on a single lane of a Illumina HiSeq 2000.

7 Gene prediction with SNAP

SNAP was run with the parameter set for *Apis mellifera*, as parameters for *C. marinus* were not available for SNAP and in preliminary trials the *A. mellifera* parameter set lead to more predictions and more accurate predictions on the *C. marinus* reference genome than other available parameter sets.

Running SNAP with parameters for *A. mellifera* produced a large number of small and probably spurious gene predictions. There were 7,165 SNAP-predicted gene models without any protein or transcript support. However, there were still 3,224 gene models for which the SNAP prediction was the only evidence considered by MAKER and which were – independent of MAKER – found to have protein or transcript support (see Table S1). Therefore, gene prediction with SNAP was enabled despite the cost of numerous spurious predictions.

Supplementary Notes

1 Completeness of the reference genome and estimated gene numbers

Even though the *C. marinus* reference genome is very small (85.6 Mb), it does not seem to be characterized by a reduced number of genes or technical incompleteness. The 14,041 supported gene models are well in the range of gene numbers reported *Drosophila melanogaster* (BDGP 5, version 75.546: 15,507 genes) and *Anopheles gambiae* (AgamP3, version 75.3: 13,460 genes).

We also assessed completeness of the reference genome with the Core Eukaryotic Genes Mapping Approach (CEGMA)[11]. CEGMA reports that of 248 highly conserved eukaryotic genes it finds 240 complete and 3 partial sequences in the *C. marinus* reference assembly, leading to an estimate of 97.98% completeness. In order to find out which of the 248 eukaryotic clusters of orthologous genes (KOGs) are not found in the *C. marinus* reference genome, we investigated which KOGs are missing in the set of *C. marinus* orthologs as reported by CEGMA. Interestingly, nine KOGs were missing in the dataset (KOG0209, KOG0276, KOG0462, KOG0477, KOG0871, KOG0948, KOG0960, KOG0969, KOG1123). We then BLAST searched the corresponding nine *D. melanogaster* orthologous proteins against the *C. marinus* reference genome (tblastn) and found that seven of them were clearly present in the reference sequence, in each case indicated by a BLAST hit with an e-value of 0.00 covering more than 80% of the sequence. This BLAST result would leave only KOG0871 and KOG960 unidentified in the *C. marinus* reference genome. Orthologs of these KOGs are found in the *C. marinus* transcript datasets, indicating that *C. marinus* has not lost these genes, but that they are erroneously missing from the *C. marinus* reference genome assembly. This suggests a revised estimate of completeness of the assembly of 246/248 genes or 99.2 %.

In order to get a second estimate besides the slightly inconsistent CEGMA report, we searched the 75 mitochondrial ribosomal proteins (mRps) from *D. melanogaster* as published by Marygold et al.[12] in the *C. marinus* predicted protein dataset and reference genome. We identified all 75 genes based on reciprocal best BLAST hits or manual annotations (Table S2), underscoring that

the *C. marinus* protein dataset and reference genome sequence is close to complete.

During manual curation of the annotations we inspected all gene models in the QTLs, i.e. in approximately 10% of the reference sequence. Within these regions we found roughly 100 chimeric annotations and clusters of closely related genes that needed to be split into approximately 300 independent gene models. Extrapolating from these findings, we can expect that approximately 1,800 genes are still "hidden" in chimeric annotations within the uninspected parts of the reference genome. This expectation was underlined by the assessment of the mitochondrial ribosomal proteins (mRps), where 14 of the 75 genes were found in chimeric gene models (Table S2). The high fraction of chimeric gene models and mis-annotated gene clusters may be due to the small size of the genome. In many regions of the genome the genes are densely packed and their UTRs overlap (Extended Data Fig. 3a), which can produce chimeric sequences during transcript assembly, and these may misguide the MAKER2 annotation pipeline.

2 Genome evolution in dipterans

The *C. marinus* genome is currently the smallest sequenced insect genome. Chironomids originated 231 to 308 million years ago[13] and comparisons between the three distantly related groups can provide insights into basic patterns of genome evolution.

The genomes of *Polypedilum vanderplanki* (104 Mb; scaffold N50: 229 kb)[14] and *Belgica antarctica* (90 Mb; scaffold N50: 98 kb)[15] are similarly sized, and flow cytometry estimates for 25 chironomid species[16] suggest that chironomid genomes are generally compact. The *C. marinus* reference genome is highly contiguous (scaffold N50: 1.9 Mb) and largely mapped, making chironomids the third dipteran subfamily with a reference genome for which >90% of the chromosomes are reconstructed. The other two subfamilies are drosophilid flies, represented by five genomes including that of *Drosophila melanogaster*, and culicid mosquitoes, represented by the genome of *Anopheles gambiae* (Extended Data Fig. 3b).

In order to estimate the position of centromeres in *C. marinus* chromosomes, we estimated genetic diversity (θ), *i.e.* the amount of genetic variation at a given locus, and short-range linkage disequilibrium (LD; measured as r^2), i.e. the association between nearby genetic variants, from pooled-sequencing data of 300 field-caught individuals of the *Jean* strain. Plotting these measures along the chromosomes shows characteristic signatures of elevated LD and reduced genetic diversity at the telomeres and centromeres (Extended Data Fig. 3c), as is observed in other species[17,18]. Just as *A. gambiae* and similar to *D. melanogaster*, *C. marinus* has one telocentric and two metacentric chromosomes, resulting in five chromosome arms, which we called 1, 2L, 2R, 3L and 3R (Table S3). Comparison of the chromosomal locations of 5,388 putatively orthologous genes identified homologs to four of the *C. marinus* chromosome arms based on the largest fraction of shared genes (Extended Data Fig. 3d-f and 4a, Table S3). In the three species, homologous chromosome arms occur in different combinations within chromosomes, a phenomenon commonly observed in dipterans[19-21].

Chromosome arm 2L of *C. marinus* is homologous to the X chromosome of *D. melanogaster* and *A. gambiae*. However, *C. marinus* does not have distinct sex

chromosomes[22], but a ZW-like sex-linked locus on chromosome 1[1]. Thus, sex determination in *C. marinus* does not employ sex chromosomes, the autosomal sex determining locus is not linked to the X chromosome homolog and sex determination follows a ZW like system. This uncommon scenario underscores the idea that chironomids may be interesting objects to study the evolution of sex determination[23].

Only chromosome arm 3L of *C. marinus* is strongly conserved in gene content between *C. marinus*, *A. gambiae* and *D. melanogaster* (Extended Data Fig. 3d-f), suggesting specific constraints on rearrangements of this chromosome arm. Overall, synteny between *D. melanogaster* and *A. gambiae* is higher than the synteny of both to *C. marinus* (Extended Data Fig. 3d-f and 4a). This suggests that chromosomal rearrangements are more common in the lineage leading to *C. marinus*, a phenomenon we looked at in more detail (see Supplemantary Note 3).

3 An elevated rate of chromosomal rearrangements in the lineage leading to *C. marinus*

Genome-wide synteny comparison revealed that gene content of chromosome arms is more conserved between *A. gambiae* and *D. melanogaster* than between the more closely related *A. gambiae* and *C. marinus,* suggesting that the lineage leading to *C. marinus* has an elevated rate of chromosomal re-arrangements. An analysis of conserved microsynteny blocks between *C. marinus*, *D. melanogaster* and *A. gambiae* supports this view (section 3.1 below). It is also in line with the observation that polymorphic chromosomal rearrangements are very common in *C. marinus*, as is suggested by non-recombining regions in mapping families and non-pairing regions in polytene chromosomes (section 3.2), as well as by analysis of pooled sequencing data from *C. marinus* populations (section 3.3).

3.1 Analysis of microsynteny blocks

Considerations on microsynteny were limited to a set of 5,388 genes, for which 1:1:1 putative orthology between *C. marinus*, *A. gambiae* and *D. melanogaster* was suggested by reciprocal best blast hits among all three species. This dataset served to calculate the fraction of genes that occurs in microsynteny blocks between species pairs. If for two adjacent orthologs in *C. marinus* the respective orthologs in *A. gambiae* are also adjacent, this is counted as two genes that are in a microsynteny block. Computing the blocks for the 5,388 genes with 1:1:1 orthology provided the fraction of genes in microsynteny blocks for the three species pairs. The fraction of genes in microsynteny blocks was 0.2326 for the *C. marinus* – *A. gambiae* comparison (“CA”), 0.1555 for the *C. marinus* – *D. melanogaster* comparison (“CD”) and 0.2318 for the *A. gambiae* – *D. melanogaster* comparison (“AD”).

Based on the fraction of pairwise microsynteny, we estimated the fraction of microsyntenic conservation allocated to the specific branches in the phylogenetic tree of *C. marinus*, *A. gambiae* and *D. melanogaster*, by solving the system of equations in Extended Data Fig. 4b. A direct comparison of all three branches is hindered by the fact that the exact divergence times of the species

are unknown, but the branches of *A. gambiae* and *C. marinus* had the same time t_1 of independent evolution (Extended Data Fig. 4b). Nevertheless, conservation of microsynteny in the *A. gambiae* branch is 1.5-fold the conservation in the *C. marinus* branch. This suggests, that in the lineage leading to *C. marinus*, chromosomal rearrangements are more common.

A crude simulation can provide an estimate of how much more chromosomal rearrangements must take place in the branch leading to *C. marinus* to yield the observed pattern. To this end we simulate 1,001 genes along a chromosome (1,000 connections) and then randomly break the links between neighboring genes (Extended Data Fig. 4c). We then monitor the fraction of genes in microsynteny blocks as a function of the number of breaks (Extended Data Fig. 4d). As it requires two breaks for a gene to drop out of a microsynteny block and as breaks can hit the same position several times, the decrease in microsynteny is not linear. From the observed microsynteny fraction occurring on each branch, we can then estimate how many breaks occurred along that branch. The simulation suggests that breaks (and thus the number of rearrangements) in the branch leading to *C. marinus* are 1.47x more frequent than breaks in the branch of *A. gambiae*. In the branch of *D. melanogaster* the estimated number of breaks is almost equal to the number in the branch of *C. marinus* (1.003x), although the branch of *D. melanogaster* is certainly longer ($t_1 + 2*t_2$ for *D. melanogaster* vs. t_1 for *C. marinus*; compare Extended Data Fig. 4b).

The monophyletic origin of chironomids, including *C. marinus*, is estimated to 231 to 308 million years ago[13]. The estimated divergence time of *D. melanogaster* and *A. gambiae* is 215 to 294 million years ago[24]. For the unknown times t_1 and t_2 in the phylogenetic tree, combining these estimates implies that t_1 can range from 231-294 million years and t_2 from 0 to 63 million years, the actual possible range of t_2 depending on t_1. Within these ranges, there are many possible combinations of t_1 and t_2 that would make the frequency of rearrangements along the *D. melanogaster* branch equal the frequency of rearrangements along the *A. gambiae* branch (e.g. t_1 = 231 million years and t_2 = 54 million years). Such a scenario would imply that an elevated frequency of rearrangements is likely specific to the lineage leading to *C. marinus*, possibly specific to chironomids.

3.2 Genetic linkage maps and polytene chromosomes highlight large non-recombining regions

Based on the refined linkage map of the *C. marinus* genome, both male and female informative markers served to place and orient the 75 mapped super-scaffolds on the genetic linkage map (Extended Data Fig. 2). This allowed the reconstruction of the three chromosomes of *C. marinus* (Fig.1a).

There is a large region at the end of linkage group 2 in which no recombination events were observed in the male and the female, suggesting a large heterozygous chromosomal rearrangement may have been present in both backcross parents. Additionally, half of linkage group 3 does not show any recombination in the F1 hybrid father, while recombination is limited to the middle of that region in the backcross mother. This may also point to a large inversion or other rearrangement, maybe with different levels of complexity in the two individuals. Further small regions with low recombination are found in the first half of linkage group 1.

Generally, these regions with low recombination coincide with regions that were difficult to assemble. These difficulties were often due to ambiguous connections between scaffolds (see for example the super-scaffolds 47A to 47K, which all received the identifier "47", as they were all part of a connected scaffold network). These ambiguous connections may indicate that these regions harbor complex sets of rearrangements, which suppress recombination. Notably, the non-recombining region that comprises super-scaffolds 47A- 47K largely corresponds to chromosome arm 2R (Extended Data Fig. 2; Table S3), which could not be assigned clear homology in other dipterans, as genes found in the different chromosome arms of other dipterans occur at similar frequencies (Extended Data Fig. 3e,f and 4a). This underscores the idea of frequent chromosomal rearrangements in this particular chromosome arm.

These findings are further backed by the published description of the polytene chromosomes of *C. marinus*[22]. The polytene chromosomes have been named I, II and III. Polytene chromosome I carries the nucleolus organizer region (NOR), i.e. the ribosomal DNA clusters, fragments of which are found on chromosome 1 of our reference assembly. Thus, the polytene chromosomes II

and III must correspond to the chromosomes (or linkage groups) 2 and 3 in the reference assembly, although we do not know in which combination. However, large chromosomal inversions are frequently found in both polytene chromosomes II and III[22], fully consistent with the large non-recombining regions that we observe in both linkage groups 2 and 3.

The polytene chromosomes of other chironomids have also been found to show many (polymorphic) chromosomal rearrangements[21,25], suggesting the phenomenon of an elevated rate of chromosomal rearrangements may not be limited to *C. marinus*, but may affect chironomids in general.

3.3 Detection of inversions and deletions from NGS data

In order to further substantiate the finding of frequent chromosomal rearrangements for *C. marinus*, we screened the available pool-sequencing data of the *Por* and *Jean* strains (see population genomic analyses for details) for large insertion-deletions or inversions with the multi-sample version of DELLY[26]. Detection of chromosomal rearrangements with NGS data is sensitive to errors in the reference sequence and limited to continuous reference sequence.

To meet the first problem we set strict quality criteria in that we only reported rearrangements if they had support by both seemingly malformed read pairs (i.e. the paired-end read orientation is altered by inversions or the paired-end read distance is altered by deletions/insertions) and split reads (i.e. a read is mapped to discontinuous reference sequence due to the fact that it spans an inversion or deletion breakpoint). Additionally, all reported rearrangements had to pass DELLY's default quality filter. Based on these criteria we identified 737 putative insertion-deletions (median: 2.5 kb) and 272 putative inversions (median: 76.4 kb). Basically all chromosomal rearrangements are reported to be polymorphic, i.e. both the reference arrangement and the variant arrangement have support, and mostly the frequency of the two arrangements varies between the two tested strains.

Due to the limitation of rearrangement detection to continuous reference sequence, all detected rearrangements in *C. marinus* lie within individual super-scaffolds. This implies that large chromosomal rearrangements, which go beyond the scope of individual super-scaffolds, escaped our analysis. Thus, particularly

the number and median size of inversions in the *C. marinus* genome may be much larger than reported.

4 Refined QTL analysis for circadian and lunar timing

Based on the revised linkage map with increased marker density, Quantitative Trait Locus (QTL) analysis for the differences in circadian and circalunar timing was repeated according to the original publication[1]. The timing differences of the strains are given in Extended Data Fig. 1. The revised QTL analysis does not differ in the number of detected QTLs, while the location and estimated effects of the QTLs differ slightly (Table S4).

One notable consequence of the slight changes is that now the location of one of the circadian and one of the circalunar QTLs coincide at marker group 1-M6, while previously they were separated by a few cM. This revision in the genetic architecture is important in the light of the previous finding that circadian and circalunar timing adaptations in the crossing experiment are not inherited independently, but the two traits are correlated[27]. The previously reported genetic architecture[1] was not sufficient to explain the correlation. In the same statistical test as used in the previous study (see [1]), the null hypothesis that the genetic architecture is sufficient to explain the correlation is now not rejected anymore based on the revised architecture ($p = 0.0526$ based on estimated additive effects; $p = 0.1047$ based on r^2).

The other notable difference is that the effect of the circadian QTL at 1-M6 is now estimated to be weaker than previously, while the effect of the circadian QTL at 1-M16/1-M17 is estimated to be larger (see Table S4).

Of the known putative circadian clock genes, only *timeout/timeless2* is located within the QTLs. The presence of a single putative clock gene in the QTLs is consistent with a random distribution of these genes. A previously reported *timeless3* gene in the same region[1] is a 3' fragment of the *timeless2* gene.

5 Differentiated SNPs in in the *C. marinus* timing strains

Genome-wide, there are 1,263 (0.12%) strongly differentiated SNPs (FST ≥ 0.8). Most of these SNPs are non-coding (Extended Data Fig. 5c,d), but compared to all SNPs in the genome, the strongly differentiated SNPs are slightly enriched for non-synonymous coding SNPs (19% vs. 13%). Additionally, we detected 873 strongly differentiated short indels (<30 bp; FST ≥ 0.8; Extended Data Fig. 5c,d).

For almost all SNPs with FST≥0.8, the major allele in one strain also occurs at low frequency (0.5 to 5%) in the other strain, suggesting that different adaptive timing alleles were already present in the ancestral populations as standing genetic variation. This is congruent with the fact that in QTLs C1/L1 and L2 there are no extended differentiation peaks. Such peaks would be expected if these QTLs had experienced recent hard selective sweeps involving de-novo mutations (Extended Data Fig. 5a,b).

6 Determining lunar peak phase for semi-lunar rhythms

For *C. marinus* strains with lunar rhythms, i.e. with a single emergence peak in one lunar cycle, determining the phase of the lunar peak relative to the artificial moonlight treatment in the laboratory is straightforward (compare *Vigo* and *Jean* in Extended Data Fig. 1). However, for strains with a semilunar rhythm, i.e. two peaks in one lunar cycle, it is necessary to explain why only one peak is considered and based on which criteria this peak is chosen (compare *Por*, *He* and *Ber* in Extended Data Fig. 1).

Free-running experiments are experiments in which *C. marinus* strains are first treated with artificial moonlight, but then released into conditions without artificial moonlight in order to determine the period at which their endogenous circalunar clocks run without external moonlight cues. Free-running experiments showed that the lunar rhythm in the *Jean* strain has an endogenous free-running period of 27 days, whereas the semilunar rhythms in the *Por* and *He* strains have an endogenous free-running period of 14 days or 11 days respectively[28]. In the light of these findings, we may assume that a semi-lunar rhythm consists of a “directly entrained peak”, which is set by the last effective moonlight treatment, and a “free-running peak”, which is merely a product of the short endogenous period of the lunar rhythm (11-14 days) that allows for the occurrence of a second peak in a lunar cycle (28.5 days). In strains with a lunar rhythm the “free-running peak” is absent, because the free-running period (27 days) is very close to the lunar cycle (28.5 days), so that each peak will be directly entrained by a corresponding previous moonlight treatment. Therefore, for comparison of the peak phase between strains with lunar and semilunar rhythms, we need to find out which peak is the “directly entrained peak” in a semilunar rhythm. There are two lines of evidence, which allow us to do so.

First, crossing experiments between the *Por* strain (semilunar rhythm) and the *Jean* strain (lunar rhythm) result in an F1 hybrid generation with a major peak that is intermediate in phase between the single *Jean* peak and the first peak of the *Por* strain around day 2 (compare Extended Data Fig. 1c for *Por* and *Jean* and [27] for the F1 hybrids). This suggests that the first *Por* peak is the

physiological equivalent to the single *Jean* peak, and thus it would be the "directly entrained peak".

Second, experiments by Neumann showed that lunar emergence time in *C. marinus* is already fully determined about 20 days before emergence[29]. As a consequence of that, the second peaks in the *Por*, *He* and *Ber* strains (around days 17-20; see Extended Data Fig. 1) happen too early after the previous moonlight treatment to be directly entrained by this moonlight treatment. Thus, the "directly entrained peak" in these semi-lunar rhythms must rather be the first peak (around days 1-5; see Extended Data Fig. 1), being entrained by the moonlight treatment that took place about 30 days earlier. This is fully consistent with the observations in the crossing experiment.

As a consequence of that, we compared the lunar timing differences between the *C. marinus* strains based on the first peaks of the *Por*, *He* and *Ber* strains (around days 1-5).

Supplementary References

1 Kaiser, T. S. & Heckel, D. G. Genetic Architecture of Local Adaptation in Lunar and Diurnal Emergence Times of the Marine Midge *Clunio marinus* (Chironomidae, Diptera). *PLoS ONE* **7**, e32092, doi:10.1371/journal.pone.0032092 (2012).

2 Barton, M. D. & Barton, H. A. Scaffolder - software for manual genome scaffolding. *Source code for biology and medicine* **7**, 4-4, doi:10.1186/1751-0473-7-4 (2012).

3 Baird, N. A. *et al.* Rapid SNP Discovery and Genetic Mapping Using Sequenced RAD Markers. *PLoS ONE* **3**, e3376 (2008).

4 Martin, M. Cutadapt removes adapter sequences from high-throughput sequencing reads. *EMBnet.journal* **17**, 10-12 (2011).

5 Li, H. & Durbin, R. Fast and accurate short read alignment with Burrows–Wheeler transform. *Bioinformatics* **25**, 1754-1760 (2009).

6 Li, H. *et al.* The Sequence Alignment/Map format and SAMtools. *Bioinformatics* **25**, 2078-2079, doi:10.1093/bioinformatics/btp352 (2009).

7 McKenna, A. *et al.* The Genome Analysis Toolkit: A MapReduce framework for analyzing next-generation DNA sequencing data. *Genome Research* **20**, 1297-1303, doi:10.1101/gr.107524.110 (2010).

8 Huson, D. H., Mitra, S., Ruscheweyh, H.-J., Weber, N. & Schuster, S. C. Integrative analysis of environmental sequences using MEGAN4. *Genome Research* **21**, 1552-1560, doi:10.1101/gr.120618.111 (2011).

9 Boetzer, M. & Pirovano, W. Toward almost closed genomes with GapFiller. *Genome Biology* **13**, R56, doi:10.1186/gb-2012-13-6-r56 (2012).

10 Chakravarti, A., Lasher, L. K. & Reefer, J. E. A Maximum-Likelihood method for estimating genome length using genetic-linkage data. *Genetics* **128**, 175-182 (1991).

11 Parra, G., Bradnam, K. & Korf, I. CEGMA: a pipeline to accurately annotate core genes in eukaryotic genornes. *Bioinformatics* **23**, 1061-1067, doi:10.1093/bioinformatics/btm071 (2007).

12 Marygold, S. J. *et al.* The ribosomal protein genes and Minute loci of Drosophila melanogaster. *Genome Biology* **8**, doi:10.1186/gb-2007-8-10-r216 (2007).

13 Cranston, P. S., Hardy, N. B., Morse, G. E., Puslednik, L. & McCluen, S. R. When molecules and morphology concur: the 'Gondwanan' midges (Diptera: Chironomidae). *Systematic Entomology* **35**, 636-648, doi:10.1111/j.1365-3113.2010.00531.x (2010).

14 Gusev, O. *et al.* Comparative genome sequencing reveals genomic signature of extreme desiccation tolerance in the anhydrobiotic midge. *Nature Communications* **5**, doi:10.1038/ncomms5784 (2014).

15 Kelley, J. L. *et al.* Compact genome of the Antarctic midge is likely an adaptation to an extreme environment. *Nature Communications* **5**, doi:10.1038/ncomms5611 (2014).

16 Cornette, R. *et al.* Chironomid midges (Diptera, Chironomidae) show extremely small genome sizes. *Zoological Science* **32**, 248-254, doi:10.2108/zs140166 (2015).

17 Mackay, T. F. C. *et al.* The *Drosophila melanogaster* Genetic Reference Panel. *Nature* **482**, 173-178, doi:Doi 10.1038/Nature10811 (2012).

18 Ellegren, H. *et al.* The genomic landscape of species divergence in *Ficedula* flycatchers. *Nature* **491**, 756-760, doi:10.1038/nature11584 (2012).

19 Zdobnov, E. M. *et al.* Comparative genome and proteome analysis of Anopheles gambiae and Drosophila melanogaster. *Science* **298**, 149-159, doi:10.1126/science.1077061 (2002).

20 Schaeffer, S. W. *et al.* Polytene chromosomal maps of 11 Drosophila species: the order of genomic scaffolds inferred from genetic and physical maps. *Genetics* **179**, 1601-1655 (2008).

21 Wülker, W. Basic patterns in the chromosome evolution of the genus Chironomus (Diptera). *Zeitschrift für zoologische Systematik und Evolutionsforschung* **18**, 112-123 (1980).

22 Michailova, P. A Review of the European Species of Genus *Clunio* Haliday, 1855 (Diptera, Chronomidae). *Zoologischer Anzeiger* **205**, 417-432 (1980).

23 Bachtrog, D. *et al.* Sex Determination: Why So Many Ways of Doing It? *PLoS Biology* **12**, 1899-1899 (2014).

24 Logue, K. *et al.* Mitochondrial genome sequences reveal deep divergences among Anopheles punctulatus sibling species in Papua New Guinea. *Malaria Journal* **12**, doi:10.1186/1475-2875-12-64 (2013).

25 White, M. J. D. Chromosomal rearrangements and speciation in animals. *Annual Review of Genetics*, 75-98 (1969).

26 Rausch, T. *et al.* DELLY: structural variant discovery by integrated paired-end and split-read analysis. *Bioinformatics* **28**, I333-I339, doi:10.1093/bioinformatics/bts378 (2012).

27 Kaiser, T. S., Neumann, D. & Heckel, D. G. Timing the tides: Genetic control of diurnal and lunar emergence times is correlated in the marine midge *Clunio marinus*. *BMC Genetics* **12**, 49, doi:10.1186/1471-2156-12-49 (2011).

28 Neumann, D. Die lunare und tägliche Schlüpfperiodik der Mücke *Clunio* - Steuerung und Abstimmung auf die Gezeitenperiodik. *Zeitschrift für Vergleichende Physiologie* **53**, 1-61 (1966).

29 Neumann, D. & Spindler, K. D. Circasemilunar Control of Imaginal Disk Development in Clunio marinus - Temporal Switching Point, Temperature-Compensated Developmental Time and Ecdysteroid Profile. *Journal of Insect Physiology* **37**, 101-109 (1991).

doi:10.1038/nature20151

Supplementary Figure

Supplementary Figure 1 Source image for gel lanes in Fig. 3c

Lane 1: Marker (nt); lanes 2,4,6 and 8: *Por* strain; lanes 3,5,7 and 9: *Jean* strain; lane 10: -RT control. Lanes 6 and 9 are shown as representative examples with comparable background in Fig. 3c. For quantifications local background differences are used for standardisation using the “local average” method of the ImageQuant software.

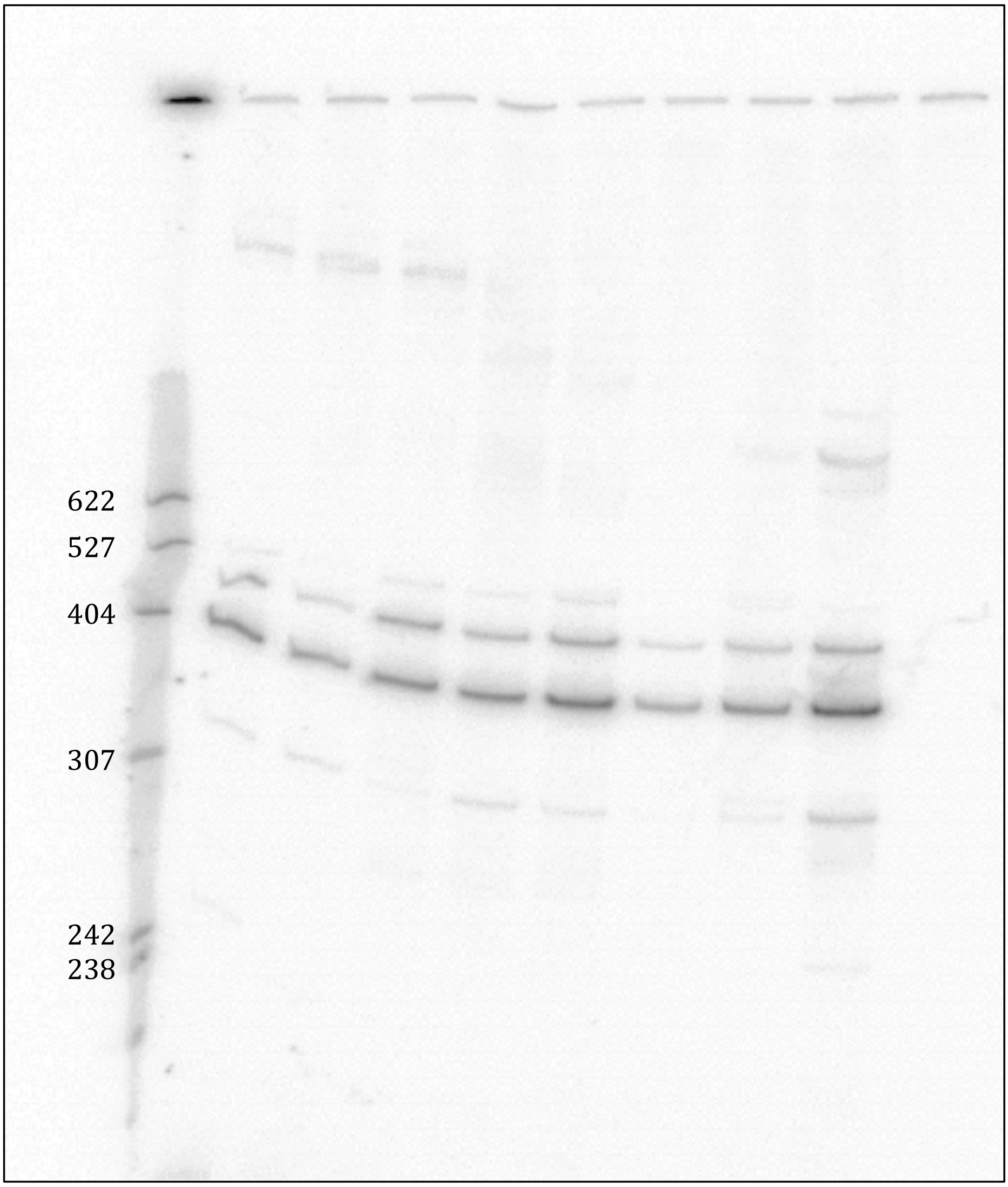

Supplement: Supplementary Information — This file contains Supplementary Tables 1-19, Supplementary Methods, Supplementary Notes, Supplementary References and a Supplementary Figure – see contents page for details. (PDF 2893 kb) [file 41586_2016_BFnature20151_MOESM28_ESM.pdf]
