# Supplementary material for: The genomic basis of circadian and circalunar timing adaptations in a midge
Source: Nature. 2016 Nov 21;540(7631):69–73. doi: 10.1038/nature20151 (PMC5133387; doi:10.1038/nature20151)
Supplement: Supplementary file 4 — PowerPoint slide for Fig. 1 [file 41586_2016_BFnature20151_MOESM25_ESM.ppt]

## Slide 1
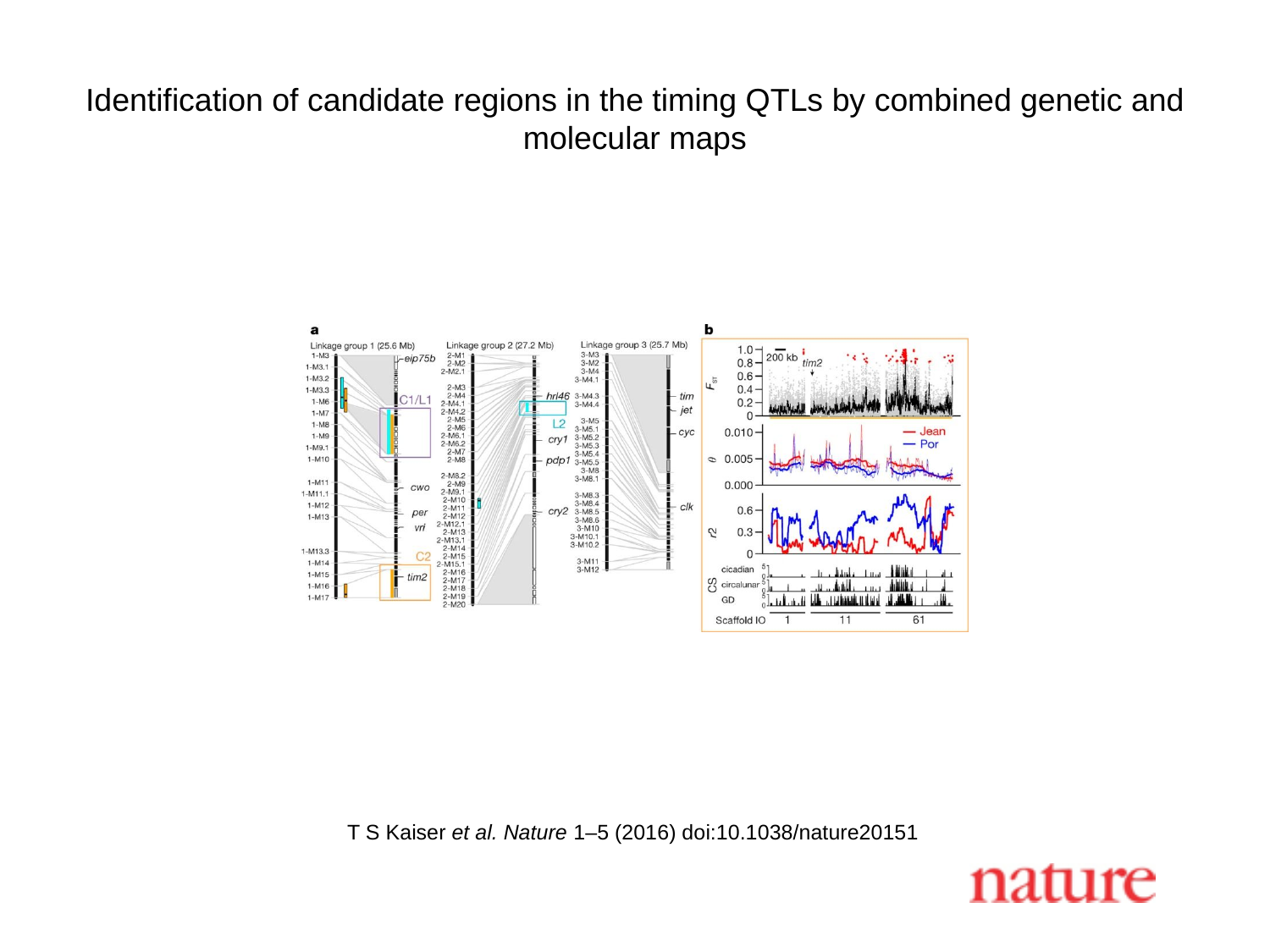

# Identification of candidate regions in the timing QTLs by combined genetic and molecular maps
T S Kaiser et al. Nature 1–5 (2016) doi:10.1038/nature20151
